# *O,S*-Acetals in a New Modification of *oxo*-Friedel–Crafts–Bradsher Cyclization—Synthesis of Fluorescent (Hetero)acenes and Mechanistic Considerations

**DOI:** 10.3390/molecules28062474

**Published:** 2023-03-08

**Authors:** Krzysztof Owsianik, Ewa Różycka-Sokołowska, Piotr Bałczewski

**Affiliations:** 1 Division of Organic Chemistry, Centre of Molecular and Macromolecular Studies, Polish Academy of Sciences, Sienkiewicza 112, 90-363 Łódź, Poland; 2 Institute of Chemistry, Faculty of Science and Technology, Jan Długosz University in Częstochowa, Armii Krajowej 13/15, 42-201 Częstochowa, Poland

**Keywords:** acenes, cyclization, *O,S*-acetals, electrophilic aromatic substitution, Friedel–Crafts–Bradsher reactions, fluorescence

## Abstract

This paper presents the use of *O,S*-acetals in a new modification of the *oxo*-Friedel–Crafts–Bradsher cyclization. In this reaction, under mild reaction conditions (25 °C), three- and four-ring fused *RO*-acenes (major) and/or *HO(CH_2_)_2_S*-acenes (minor) are formed, the latter products having never been observed before in this type of cyclization. In this way, two electronically different fluorophores could be obtained in a single cyclization reaction, one of them having strong electron donor properties (+M effect of alkoxy groups) and the other having donor-acceptor properties (+M and −I effects of the *HO(CH_2_)_2_S*-group, Hammett’s constants). Further increasing the reaction temperature, HCl concentration or prolonging reaction time, surprisingly, yielded a 2:1 mixture of *cis* and *trans* dimeric isomers, as the only products of this cyclization. The DFT calculations confirmed a greater stability of the *cis* isomer compared to the *trans* isomer. The formation of unexpected dimeric products and *HO(CH_2_)_2_S*-acenes sheds light on the mechanism of *oxo*-Friedel–Crafts–Bradsher cyclization, involving competitive O/S atom protonation in strained *O,S*-acetals and in strain-free side groups of intermediate species.

## 1. Introduction

Organic electronics and optoelectronics are relatively new fields of basic knowledge and technology, which have become a subject of interest to chemists, physicists and process engineers [1]. Therefore, a search for organic fluorescent and semiconducting materials for the construction of new-generation electronic devices, such as organic light-emitting diodes (OLEDs), organic field-effect transistors (OFETs), organic solar cells (OPVs), organic solar concentrators (OSCs), organic lasers, etc., has drawn the attention of numerous multidisciplinary joint laboratories [2]. Among aromatic hydrocarbons, linearly fused acenes are being considered as key organic compounds for achieving these goals. Anthracene and its derivatives are particularly attractive due to high thermal stability [3], relatively good solubility, low price, blue photoluminescent [4] and electroluminescent properties [5]. Many blue-light-emitting materials with an anthracene core structure [6,7,8,9,10,11,12,13,14,15,16,17,18] have been developed; however, deep blue is still in demand due to the lack of electrically and photochemically stable light-emitting materials [19,20].

In the literature, examples of intramolecular cyclizations of *o*-formyl [21], *o*-acyl [22] and *o*-carboxy [23] diarylmethanes as well as *o*-carboxy [24,25] diarylketones, leading to the required fused aromatic systems, have been described. The first two types of reactions and our present modification of the *oxo*-Friedel–Crafts–Bradsher cyclization, utilizing *O,S*-acetals, lead directly to fused aromatic hydrocarbons, while the remaining transformations require additional steps involving reductions in intermediate products, i.e., anthrones or anthraquinones, followed by aromatization of the obtained cyclic system. In addition, these reactions require harsh reaction conditions, such as high concentrations of Brønsted acids and high temperatures up to 180 °C or more, which preclude the presence of most substituents on the aromatic system [26,27,28]. Only a few examples of reactions, carried out under milder, non-aqueous reaction conditions, are known [29,30]. Our approach employs a dilute, aqueous methanolic solution (2:1) of hydrochloric acid as a strong carbocation solvating medium and room temperature, being the mildest reaction conditions ever used in these types of intramolecular, electrophilic and aromatic cyclizations [31,32,33]. These mild conditions allow for the installation of thermally and chemically sensitive functional groups on aromatic systems and, thus, the *oxo*-Friedel–Crafts–Bradsher cyclization gives rise to highly substituted, fused aromatics, as we demonstrated in this study.

**Scheme 1:**
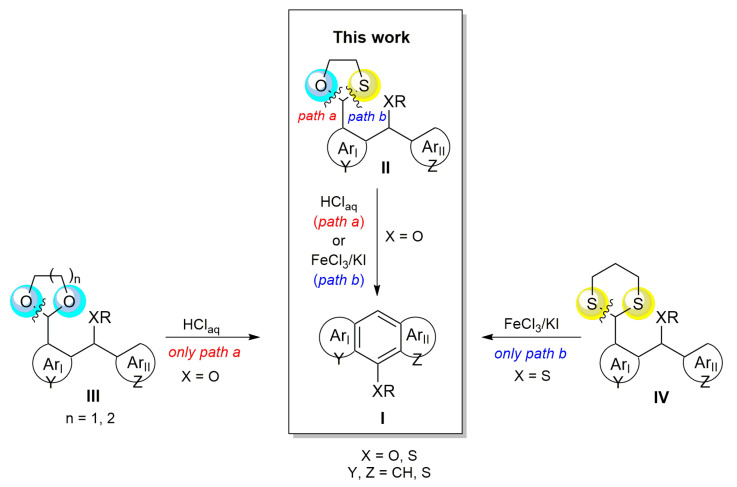
The *hetero*-Friedel–Crafts–Bradsher cyclization of *O,O*-, *O,S*- and *S,S*-acetals.

Earlier, we obtained hetero (XR = OR, SR)-substituted acenes **I** via cyclization of diarylmethanol derivatives, i.e., *ortho*-*O,O*-acetals **III** (path a, Scheme 1) via *oxo*-Friedel–Crafts–Bradsher cyclization [32,33,34] or *S,S*-dithioacetals **IV** (path b, Scheme 1) via the *thio*-Friedel–Crafts–Bradsher cyclization [3]. In both *heter*o-Friedel–Crafts–Bradsher cyclizations, a new benzene ring, fused to two other (hetero)aromatic moieties, ArI and ArII, is formed in the acene **I**. It is worth noting that the cyclization of *O,O*-acetals **III** took place only in the Brønsted acid aqueous solutions and did not occur under anhydrous conditions in the presence of Lewis acid (FeCl_3_/KI). On the other hand, the cyclization of *S,S*-dithioacetals **IV** proceeded exclusively in the presence of FeCl_3_/KI in an organic solvent solution.

This differentiated behavior of *O,O*-acetals and *S,S*-dithioacetals is due to the greater hydrolytic susceptibility of acetal C-O bonds than dithioacetal C-S bonds towards relatively dilute Brønsted acids and a lack of reactivity of the FeCl_3_/KI system towards C-O bonds. In this way, suitable reaction conditions can be selected for the preservation of sensitive substituents on the aromatic system.

In the present study, we employed *O,S*-acetals (1,3-oxathiolanes) **II,** which possess C-O and C-S bonds, as precursors of three- and four-ring fused aromatics **I**. Both bonds are cleaved under different reaction conditions, yielding carbocation intermediates that are active in the new *oxo*-Friedel–Crafts–Bradsher cyclization modification (Scheme 1). The importance of *O,S*-acetals, as carbocation-equivalent reagents for carbon–carbon bond formation and as protecting groups for carbonyl compounds, has been well documented. Generally, *O,S*-acetals are prepared via condensation of carbonyl compounds with mercaptoalcohols in the presence of protic acids [35,36,37]. Among *O,S*-acetals, 1,3-oxathiolanes and 1,3-oxathianes have long been used [38,39]. They are considerably more stable than the *O,O*-acetals under acidic conditions and easier to remove than *S,S*-acetals [40]. Mechanistic studies on the rate of acid-catalyzed cleavage show that *O,S*-acetals have a stability that lies between *S,S*-dithioacetals and *O,O*-acetals [41].

## 2. Results and Discussion

### 2.1. Synthesis

**Scheme 2:**
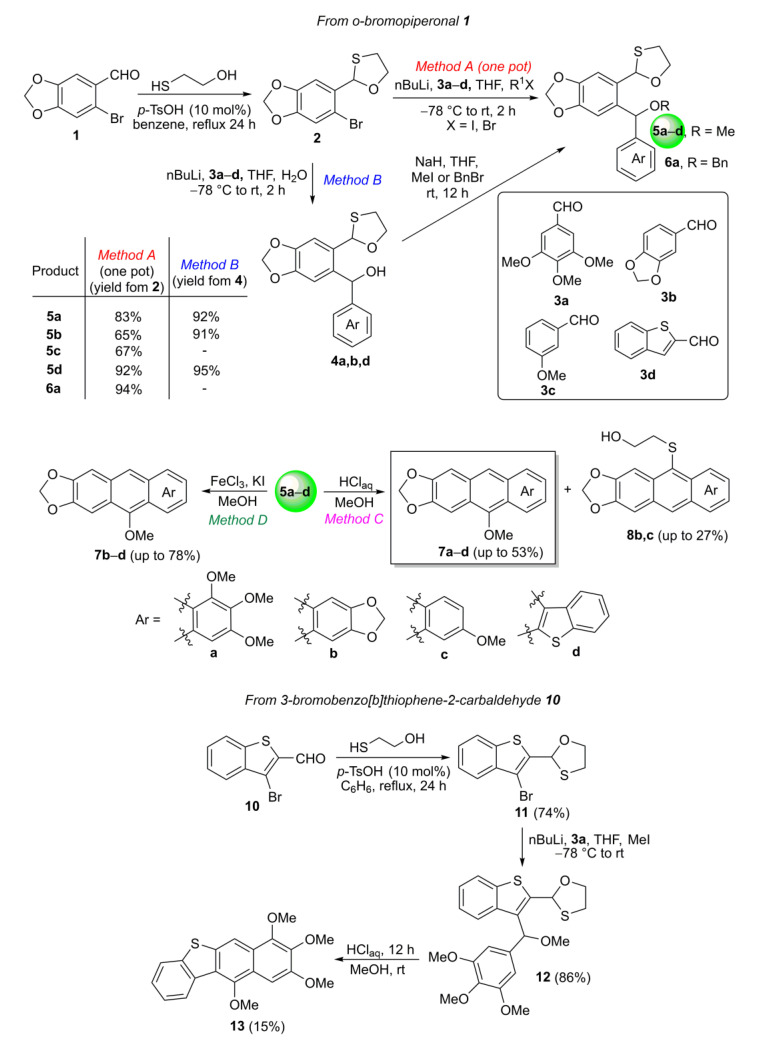
Synthesis *RO*-acenes **7a**–**d** and **13** (major) and *HO(CH_2_)_2_S*-acenes **8b**,**c** (minor) via the *oxo*-Friedel–Crafts–Bradsher cyclization. The yields given refer to products of the reactions on about a 0.3 g scale and purified by column chromatography.

The synthesis of a series of three- and four-ring fused aromatics **7** and **13** was based on the new modification of the *oxo*-Friedel–Crafts–Bradsher reaction [3], employing cyclization of *O,S*-acetals **5** and **12** (Scheme 2). The strategy of the synthesis involved is as follows: (1) protection of the aldehyde group in *ortho*-bromo aromatic aldehydes **1** and **10** with 1,2-mercaptoethanol to give *O,S*-acetals **2** and **12**, (2) the Br/Li exchange reaction in the latter followed by condensation with aromatic aldehydes **3** to afford diarylmethanols **4**, (3) protection of the hydroxyl group in **4** with methyl or benzyl halides to obtain diarylmethyl or benzyl ethers **5** and **6** to avoid the formation of lactones from the reaction of free hydroxyl with aldehyde groups and (4) acid-driven cyclization of **5** and **12** to the corresponding acenes **7** and **13** (Scheme **2**).

Turning to a more detailed discussion of this synthesis, the first *O,S*-acetalization step was performed with 2-mercaptoethanol, catalytic amount of *p*-TsOH and the corresponding *ortho*-bromo aromatic aldehydes **1** or **10** in refluxing benzene using the Dean-Stark trap to remove water (24 h). The crude reaction mixture was purified with column chromatography to give a colorless solid of *o*-bromo *O,S*-acetals **2** in 80% yield and **11** as colorless oil in a 74% yield (Scheme 2). *Ortho-lithiation* of 2 in THF at low temperature followed by the reaction with different aromatic aldehydes 3 led to diarylmethanols **4** in up to an 88% yield, which were next converted to the corresponding ethers **5**, **6** with methyl or benzyl halides (Method B, Scheme 2). Product **6a** was obtained in a 94% yield; however, it decomposed on silica gel during attempts of purification and, therefore, was not used in further transformations. It should be noted that the last two steps can be carried out as a one-pot procedure, which was found to also be effective in the synthesis of ether **12** directly from *o*-bromo *O,S*-acetal **11** (Method A, Scheme 2). The synthesized alcohols **4** and ethers **5**, **6** and **12** were obtained as inseparable mixtures of two diastereoisomers, which were used in further reactions. However, in the case of *o*-(*O,S*-acetalaryl)arylmethyl methyl ether **5d**, the major and minor diastereoisomers were successfully separated by column chromatography over silica. In the ^1^H NMR spectra, characteristic singlets at around 5.5 ppm due to OCHS, multiplets at around 5.3 ppm from 1,3-dioxolane ring and singlets at around 6.5 ppm from the dibenzylic proton were observed for the discussed compounds.

*Cyclization reaction.* As mentioned, in the case of *O,O*-acetals, especially five- and six-membered ones, the cyclization to acenes proceeded only with Brønsted acids in an aqueous media (path a, Scheme 1) [31,32,33] with the cleavage of C-O acetal bonds, while with six-membered *S,S*-acetals, the cyclization occurred only with Lewis acids in anhydrous media with the cleavage of the C-S dithioacetal bonds (path b, Scheme 1). One of the reasons for this differentiated behavior is the lower electron density on the bigger sulfur atom than on the oxygen atom and the lower electronegativity of the former, which means that the 1,3-dithiane sulfur atoms in moderately concentrated mineral acid aqueous solutions at room temperature do not undergo an effective protonation, with the consequence that they also do not undergo apparent hydrolysis through the intermediate benzyl-type carbocations that are required for the *thio*-Friedel–Crafts–Bradsher cyclization to occur.

We discovered that a different situation exists in strained five-membered *O,S*-acetals, in which both C-O and C-S bonds can be cleaved in the presence of mineral acids (HCl), especially when the reaction conditions were intensified (higher HCl concentration, higher temperature, longer reaction time). Therefore, in this study, we installed *ortho*-*O,S*-acetal moiety on one of the aryl groups in **5** and **12** to benefit from the ability to cleave both C-O and C-S bonds under different reaction conditions (Method C and D, Scheme 2) and to study the mechanism of this electrophilic modification.

**Figure 1:**
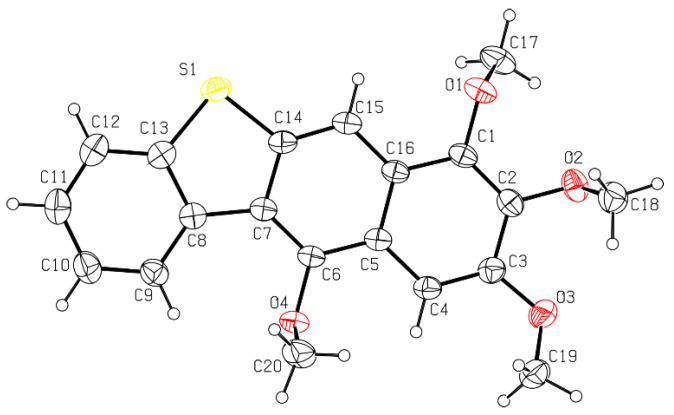
A view of molecule **13,** showing the atom numbering scheme and displacement ellipsoids drawn at the 30% probability level. H atoms are shown as small spheres of arbitrary radii.

Thus, having in hand *o*-(*O,S*-acetalaryl)arylmethyl methyl ethers **5** and **12***,* we started the investigation of the *oxo*-Friedel–Crafts–Bradsher cyclization with these substrates (Table 1). The cyclization was performed with aqueous solution of hydrochloric acid in methanol (r.t., 72 h, Method C, Scheme 2) and in the presence of the FeCl_3_/KI in methanol under anhydrous conditions (65 °C, 12 h, Method D, Scheme 2). The latter conditions gave better yields (up to 78%) of fused *RO*-acenes **7** while the former ones delivered up to 53% yields and required longer reaction times. Interestingly, the yield of the four-ring acene **13** was 62% when the aldehyde **10** was used while the yield of another four-ring acene **7d** was only 15% when the aldehyde **3d** was employed, both aldehydes derived from benzothiophene. The structure of **7d** was unambiguously confirmed via X-ray analysis (Figure 1).

Increasing the reaction temperature, HCl concentration or prolonging reaction time resulted in the formation of unexpected *HO(CH_2_)_2_S*-acenes **8** in yields up to 27%, which had never been observed in this type of reaction before.

**Scheme 3:**
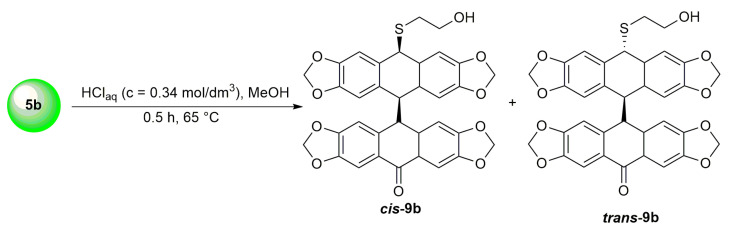
Formation of dimeric isomers *cis*-**9b** and *trans*-**9b** from *o*-(*O,S*-acetalaryl)arylmethyl methyl ether **5b**.

Surprisingly, neither the acene **7** nor the acene **8** was formed in this case. TLC and HPLC analysis confirmed the presence of the two products (Scheme 3). In both ^1^H and ^13^C NMR spectra, doubling signals were observed in a 2:1 ratio, which corresponded to the formation of the two isomers *cis*-**9b**/*tran*s-**9b**. In the ^13^C NMR spectrum, characteristic signals due to *cis*-**9b**/*tran*s-**9b** carbonyl groups were observed at 195.09 and 194.34 ppm. The presence of the latter was further confirmed by observation of the band at 1697 cm^−1^ in the IR spectrum and by the DFT calculation.

### 2.2. DFT Calculations for ***9a*** and ***9b***

**Figure 2:**
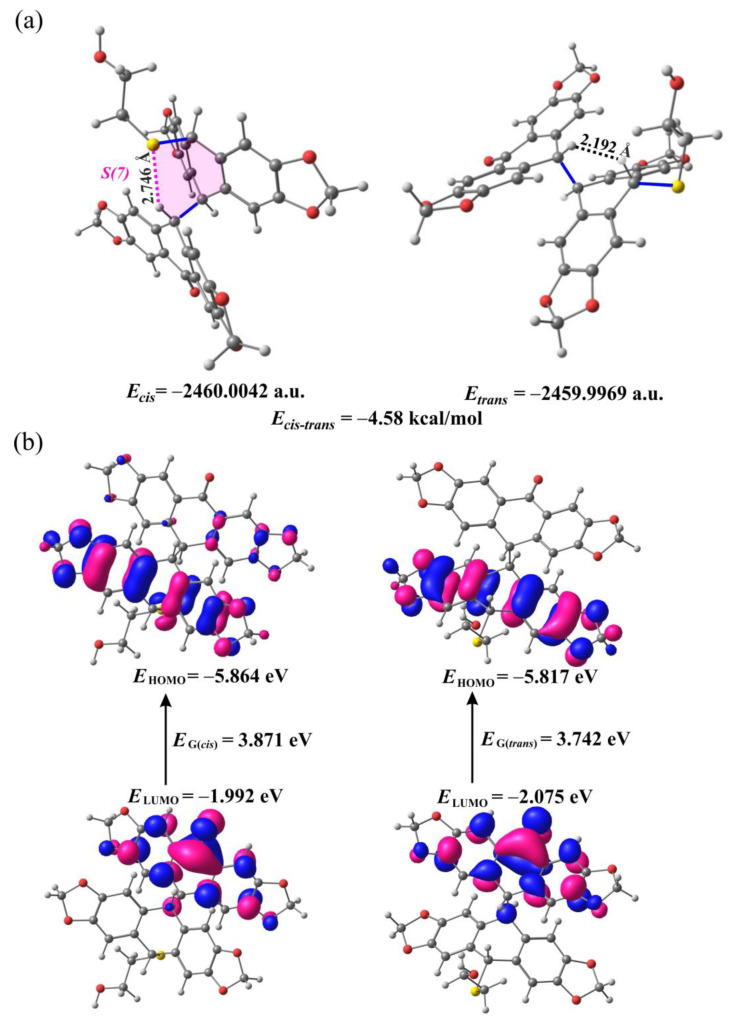
Molecular structures *cis*-**9b** and *trans*-**9b** (**a**) and their HOMO and LUMO orbitals (**b**) optimized at the DFT theory B3LYP/6-31++G(d,p). The dashed pink and black lines denote non-covalent C-H···S and H···H interactions, respectively. The blue solid lines show *cis* and *trans* configurations.

**Figure 3:**
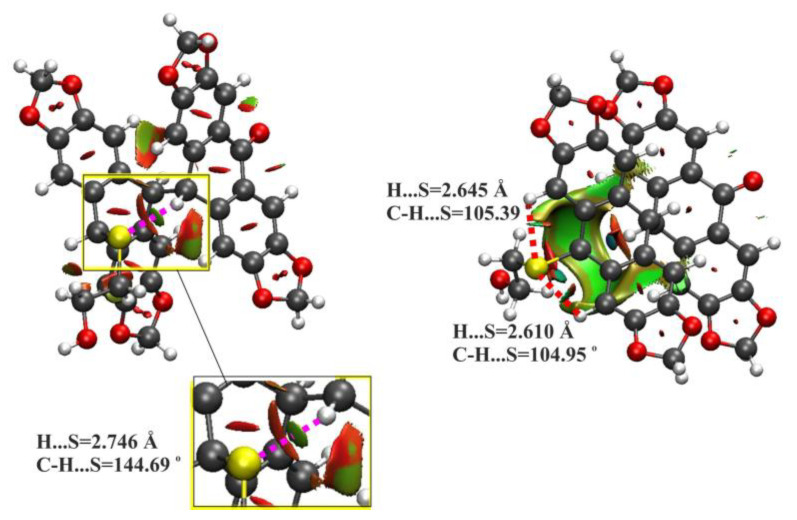
Color-filled reduced-density gradient (RDG) isosurfaces depicting non-covalent interactions in *cis*-**9b** (**left**) and *trans*-**9b** (**right**). The pink and red dashed lines denote weak C-H···S hydrogen bonds, respectively.

The optimized geometries and electronic structures of both the *cis*- and *trans*-isomers of **9b** in the gas phase, at the ground state obtained from DFT calculations using the gradient-corrected three-parameter hybrid functional (B3LYP) with the 6-31++G(d,p) basis set, are presented in Figure 2 (see also Tables S1 and S2 in Supplementary Materials). According to the DFT calculations, the *cis*-**9b** is more thermodynamically stable than the *trans***-9b** by 4.58 kcal/mol (Figure 2a). It is seen from Figure 2b that *cis*-**9b** is also chemically more stable with HOMO-LUMO energy gap (*E_G_*) of 3.871 eV compared to the *trans* isomer with *E_G_* = 3.742 eV. The higher thermodynamic and chemical stability of the *cis* isomer compared to the *trans* isomer may be due to the presence of an intramolecular non-covalent interaction between the S atom of the substituent attached to the cyclohexadiene ring of the *cis* isomer and the H atom of the cyclohexanone ring (dashed pink lines in Figure 2a and Figure 3), as revealed by the non-covalent interaction (NCI) analysis (see Supplementary Materials for details). The distance between non-covalently bonded S and H atoms (2.746 Å) is smaller by 0.25 Å than the sum of their van der Waals radii (3.00 Å). The geometrical parameters of this interaction, and especially the C-H···S angle of 144.69° (Figure 3), indicated that it could be treated as a weak unconventional hydrogen bond of the C-H···S type. This interaction forms a closed seven-membered ring *S*(7) (Figure 2a). The distance between the corresponding S and H atoms in *trans***-9b** is 4.534 Å, so this kind of non-covalent interaction has no possibility to occur in this molecule. It is worth noting that in the case of the *trans* isomer, the S atom is involved in the formation of short non-covalent contacts with the hydrogen atoms attached to aromatic carbons (Figure 3). Different non-covalent interactions involving the sulfur atom, i.e., C-H···S (in *cis***-9b**) and two C-H···S (in *trans***-9b**) interactions (Figure 2a and Figure 3), led to differences in the molecular configuration of the two isomers. For example, in *cis*-**9b**, the distance between the sulfur atom and the cyclohexan-1-one 4-C_sp3_ atom is only 3.692 Å, while in the *trans* isomer, it is about 1.5 Å greater. In the *cis* isomer, the C-H···S interaction forms an intramolecular ring *S*(7) that prevents free rotation around the C_sp3_-C_sp3_ bond connecting the two-ring systems.

### 2.3. Mechanistic Considerations and DFT Calculations

**Scheme 4:**
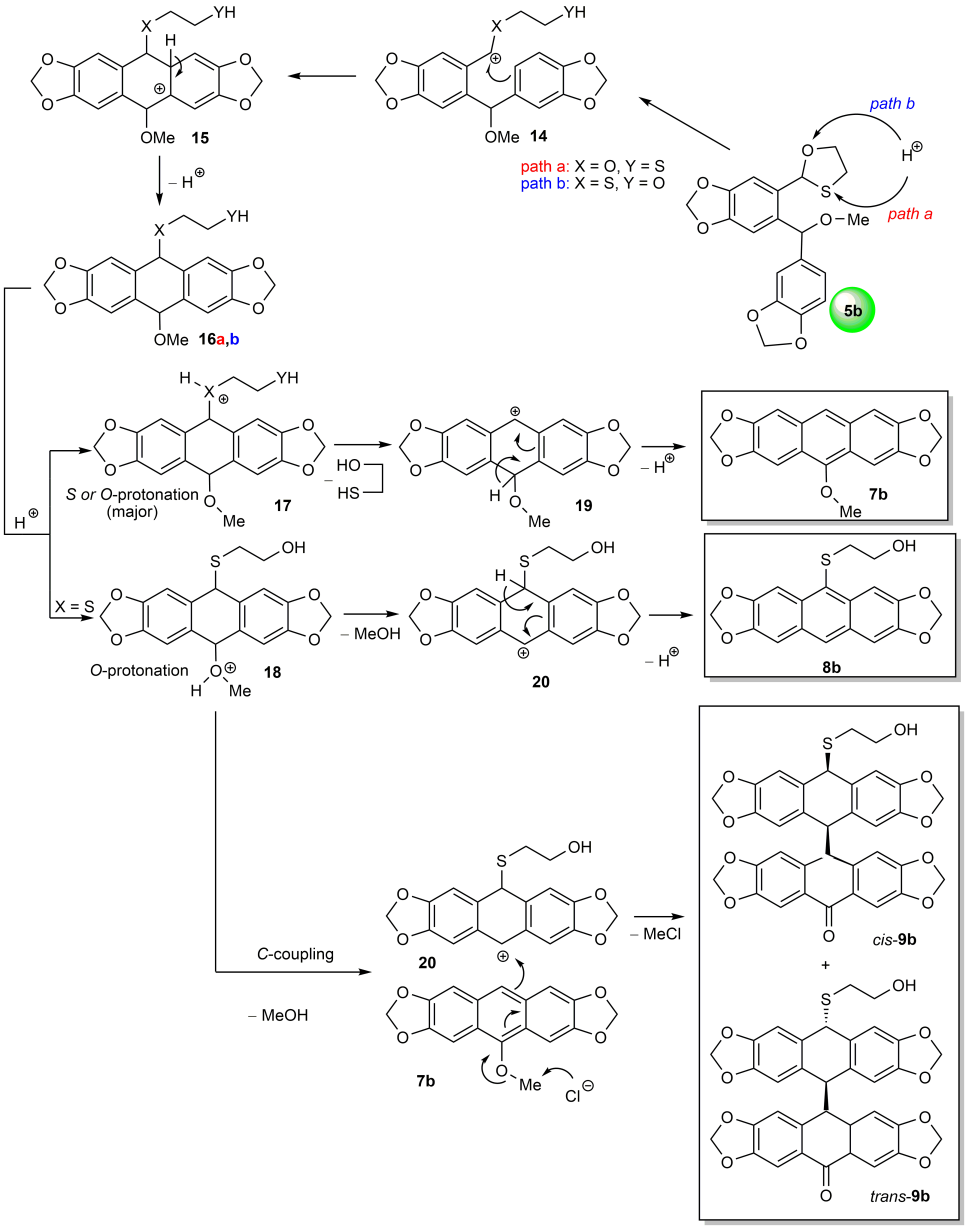
General mechanism for the *oxo*-Friedel–Crafts–Bradsher cyclization reaction using *O,S*-acetals on the example of the *O,S*-ether **5b** cyclization.

**Figure 4:**
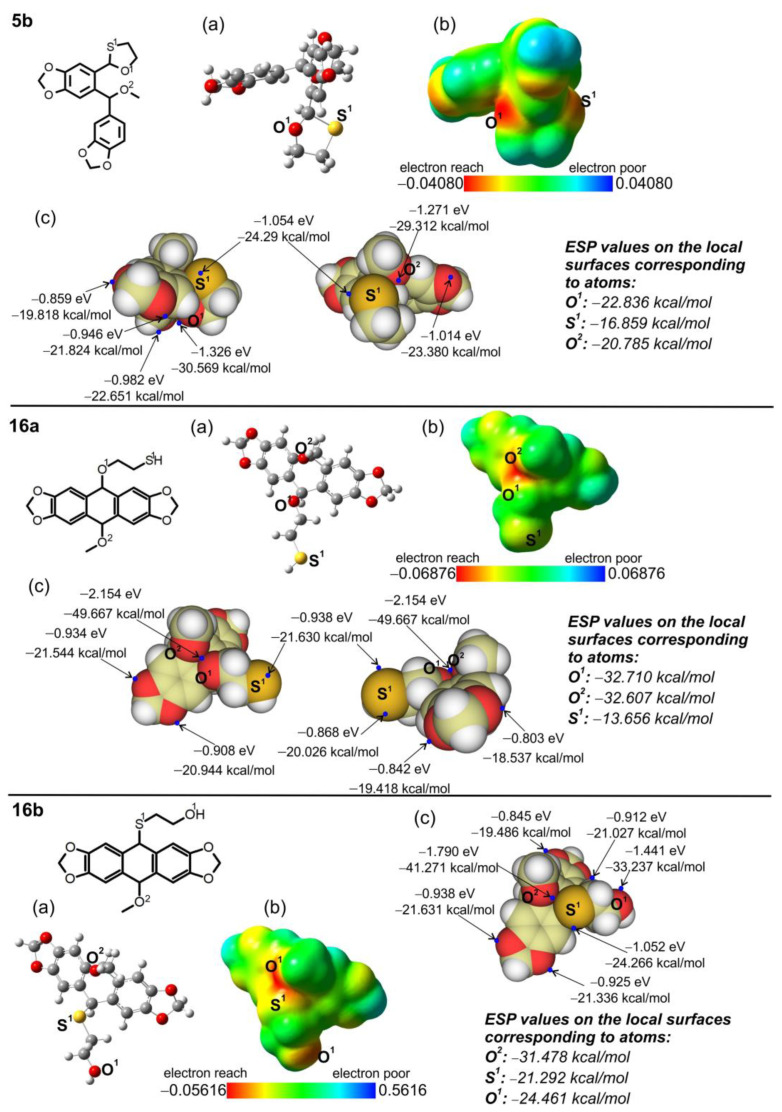
(**a**) Molecular structures of **5b**, **16a** and **16b** optimized at the DFT theory B3LYP/6-311++G(d,p) (see also Tables S3–S5 in Supplementary Materials), (**b**) electron density maps from total SCF B3LYP mapped with ESP (isovalue = 0.02; blue corresponds to low electron densities, whereas red corresponds to high electron densities), (**c**) the largest minima of ESP (blue points) on the van der Waals surfaces and the ESP values on the local surfaces corresponding to O^1^, O^2^ and O^3^ atoms (italic).

The formation of two unexpected types of products **8** and **9** made it possible to explain not only a pathway for obtaining these products but also to propose the overall mechanism of the *oxo*-Friedel–Crafts–Bradsher cyclization reaction using *O,S*-acetals (Scheme 4). To make the proposed mechanism credible, we performed DFT calculations (B3LYP 6-311++G(d,p)) in the gas phase in the ground state and the quantitative analysis of molecular surfaces [42,43] for **5b**, **16a** and **16b**. As a result of this analysis, the largest minima of electrostatic potential (ESP) on the van der Waals surfaces of the compounds and more precise ESP values on the local surfaces (surface corresponding to a given atom) were calculated for oxygen and sulfur atoms in the *O,S*-acetal **5b** as well as for the MeO oxygen and X atom (X = O, S) in the XCH_2_CH_2_YH side chains in **16a** and **16b** (Scheme 4, Figure 4). ESP values reflect the electron density in these atoms. As the electron density in a given atom decreases, its affinity for the proton also decreases, making the atom less basic, and vice versa. The atom is red, indicating that it is rich in electrons, and if the color of the atom gradually changes toward yellow and green, then the atom becomes steadily less rich in electrons.

Thus, on the basis of the obtained experimental and calculation data, we assumed that both oxygen and sulfur atoms can be protonated in strained five-membered *O,S*-acetals systems, with an obvious preference for the *O,S*-acetal oxygen atom because the difference in the ESP values between oxygen (−22.836 kcal/mol) and sulfur (−16.859 kcal/mol) atoms in **5b** is only −5.977 kcal/mol. It means that the difference in electron density in sulfur and oxygen in the cyclic *O,S*-acetal, and consequently affinity of the latter for the proton, may be regarded as comparable (*cf.* **16b**, vide infra). We also assumed that protonation of the sulfide sulfur atom in the strain-free side groups of intermediates **15**–**18** (X = S) and also in the final products **9b** would be more difficult than in the strained *O,S*-acetals because the difference in the ESP values between oxygen (−31.478 kcal/mol) and sulfur (−21.292 kcal/mol) atoms in **16b** is twice as high as in **5b** and equals 10.186 kcal/mol. However, the protonation in **16b** to give **17** (X = S) can be possible, to some extent, under harsh reaction conditions, SUCH AS higher HCl concentration, higher temperature and/or longer reaction times. This minor pathway, as in the case of major pathway for **16a,** also leads to carbocation **19** and then to anthracene **7b**.

Thus, the possible protonation of both heteroatoms leads to *O,S*-acetal cleavage and formation of the reactive benzylic carbocation **14** followed by the intramolecular S_E_Ar cyclization to give **15** and next aromatization one of the benzene rings to form **16a,b**. Preferential protonation of oxygen in **16a** or sulfur atoms in **16b** (the latter under harsh conditions), as mentioned above, produces **17,** which next undergoes aromatization through the intermediate dibenzylic carbocation **19** to give the major cyclization product **7.** Protonation of the MeO oxygen atom in **16b** gives **18** and, after aromatization through **19**, delivers the minor aromatic product **8b** of the *oxo*-Friedel–Crafts–Bradsher reaction. Finally, the obtained product **7b** couples with the intermediate dibenzylic carbocation **20** to give the dimeric products *cis*-**9b** and *tran*s-**9b.** This reaction predominates over pathways leading to **7b** and **8b** at higher temperature (65 °C) and higher HCl concentration (c = 0.34 mol/dm^3^).

### 2.4. Electron Character of RO-Acenes ***7***, ***13*** and HO(CH_2_)_2_S-Acenes ***8***

The electron nature of the obtained highly substituted acenes **7** and **8** and, in consequence, their photophysical properties are related to the character of the substituents attached to the acene system. The measure of the electron effect exerted by these substituents is the Hammett constants, which were calculated using the ACD/Percepta program [44]. The methoxy and methylene-1,3-dioxa groups with negative σ_p_ values of −0.27 and −0.13, respectively, have a strong electron donor character and increase electron density in acene systems **7a**–**d** and **13**. On the other hand, the small but positive values of σ_p_ = 0.07 constants confirm a weak electron-acceptor character of the *HO(CH_2_)_2_S*- group when attached to electron-rich acenes **8b** and **8c** substituted by electron-donating alkoxy groups [3,45].

The electron effects operating in the discussed functional groups (MeO, methylene-1,3-dioxa- and *HO(CH_2_)_2_S*-) were further analyzed with the σ_ind_ and σ_res_ Hammett’s components. They show that the electron-donating properties of the methoxy group with σ_ind_/σ_res_ = 0.30/−0.58 and methylene-1,3-dioxa group with σ_ind_/σ_res_ = 0.35/−0.48 are connected with a dominance of the positive resonance (+M) effect over the inductive (−I) effect of both alkoxy-type substituents.

On the other hand, in the *HO(CH_2_)_2_S*- group with σ_ind_/σ_res_ = 0.26/−0.21, the predominantly negative inductive effect (−I) dominates over the resonance effect. It accounts for the electron-withdrawing character of the RS group in electron-rich aromatics **8b** and **8c**.

### 2.5. Photopysical Properties

**Figure 5:**
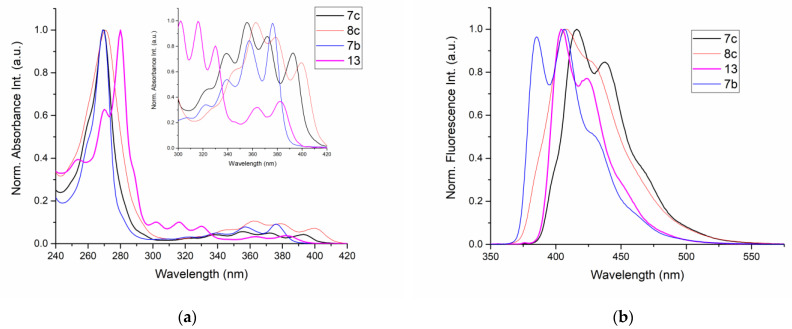
Normalized absorption (**a**) and emission (**b**) spectra of **7b**, **7c**, **8c** and **13** in CH_2_Cl_2_ (10^−5^ mol/dm^3^, 25 °C).

Thus, *RO*-acenes **7** and **13** belong to a group of highly substituted donor chromophores absorbing UV light in a typical range of 270–395 nm and emitting blue light at 380–445 nm [32]. Sulfur-substituted products, represented here by *HO(CH_2_)_2_S*-acenes **8b** and **8c,** belong to a group of donor-acceptor chromophores that normally absorb light in a range of 270–425 nm and emit blue light at longer wavelengths of 404–457 nm [3]. UV/Vis absorption and emission spectra of the obtained substituted acene derivatives **7b**, **7c**, **8c** and **13** are shown in Figure 5 and Table 2.

**Figure 6:**
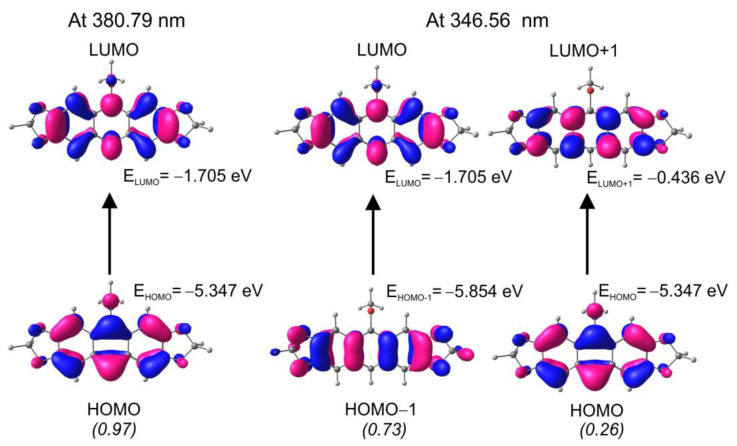
TD-DFT calculated orbitals involved in the electron transitions for **7b**.

In particular, in a range of 240–300 nm electron-donor anthracene **7b**, **7c** and a weak electron donor-acceptor anthracene **8c** due to the presence of the thio group with the −I effect, revealed almost identical absorption maxima at c.a. 269 nm and the same absorption profile. A further comparison of absorption spectra of electron-rich three-ring acenes (**7b**, **7c**), with the four-ring acene **13** of the same electron character, showed a redshift by **11** nm in a range of 240–300 nm, which indicated the effect of a larger aromatic conjugation in **13** (Figure 5a). In a range of 300–420 nm, all investigated compounds exhibited absorption bands of lower intensity in the long-wavelength part of the spectrum. The TD-DFT calculations in the gas phase, made for **7b**, revealed, in this part of the spectrum, two strong transitions, i.e., HOMO → LUMO (0.97), corresponding to a band at 380.79 nm and HOMO-1 → LUMO (0.73), HOMO → LUMO+1 (0.26) at 346.56 nm (Figure 6).

The fluorescence spectra of the obtained acenes exhibited blue emission and covered a region from 385 to 438 nm (Figure 5b). A redshift of 19–31 nm was observed for emission maxima of **7c**, **8c** and **13** relative to **7b.**

## 3. Materials and Methods

Organic solvents were purchased from commercial sources (ChemPur, Piekary Śląskie, Poland) and used as received or dried using standard procedures. Tetrahydrofuran (THF) was purchased from J.T. Baker and purified on Solvent Purification System (MBraun SPS-800). All reagents were from commercial suppliers (Sigma-Aldrich, Merck–USA, Beijing, China) and used without further purification. The ^1^H NMR and ^13^C NMR spectra were measured with a Bruker AV 200 or AV 500 spectrometer (Billerica, MA, USA), with chemical shifts given in ppm relative to TMS as an internal standard. High-resolution mass spectrometry (HRMS) measurements were performed using SQ Detector 2 mass spectrometer (Waters, Milford, MA, USA). Melting points were measured using Boetius apparatus. Thin-layer chromatography (TLC) was performed on precoated Merck 60 (F254 60, Darmstad, Germany) silica gel plates with fluorescent indicator, with detection by means of UV light at 254 and 360 nm. Column chromatography was performed on Merck silica gel (Kieselgel 60, Darmstad, Germany, 230–400 mesh) or using Pure FlashPrep 850 Chromatography System (Büchi, Flawil, Switzerland). The UV-Vis absorption spectra were recorded in 1 cm cuvettes on a Shimadzu UV-2700 spectrophotometer (Kioto, Japan) using two types of light source: a D2 64604 deuterium lamp and a Wl L6380 halogen lamp (Kioto, Japan, 220–600 nm). The emission spectra were obtained with the Horiba Jobin Yvon, FluoroMax-4 spectrofluorometer (Glasgow, UK), using a xenon lamp as a light source. The IR absorption spectra were recorded on Nicolet 6700 FT-IR spectrometer (Thermo Scientific, Waltham, MA, USA).

### 3.1. Synthesis of o-Bromopiperonal O,S-acetal ***2***

2-Mercaptoethanol (7.50 g, 96.05 mmol, 6.7 mL) and *p*-TsOH·H_2_O (10 mol%, 1.83 g) were added to a solution of o-bromopiperonal **1** (20 g, 87.32 mmol) in benzene (200 mL) and refluxed for 24 h using the Dean–Stark trap to remove water. The mixture was concentrated and purified with column chromatography using toluene as an eluent to give a colorless solid of **2** (20 g, 80%). mp = 72–73 °C. (Lit.: 73–74 °C [46])

^1^H NMR (500 MHz, CDCl_3_): 7.11 (s, 1H), 6.95 (s, 1H), 6.23 (s, 1H), 5.96 (d, *J* = 10.1 Hz, 1H), 5.95 (d, *J* = 10.1 Hz, 1H), 4.64–4.50 (m, 1H), 3.92 (dt, *J* = 9.0, 6.1 Hz, 1H), 3.28–3.08 (m, 2H) ppm. ^13^C{^1^H} NMR (126 MHz, CDCl_3_): 148.3, 147.7, 132.7, 112.5, 112.4, 107.3, 102.0, 85.8, 72.2, 33.7 ppm. Anal. calcd for C_10_H_9_BrO_3_S: C, 41.54, H, 3.14, S, 11.09; found C, 41.49, H, 3.17, S, 11.03. HRMS (ESI): *m*/*z* [M + H]^+^ calcd for C_10_H_10_BrO_3_S: 288.9534; found 288.9536.

### 3.2. General Procedure for the Synthesis of o-(O,S-acetalaryl)arylmethanols ***4***

*o*-Bromopiperonal O,S-acetal 2 (0.289 g, 1.0 mmol) was placed in the round-bottom flask (50 mL) and dissolved in dry THF (8 mL) under argon atmosphere. The temperature of the resulting solution was lowered to −78 °C and *n*-BuLi (1.1 mmol, 2.5 M in hexanes) was added. The resulting mixture was stirred under argon for 15 min and then the corresponding aromatic aldehyde 3a–d (1.2 mmol) in dry THF (5 mL) was added. Stirring was continued for 2 h at −78 °C and the reaction mixture was warmed to room temperature. The saturated aqueous NH_4_Cl solution was added and organic layer was concentrated. The residue was diluted with ethyl acetate (3 × 10 mL), washed with water (15 mL) and dried over anhydrous MgSO_4_. After filtration, ethyl acetate was removed in vacuum and the crude product 4 was purified by column chromatography over silica gel with a mixture of toluene/ethyl acetate (1:1 *v*/*v*) as an eluent.

*(6-(1,3)-Oxathiolan-2-yl-benzo[d][1,3]dioxol-5-yl)(3,4,5-trimethoxyphenyl)methanol **4a***. ^1^H NMR (200 MHz, C_6_D_6_): 7.44 (s, 1H), 7.13 (s, 1H), 6.84 (s, 2H), 6.34 (s, 1H), 6.24 (d, *J* = 2.5 Hz, 1H), 5.32 (dd, *J* = 5.9, 1.2 Hz, 2H), 3.98 (ddd, *J* = 9.3, 6.1, 2.2 Hz, 1H), 3.88 (s, 3H), 3.46 (s, 6H), 3.29 (ddd, *J* = 9.3, 6.1, 6.1 Hz, 1H), 2.87–2.67 (m, 2H), 2.58 (ddd, *J* = 9.3, 6.1, 2.2 Hz, 1H) ppm. ^13^C{^1^H} NMR (50 MHz, C_6_D_6_): 152.6, 147.0, 146.2, 137.6, 136.9, 135.4, 129.5, 126.8, 107.0, 106.5, 102.9, 102.0, 99.8, 83.3, 70.4, 70.1, 59.0, 54.2, 32.4. Anal. calcd for C_20_H_22_O_7_S: C, 59.10, H, 5.46, S, 7.89; found C, 59.11, H, 5.50, S, 7.82. HRMS (ESI): *m*/*z* [M + Na]^+^ calcd for C_20_H_22_O_7_SNa: 429.0984; found 429.0985. Yield: 68%, colorless oil.

*(6-(1,3)-Oxathiolan-2-yl-benzo[d][1,3]dioxol-5-yl)(benzo[d][1,3]dioxol-5-yl)methanol* **4b**. Two diastereoisomers (A and B, 2:1)—^1^H NMR (200 MHz, C_6_D_6_): 7.39 (s, 1H, B), 7.37 (s, 1H, A), 7.09 (d, *J* = 1.6 Hz, 1H, B), 7.06 (d, *J* = 1.6 Hz, 1H, A), 7.01 (s, 1H, A), 6.97 (s, 1H, B), 6.93–6.76 (m, 2H, A+B), 6.71–6.53 (m, 2H), 6.22 (s, 1H, B), 6.17 (s, 1H, A), 6.03 (s, 1H, A), 5.97 (s, 1H, B), 5.38–5.18 (m, 8H), 3.88 (ddd, *J* = 8.9, 6.4, 2.4 Hz, 2H, A+B), 3.20 (ddd, *J* = 9.3, 4.7, 4.7 Hz, 2H, A+B), 2.76–2.58 (m, 2H, A+B), 2.56–2.40 (m, 2H, A+B), 2.15 (s br, 2H, A+B) ppm. ^13^C{^1^H} NMR (50 MHz, C_6_D_6_): 146.8, 146.8, 146.7, 146.7, 146.1, 146.1, 145.7, 136.5, 136.3, 134.9, 129.4, 129.3, 126.8, 119.0, 118.9, 106.7, 106.6, 106.4, 106.3, 106.1, 99.9, 99.6, 82.90, 82.5, 70.2, 70.1, 69.8, 63.2, 32.4, 32.4 ppm. Anal. calcd for C_18_H_16_O_6_S: C, 59.99, H, 4.48, S, 8.90; found C, 59.95, H, 4.50, S, 8.86. HRMS (ESI): *m*/*z* [M + Na]^+^ calcd for C_18_H_16_O_6_SNa: 383.0566; found 383.0565. Yield: 32%, yellowish oil.

*6-(1,3-Oxatiolan-2-yl)benzo[d](1,3-dioxol-5-yl)(benzo[b]thien-2-yl)methanol* **4d**. Two diastereoisomers (A and B, 1:1)—^1^H NMR (200 MHz, C_6_D_6_): 7.67–7.46 (m, 4H, A+B), 7.43 (s, 1H, A), 7.41 (s, 1H, B), 7.20–6.94 (m, 6H, A+B), 6.40 (s, 1H, A), 6.35 (s, 1H, B), 6.31 (s, 2H, A+B), 5.33 (dd, *J* = 14.5, 5.1 Hz, 4H, A+B), 4.08–3.84 (m, 2H, A+B), 3.50–3.30 (m, 1H, A), 3.27 (dt, *J* = 15.3, 8.9 Hz, 1H, B), 2.70 (td, *J* = 16.2, 9.4 Hz, 2H, A+B), 2.60–2.43 (m, 2H, A+B) ppm. ^13^C{^1^H} NMR (50 MHz, C_6_D_6_): 147.7, 146.9, 146.6, 138.9, 138.6, 133.8, 129.4, 122.9, 122.4, 121.2, 120.2, 106.6, 106.2, 100.0, 82.9, 70.1, 68.0, 32.5 ppm. Anal. calcd for C_19_H_16_O_4_S_2_: C, 61.27, H, 4.33, S, 17.22; found C, 61.25, H, 4.36, S, 17.22. HRMS (ESI): *m*/*z* [M + Na]^+^ calcd for C_19_H_16_O_4_S_2_Na: 395.0388; found 395.0387. Yield: 88%, yellowish oil.

### 3.3. General Procedure for the One-Pot Synthesis of o-(O,S-acetalaryl)arylmethyl Methyl Ethers 5 from o-Bromopiperonal O,S-acetal ***2*** (Method A)

*o*-Bromopiperonal *O,S*-acetal 2 (0.289 g, 1.0 mmol) was placed in the round-bottom flask (50 mL) and dissolved in dry THF (8 mL) at −78 °C under argon atmosphere. Next, *n*-BuLi (1.1 mmol, 2.5 M in hexanes) was added. The resulting mixture was stirred under argon for 15 min, and then the corresponding aromatic aldehyde 3a–c or 3d (1.2 mmol) was added in dry THF. Stirring was continued for 2 h at −78 °C and 5 equiv. of MeI was added. The reaction mixture was warmed to room temperature. The saturated aqueous NH_4_Cl solution was added, and organic layer was concentrated. The residue was diluted with ethyl acetate (3 × 10 mL), washed with water (15 mL) and dried over anhydrous MgSO_4_. After filtration, ethyl acetate was removed in vacuum and the crude product 5 was purified by column chromatography over silica gel with a mixture of toluene/ethyl acetate (1:1 *v*/*v*) as an eluent.

*5-[Methoxy-(3,4,5-trimethoxyphenyl)methyl]-6*-[1,3]*oxathiolan-2-ylbenzo[1,3]dioxole* **5a**. Two diastereoisomers (A and B, 1.4: 1)—^1^H NMR (200 MHz, C_6_D_6_): 7.62 (s, 1H, A), 7.56 (s, 1H, B), 7.11 (s, 1H, B), 7.09 (s, 1H, A), 6.90 (s, 2H, B), 6.82 (s, 2H, A), 6.52 (s, 1H, B), 6.45 (s, 1H, A), 5.59 (2×s, 2H, A+B), 5.36–5.24 (m, 4H, A+B), 4.13–3.94 (m, 2H, A+B), 3.90 (2×s, 6H, A+B), 3.51 (4×s, 18H, A+B), 3.46, 3.36, 3.32, 3.38–3.19 (m, 2H, A+B), 2.86–2.68 (m, 2H, A+B), 2.66–2.51 (m, 2H, A+B) ppm. ^13^C{^1^H} NMR (50 MHz, CDCl_3_): 152.0, 151.8, 146.7, 146.5, 146.3, 146.2, 135.7, 135.0, 131.3, 130.6, 130.1, 106.3, 106.1, 105.6, 105.5, 102.8, 102.5, 100.0, 82.0, 81.9, 79.9, 79.6, 70.7, 70.6, 59.5, 55.8, 54.8, 32.9, 32.7, 28.4 ppm. Anal. calcd for C_21_H_24_O_7_S: C, 59.99, H, 5.75, S, 7.62; found C, 59.91, H, 5.70, S, 7.66. HRMS (ESI): *m*/*z* [M + Na]^+^ calcd for C_21_H_24_O_7_SNa: 443.1140; found 443.1136. Yield: 83%, yellowish crystals, mp: 110–112 °C.

*5-((5-(1,3-Oxathiolan-2-yl)benzo[d][1,3]dioxol-6-yl)(methoxy)methyl)benzo[d][1,3]dioxole* **5b**. Two diastereoisomers (A and B, 1.6: 1)—^1^H NMR (500 MHz, C_6_D_6_): 7.46 (s, 1H, A), 7.41 (s, 1H, B), 7.04 (s, 1H, B), 7.00 (s, 1H, A), 6.97 (s, 1H, A), 6.91 (s, 1H, B), 6.78 (d, *J* = 8.00 Hz, 1H, B), 6.72 (d, *J* = 8.00 Hz, 1H, A), 6.58 (d, *J* = 8.00 Hz, 1H, B), 6.55 (d, *J* = 8.00 Hz, 1H, A), 6.30 (s, 1H, B), 6.23 (s, 1H, A), 5.35 (s, 1H, A), 5.28 (s, 1H, B), 5.25–5.14 (m, 8H, A+B), 3.93–3.83 (m, 2H, A+B), 3.23–3.14 (m, 2H, A+B), 3.15 (s, 3H, A), 3.11 (s, 3H, B), 2.68–2.56 (m, 2H, A+B), 2.50–2.36 (m, 2H, A+B) ppm. ^13^C{^1^H} NMR (126 MHz, C_6_D_6_): 149.1, 149.0 (2×s), 148.7 (2×s), 148.2, 137.0, 136.7, 134.2, 133.1, 133.0, 129.0, 121.7, 121.6, 109.0, 108.9, 108.8 (2×s), 108.7, 108.5, 108.0, 101.9, 101.7, 84.4, 84.3, 82.4, 82.2, 72.4, 72.4, 57.3, 34.6, 34.5 ppm. Anal. calcd for C_19_H_18_O_6_S: C, 60.95, H, 4.85, S, 8.56; found C, 60.98, H, 4.80, S, 8.50. HRMS (ESI): *m*/*z* [M + Na]^+^ calcd for C_19_H_18_O_6_SNa: 397.0722; found 397.0723. Yield: 65%, white crystals, mp: 116–117 °C.

*5-(Methoxy(3-methoxyphenyl)methyl)-6-(1,3-oxathiolan-2-yl)benzo[d][1,3]dioxole* **5c**. Two diastereoisomers A and B (5.5:1)—^1^H NMR (500 MHz, CDCl_3_): 7.57 (s, 1H, B), 7.52 (s, 1H, A), 7.30 (s, 1H, A), 7.26 (s, 1H, B), 7.15 (d, *J* = 4.55 Hz, 4H, A+B), 7.05 (s, 1H, B), 7.01 (s, 1H, A), 6.76 (s br, 2H, A+B), 6.45 (s, 1H, A), 6.38 (s, 1H, B), 5.58 (s, 1H, B), 5.52 (s, 1H, A), 5.28 (d, *J* = 18.56 Hz, 4H, A+B), 4.03–3.92 (m, 2H, A+B), 3.37 (s, 3H, A), 3.34 (s, 3H, B), 3.30 (s, 3H, B), 3.32–3.21 (m, 2H, A+B), 3.26 (s, 3H, A), 2.79–2.68 (m, 2H, A+B), 2.55–2.48 (m, 2H, A+B) ppm. ^13^C{^1^H} NMR (126 MHz, CDCl_3_): 160.1, 148.2, 147.9, 143.8, 143.4, 133.3, 132.3, 129.4, 129.4, 128.1, 119.5, 119.4, 113.1, 112.7, 112.6, 108.3, 107.8, 107.7, 107.2, 101.0, 83.5, 81.4, 71.5, 56.4, 54.5, 33.7 ppm. Anal. calcd for C_19_H_20_O_5_S: C, 63.32, H, 5.59, S, 8.90; found C, 63.29, H, 5.51, S, 8.94. HRMS (ESI): *m*/*z* [M + Na]^+^ calcd for C_19_H_20_O_5_SNa: 383.0929; found 383.0930. Yield: 67%, yellowish oil.

*5-((Benzo[b]thien-2-yl)(methoxy)methyl)-6-(1,3-oxathiolan-2-yl)benzo[d][1,3]dioxole* **5d**. Two diastereoisomers (A and B, 1.3:1 ratio). *Major diastereoisomer A*—^1^H NMR (200 MHz, C_6_D_6_): 7.65–7.43 (m, 3H), 7.29–6.98 (m, 4H), 6.38 (s, 1H), 5.83 (s, 1H), 5.42–5.30 (m, 2H), 3.99 (dd, *J* = 11.1, 4.4 Hz, 1H), 3.38–3.19 (m, 1H), 3.31 (s, 3H), 2.75 (ddd, *J* = 9.6, 6.6, 6.6 Hz, 1H), 2.54 (dd, *J* = 11.1, 4.4 Hz, 1H) ppm. Anal. Calcd for C_20_H_18_O_4_S_2_: C, 62.16, H, 4.69, S, 16.59; Found C, 62.13, H, 4.64, S, 16.55. HRMS (ESI): *m*/*z* [M + Na]^+^ calcd for C_20_H_18_O_4_S_2_Na: 409.0544; found 409.0545. Yield: 43%, yellowish oil. *Minor diastereoisomer B*—^1^H NMR (200 MHz, C_6_D_6_): 7.66–7.41 (m, 3H), 7.20–6.99 (m, 4H), 6.45 (s, 1H), 5.71 (d, *J* = 1.1 Hz, 1H), 5.32 (dd, *J* = 6.6, 1.3 Hz, 2H), 3.93 (ddd, *J* = 8.7, 6.4, 2.1 Hz, 1H), 3.33–3.11 (m, 1H), 3.27 (s, 3H), 2.71 (ddd, *J* = 9.7, 6.4, 6.4 Hz, 1H), 2.56–2.41 (m, 1H) ppm. ^13^C{^1^H} NMR (50 MHz, C_6_D_6_): 148.3, 147.4, 140.4, 139.7, 132.5, 132.2, 128.1, 124.1, 123.7, 122.4, 122.0, 108.3, 108.0, 101.2, 83.6, 79.0, 71.5, 56.5, 33.6 ppm. Anal. calcd for C_20_H_18_O_4_S_2_: C, 62.16, H, 4.69, S, 16.59; found C, 62.19, H, 4.51, S, 16.50. HRMS (ESI): *m*/*z* [M + Na]^+^ calcd for C_20_H_18_O_4_S_2_Na: 409.0544; found 409.0546. Yield: 33%, yellowish oil. Yield: 92% (diastereoisomers A+B).

### 3.4. Procedure for the Synthesis of o-(O,S-acetalaryl)arylmethyl Benzyl Ether ***6a***

*o*-Bromopiperonal *O,S*-acetal 2 (0.289 g, 1.0 mmol) was placed in the round-bottom flask (50 mL) and dissolved in dry THF (8 mL) at −78 °C under argon atmosphere. Next, *n-*BuLi (1.1 mmol, 2.5 M in hexanes) was added. The resulting mixture was stirred under argon for 15 min and then 3,4,5-trimethoxybenzaldehyde 3a (0.235 g, 1.2 mmol) was added in dry THF. Stirring was continued for 2 h at −78 °C then benzyl bromide (BnBr) (0.171 g, 1.0 mmol) was added. The reaction mixture was warmed to room temperature. The saturated aqueous NH_4_Cl solution was added, and organic layer was concentrated. The residue was diluted with ethyl acetate (3 × 10 mL), washed with water (15 mL) and dried over anhydrous MgSO_4_. After filtration, ethyl acetate was removed in vacuum and the crude product 6a was obtained as a yellow oil. The product decomposed on silica gel during purification attempts.

*5-((Benzyloxy)(3,4,5-trimethoxyphenyl)methyl)-6-(1,3-oxathiolan-2-yl)benzo[d][1,3]dioxole* **6a**. Two diastereoisomers (A:B, 2:1)—^1^H NMR (200 MHz, C_6_D_6_): 7.65 (s, 1H, B), 7.58 (s, 1H, A), 7.55–7.36 (m, 4H, A+B), 7.33–7.25 (m, 2H, A+B), 7.20–7.14 (m, 2H, A+B), 7.15–6.98 (m, 4H, A+B), 6.96 (s, 2H, A), 6.87 (s, 2H, B), 6.44 (s, 1H, B), 6.38 (s, 1H, A), 5.90 (s, 1H, B), 5.87 (s, 1H, A), 5.32 (d, *J* = 1.3 Hz, 2H, A), 5.27 (d, *J* = 1.3 Hz, 2H, B), 4.95–3.85 (m, 2H, A+B), 4.73 (d, *J* = 12.0 Hz, 1H, B), 4.58 (d, *J* = 12.0 Hz, 1H, B), 4.67 (d, *J* = 12.2 Hz, 1H, A), 4.52 (d, *J* = 12.2 Hz, 1H, A), 4.02 (s, 3H, B), 3.91 (s, 3H, A), 3.51 (s, 6H, A), 3.44 (s, 6H, B), 3.41–3.16 (m, 2H, A+B), 2.76 (dt, *J* = 9.6, 6.6 Hz, 2H, A+B), 2.54 (ddd, *J* = 9.6, 5.0, 2.0 Hz, 2H, A+B) ppm. Yield: 94% (crude product), yellow oil.

### 3.5. General Procedure for the Synthesis of o-(O,S-acetalaryl)arylmethyl Methyl Ethers ***5*** from o-(O,S-acetalaryl)arylmethanols ***4*** (Method B)

*o*-(*O,S*-Acetalaryl)arylmethanol **4a**,**b** or **4d** (1.0 mmol) and KI (5 mol%) were placed in the round-bottom flask (50 mL) and dissolved in dry THF (8 mL) at room temperature; then, *NaH* (1.1 mmol) was added and stirred for 30 min under argon atmosphere. Then, the resulting mixture was treated with MeI (1.5 mmol) and was left at room temperature overnight. After 12 h, the residue was diluted with ethyl acetate (3 × 10 mL), washed with water (15 mL) and dried over anhydrous MgSO_4_. After filtration, ethyl acetate was removed in vacuum and the crude product 5 was purified by column chromatography over silica gel with a mixture of toluene/ethyl acetate (1:1 *v*/*v*) as an eluent.

### 3.6. General Procedure for the Synthesis of MeO-Substituted Acenes ***7***, ***13*** and ***8*** Using HCl_aq_ (Method C)

To a solution of *o*-(*O*,*S*-acetalaryl)arylmethyl methyl ether **5** or **11** (0.8 mmol), dissolved in MeOH (20 mL), aqueous solution of 1*N* or 2*N* HCl (4 mL) was added and the resulting mixture was stirred at the relevant temperature (see Table 1) until disappearance of the starting material (monitoring by TLC). The reaction mixture was extracted with ethyl acetate (50 mL) and the organic layer was washed with water (30 mL), saturated solution of NaHCO_3_ (30 mL) and again with water (30 mL). After drying over anhydrous MgSO_4_ and filtration, the solvent was removed in vacuum and the crude products were purified by column chromatography over silica gel with a mixture of *n*-hexane/ethyl acetate 10:1 (*v*/*v*) to afford corresponding acenes **7** and **13**. A mixture of *n*-hexane/ethyl acetate 3:1 (*v*/*v*) was used to purify anthracene **8**.

### 3.7. General Procedure for the Synthesis of MeO-Substituted Acene ***7*** Using FeCl_3_/KI in MeOH (Method D)

To a solution of *o*-(*O*,*S*-acetalaryl)arylmethyl methyl ether **5** (1.1 mmol) in dry MeOH (10 mL), FeCl_3_ (0.195 g, 1.2 mmol) and KI (0.199 g, 1.2 mmol) were added. The mixture was refluxed until disappearance of the starting material (monitoring by TLC, Table 1). After completion of the reaction, the solvent was removed. To the crude product, ethyl acetate (10 mL) was added and the resulting mixture was poured onto the saturated solution of Na_2_S_2_O_3_ (10 mL). The organic layer was dried over anhydrous MgSO_4_. After filtration, the solvent was evaporated and the crude products were purified with column chromatography (*n*-hexane/ethyl acetate, 10:1 *v*/*v*) to give *MeO*-acenes **7** in up to 72**%** yield accompanying by *HO(CH_2_)_2_S*-acenes **8** in up to 27% yields (Table 1).

*5,7,8,9-Tetramethoxyanthra[2,3-d][1,3]dioxole* **7a** [34]. ^1^H NMR (200 MHz, C_6_D_6_): 8.52 (s, 1H), 7.74 (s, 1H), 7.37 (s, 1H), 7.20 (s, 1H), 5.35 (s, 2H), 4.01 (s, 3H), 3.93 (s, 3H), 3.81 (s, 3H), 3.56 (s, 3H) ppm. ^13^C{^1^H} NMR (50 MHz, CDCl_3_): 152.6, 148.0, 147.4, 140.6, 138.5, 128.9, 128.7, 124.0, 122.1, 121.3, 114.9, 103.3, 101.0, 95.8, 95.0, 61.8, 61.4, 55.9 ppm.

HRMS (ESI): *m*/*z* [M + Na]^+^ calcd for C_19_H_18_O_6_Na: 365.1001; found 365.0998. Yield: 53% (Method C), yellow crystals, mp: 130–132 °C.

*5-methoxyanthra[2,3-d:6,7-d’]bis[1,3]dioxole* **7b**. ^1^H NMR (500 MHz, CDCl_3_): 7.77 (s, 1H), 7.42 (s, 2H), 7.13 (s, 2H), 6.04 (s, 4H), 4.02 (s, 3H) ppm. ^13^C{^1^H} NMR (126 MHz, CDCl_3_): 150.5, 147.6, 147.5, 129.5, 121.3, 119.8, 106.4, 102.6, 101.0, 97.1, 62.1 ppm. HRMS (ESI): *m*/*z* [M + Na]^+^ calcd for C_17_H_12_O_5_Na: 319.0582; found 319.0584. Yield: 36% (Method D), yellow crystals, mp: 129–130 °C.

*5,7-Dimethoxyanthra[2,3-d][1,3]dioxole* **7c** [34]. ^1^H NMR (200 MHz, C_6_D_6_): 7.74 (s, 1H), 7.69 (d, *J* = 9.3 Hz, 1H), 7.54 (d, *J* = 2.5 Hz, 1H), 7.28 (dd, *J* = 9.3, 2.5 Hz, 1H), 7.23 (s, 1H), 7.14 (s, 1H), 5.35 (s, 2H), 3.79 (s, 3H), 3.55 (s, 3H) ppm. ^13^C{^1^H} NMR (126 MHz, C_6_D_6_): 158.3, 151.2, 149.4, 148.5, 130.9, 129.9, 129.5, 126.0, 123.9, 122.2, 120.8, 104.1, 101,7, 99.1, 98.1, 62.0, 55.4 ppm.

HRMS (ESI): *m*/*z* [M + Na]^+^ calcd for C_17_H_14_O_4_Na: 305.0790; found 305.0796. Yield: 78% (Method D), yellowish oil.

*5-Methoxy-6H-[1,3]benzodioxolo[3,2-b]dibenzothiophene*  **7d**. ^1^H NMR (500 MHz, C_6_D_6_): 7.90 (s, 1H), 7.83 (d, *J* = 7.8 Hz, 1H), 7.59 (s, 1H), 7.43 (d, *J* = 7.9 Hz, 1H), 7.17–7.12 (m, 1H), 7.10–7.06 (m, 2H), 5.25 (s, 2H), 3.70 (s, 3H) ppm. ^13^C{^1^H} NMR (126 MHz, C_6_D_6_): 149.0, 148.45, 146.1, 140.6, 135.9, 131.0, 128.7, 127.8, 124.9, 123.5, 122.5, 116.7, 115.9, 111.3, 104.7, 101.5, 98.2, 60.3 ppm. Anal. Calcd for C_18_H_12_O_3_S: C, 70.11, H, 3.92, S, 10.40; Found C, 70.09, H, 3.96, S, 10.44. HRMS (ESI): *m*/*z* [M + Na]^+^ calcd for C_18_H_12_O_3_SNa: 331.0405; found 331.0403. Yield: 62% (Method D) yellowish oil.

*2-(Anthra[2,3-d:6,7-d’]bis[1,3]dioxol-10-ylthio)ethanol* **8b**. ^1^H NMR (500 MHz, DMSO-*d_6_*): 8.10 (s, 1H), 7.99 (s, 2H), 7.25 (s, 2H), 6.12 (s, 4H), 4.77 (br s, 1H), 3.33 (m, 2H), 2.79 (t, *J* = 6.8 Hz, 2H) ppm. ^13^C{^1^H} NMR (126 MHz, DMSO-*d_6_*): 149.1, 147.4, 132.0, 128.62, 126.6, 125.1, 103.1, 101.9, 101.6, 60.5, 39.1. HRMS (ESI): *m*/*z* [M + H]^+^ calcd for C_18_H_15_O_5_S: 343.0640; found 343.0636. Yield = 25% (Method C), yellowish oil.

*2-(7-Methoxyanthra[2,3-d][1,3]dioxol-10-ylthio)ethanol* **8c**. ^1^H NMR (500 MHz, C_6_D_6_): 8.45 (s, 1H), 8.25 (d, *J* = 2.7 Hz, 1H), 7.78 (s, 1H), 7.52 (d, *J* = 9.0 Hz, 1H), 7.18 (dd, *J* = 9.0, 2.7 Hz, 1H), 6.98 (s, 1H), 5.22 (s, 2H), 3.55 (s, 3H), 3.16 (t, *J* = 6.3 Hz, 2H), 2.61 (t, *J* = 6.3 Hz, 2H) ppm. ^13^C{^1^H} NMR (126 MHz, C_6_D_6_): 158.5, 149.8, 147.2, 135.7, 134.1, 130.3, 128.2, 127.9, 124.4, 120.8, 119.7, 103.3, 103.2, 102.0, 100.8, 61.0, 54.6, 38.9 ppm. ^1^H NMR (500 MHz, DMSO-*d_6_*): 8.30 (s, 1H), 8.06 (s, 1H), 8.03 (s, 1H), 7.88 (d, *J* = 9.0 Hz, 1H), 7.35 (s, 1H), 7.13 (d, *J* = 9.0 Hz, 1H), 6.15 (s, 2H), 4.78 (br s, 1H), 3.92 (s, 3H), 3.43–3.25 (m, 2H), 2.84 (t, *J* = 6.7, Hz, 2H) ppm. ^13^C{^1^H} NMR (126 MHz, DMSO-*d_6_*): 158.1, 149.9, 147.3, 135.0, 133.8, 130.8, 128.0, 127.9, 127.2, 124.3, 119.6, 103.5, 103.5, 102.0, 101.4, 60.6, 55.6, 38.8 ppm. HRMS (ESI): *m*/*z* [M + H]^+^ calcd for C_18_H_17_O_4_S: 329.0848; found 329.0845. Yield: 27% (Method C), yellow solid, mp: 153–154 °C.

### 3.8. Synthesis of 3-Bromobenzo[b]thiophene-2-carbaldehyde O,S-acetal ***11***

2-Mercaptoethanol (2.62 mmol, 205 mg, 184 µL) and *p*-TsOH·H_2_O (50 mg, 0.2 mmol, 10 mol%) were added to a solution of 3-bromobenzo[b]thiophene-2-carbaldehyde **10** (2.62 mmol, 0.633 g) in benzene (6 mL), and the resulting mixture was refluxed for 24 h using the Dean–Stark trap to remove water. The mixture was concentrated and purified with column chromatography using toluene as an eluent. Evaporation of the solvent gave a colorless oil of **11** (0.580 g, 74%) [47].

^1^H NMR (200 MHz, C_6_D_6_): 7.67 (d, *J* = 7.6 Hz, 1H), 7.41 (d, *J* = 7.8 Hz, 1H), 7.20–7.10 (m, 1H), 7.10–6.99 (m, 1H), 6.55 (s, 1H), 4.10–3.95 (m, 1H), 3.55–3.33 (m, 1H), 2.92–2.72 (m, 1H), 2.57 (ddd, *J* = 12.6, 8.0, 5.2 Hz, 1H) ppm. ^13^C{^1^H} NMR (50 MHz, C_6_D_6_): 140.6, 138.2, 137.7, 128.7, 125.6, 125.0, 122.9, 122.6, 82.2, 72.1, 33.7 ppm. Anal. calcd for C_11_H_9_BrOS_2_: C, 43.86, H, 3.01, S, 21.29; found C, 43.83, H, 2.98, S, 21.35. HRMS (ESI): *m*/*z* [M + H]^+^ calcd for C_11_H_10_BrOS_2_: 300.9356; found 300.9347.

### 3.9. Synthesis of o-(O,S-acetalaryl)arylmethyl Methyl Ether ***12***

*o*-Bromothioacetal 11 (500 mg, 1.66 mmol) was placed in the round-bottom flask (50 mL) and dissolved in dry THF (6 mL) at −78 °C under argon atmosphere. Next, *n*-BuLi (0.7 mL, 2.6 M in hexanes, 1.83 mmol) was added. The resulting mixture was stirred for 15 min under argon and then solution of the aldehyde 3a (326 mg, 1.66 mmol) in dry THF (4 mL) was added. After 2 h, MeI (1.13 g, 8.0 mmol) was added. The reaction mixture was warmed to room temperature and stirred for 12 h. The saturated aqueous NH_4_Cl solution was added, and organic layer was concentrated. The residue was diluted with ethyl acetate (3 × 10 mL), washed with water (15 mL) and dried over anhydrous MgSO_4_. After filtration, ethyl acetate was removed in vacuum and the crude product was purified by column chromatography over silica gel with a mixture of toluene/ethyl acetate (10:1 *v*/*v*). Fraction with R*_f_* = 0.65 yielded 617 mg of product **12**.

*2-(3-(Methoxy(3,4,5-trimethoxyphenyl)methyl)benzo[b]thien-2-yl)-1,3-oxathiolane* **12**. Two diastereoisomers (A and B)—^1^H NMR (500 MHz, CDCl_3_): 8.04 (d, *J* = 8.2 Hz, 1H, A), 8.02 (d, *J* = 8.2 Hz, 1H, B), 7.58 (d, *J* = 8.2 Hz, 2H, A+B), 7.24–7.03 (m, 10H, A+B), 6.96 (s, 1H, A), 6.95 (s, 1H, B), 6.83 (s, 1H, A), 6.82 (s, 1H, B), 5.87 (s, 1H, A), 5.82 (s, 1H, B), 4.10 (dddd, *J* = 15.6, 9.2, 6.2, 2.9 Hz, 2H, A+B), 3.82 (s, 1H, A), 3.81 (s, 1H, B),3.55–3.39 (m, 2H, A+B), 3.49 (s, 6H, A), 3.48 (s, 6H, B), 3.29 (s, 6H, A+B), 3.00–2.84 (m, 2H, A+B), 2.66–2.59 (m, 2H, A+B) ppm. ^13^C{^1^H} NMR (126 MHz, CDCl_3_): 153.8, 143.4, 143.2, 139.7 (2×s), 138.8, 138.7, 138.3, 137.7, 136.3, 136.2, 132.9, 132.5, 129.1, 128.3, 125.4, 125.0, 124.2, 124.1, 123.9, 122.6, 122.5, 104.3, 81.4, 81.3, 79.2, 79.1, 72.2, 71.9, 60.2, 56.6, 55.6, 34.0, 21.2 ppm. Anal. calcd for C_22_H_24_O_5_S_2_: C, 61.09; H, 5.59; S, 14.82; found C, 61.06, H, 5.52, S, 14.77. HRMS (ESI): *m*/*z* [M + Na]^+^ calcd for C_22_H_24_O_5_S_2_Na: 455.0963; found 455.0965. Yield: 86%, yellowish oil.

### 3.10. Synthesis of Acene ***13*** Using HCl_aq_

To a solution of *o*-(*O*,*S*-acetalaryl)arylmethyl methyl ether **12** (0.073 g, 0.17 mmol), dissolved in MeOH (4 mL), the aqueous solution of 2 N HCl (0.8 mL) was added and the resulting mixture was stirred at room temperature for 12 h. The reaction mixture was extracted with ethyl acetate (20 mL) and the organic layer was washed with water (10 mL), saturated solution of NaHCO_3_ (15 mL) and again with water (10 mL), then dried over anhydrous MgSO_4_. After filtration, ethyl acetate was removed in vacuum and the crude product was purified with PLC plate with a mixture of hexane/acetone (3:1 *v*/*v*). Fraction with R*_f_* = 0.45 yielded 9 mg of product **13**.

*7,8,9,11-Tetramethoxybenzo[b]naphtho[2,3-d]tiophene* ***13***. ^1^H NMR (500 MHz, C_6_D_6_): 8.71 (d, *J* = 7.7 Hz, 1H), 8.47 (s, 1H), 7.49 (d, *J* = 7.7 Hz, 1H), 7.34 (s, 1H), 7.28 (dd, *J* = 7.7, 7,7 Hz, 1H), 7.15 (dd, *J* = 7.7, 7.7 Hz, 1H), 3.79 (s, 6H), 3.66 (s, 3H), 3.44 (s, 3H) ppm. ^13^C{^1^H} NMR (126 MHz, CDCl_3_): 153.2, 152.4, 147.7, 142.0, 140.0, 136.7, 134.5, 127.0, 126.4, 126.2, 125.8, 124.7, 123.0, 122.5, 111.4, 96.6, 60.9, 60.7, 60.1, 55.1 ppm. Anal. calcd for C_20_H_18_O_4_S: C, 67.78; H, 5.12; S, 9.05; found C, 67.75, H, 5.15, S, 9.09. HRMS (ESI): *m*/*z* [M + Na]^+^ calcd for C_20_H_18_O_4_SNa: 377.0823; found 377.0821. Yield: 15%; yellowish crystals.

*Crystal structure data:* C_20_H_18_O_4_S, M = 340.40, monoclinic, space group P2_1_/n (No. 14), a = 12.1338(3) Å, b = 7.37769(18) Å, c = 19.7054(6), β = 103.602(3)°, V = 1714.54(8) Å^3^, Z = 4, T = 293(2) K, D_calc_ = 1.373 g∙cm^−3^, CuKα radiation, 2θ_max_ = 134.912°, 15,804 reflections collected, 3063 reflections unique and 2763 reflections with *I* > 2σ(*I*). Final GooF = 1.042, R1 = 0.0388 and wR2 = 0.1095 for 2763 reflections and 231 parameters [48].

### 3.11. Synthesis of Dimeric Isomers cis-***9b*** and trans-***9b*** from o-(O,S-acetalaryl)arylmethyl Methyl Ether ***5b*** Using HCl_aq_

To a solution of *o*-(*O*,*S*-acetalaryl)arylmethyl methyl ether **5b** (0.3 g, 0.8 mmol), dissolved in MeOH (20 mL), aqueous solution of 2 N HCl (4 mL) was added and the resulting mixture was stirred for 0.5 h at 65 °C until disappearance of the starting material (monitoring by TLC). The reaction mixture was extracted with ethyl acetate (50 mL) and the organic layer was washed with water (30 mL), saturated solution of NaHCO_3_ (30 mL) and again with water (30 mL). After drying over anhydrous MgSO_4_ and filtration, the solvent was removed in vacuum and the crude products were purified by column chromatography over silica gel with a mixture of *n*-hexane/ethyl acetate in a 2:1 (*v*/*v*) ratio to afford isomeric *cis*-**9b** *and trans*-**9b** (2:1) as a deep-red solid in 55% yield (0.274 g).

^1^H NMR (500 MHz, C_6_D_6_, A+B): 7.60 (s, 1H, A), 7.55 (d, *J* = 1.7 Hz, 1H, B), 7.30 (dd, *J* = 8.0, 1.8 Hz, 1H, B), 7.22 (d, *J* = 1.7 Hz, 1H, B), 7.14 (s, 1H, B), 7.12 (s, 1H, B), 7.11 (s, 1H, A), 7.04 (dd, *J* = 8.1, 1.7 Hz, 1H, B), 6.73 (s, 1H, B), 6.67 (d, *J* = 1.8 Hz, 1H, A), 6.63 (d, *J* = 8.0 Hz, 1H, A), 6.64 (d, *J* = 8.0 Hz, 1H, B), 6.58 (s, 1H, A), 6.53 (d, *J* = 8.1 Hz, 1H, B), 6.48 (s, 1H, A), 6.44 (d, *J* = 2.1 Hz, 1H, A), 6.42 (s, 1H, A), 6.40 (d, *J* = 1.7 Hz, 1H, B), 6.31 (d, *J* = 6.0 Hz, 1H, A), 6.32 (d, *J* = 3.7 Hz, 1H, A), 5.53 (s, 1H, B), 5.44 (s, 1H, A), 5.29–5.08 (m, 16H, A+B), 3.61 (td, *J* = 6.3, 2.5 Hz, 2H, A), 3.24 (dtd, *J* = 16.9, 11.2, 5.7 Hz, 2H, B), 2.67–2.58 (m, 1H, A), 2.51–2.42 (m, 1H, A), 2.22 (dt, *J* = 13.7, 5.5 Hz, 1H, B), 2.15–2.06 (m, 1H, B) ppm. ^13^C{^1^H} NMR (126 MHz, C_6_D_6_, A+B): 195.1, 194.3, 155.5, 155.2, 151.5, 151.4, 149.0, 148.7, 148.4, 148.2 (2×s) 148.0, 147.9, 147.8 (2×s) 147.0, 146.9, 146.8, 146.5, 140.9 (2×s) 135.8, 134.7, 134.6 (2×s), 132.4, 132.0, 126.2, 125.8, 125.4, 125.3, 125.2, 124.8, 123.0, 122.7, 122.2, 122.0, 111.0, 110.8, 109.4, 109.1, 109.0, 108.9, 108.6, 108.5, 108.2, 108.0, 107.7, 105.2, 105.0, 104.3, 104.2, 101.8, 101.1 (2×s), 101.0, 100.7 (2×s), 61.2, 60.4, 50.4, 50.3, 35.6, 35.1 ppm. HRMS (ESI): *m*/*z* [M + Na]^+^ calcd for C_34_H_24_O_10_SNa: 647.0988; found 647.0989; *m*/*z* [M + K]^+^ calcd for C_34_H_24_O_10_SK: 663.0727; found 663.0732. FT-IR (v_max_/cm^−1^, neat): 926, 1005, 1239, 1440, 1471, 1500, 1696, 2895. Deep-red solid, mp: 118–119 °C.

## 4. Conclusions

In summary, in this study, we presented a new modification of the *oxo*-Friedel–Crafts–Bradsher (F-C-B) cyclization reaction using *O,S*-acetals, which have not been previously studied in this type of reaction. Unlike other *hetero*-F-C-B modifications, involving *O,O*-acetals and *S,S*-dithioacetals, this reaction is more flexible and can be carried out both in aqueous solutions and under anhydrous conditions, which is important for water-sensitive substrates. Like other *hetero*-F-C-B cyclizations, *oxo*-F-C-B cyclization may be a source of highly substituted acenes, the hallmark of this type of reaction. Interestingly, in this modification, two kinds of electron donor and donor-acceptor acenes can be obtained in one reaction step. The formation of two types of products **8** and **9**, which have never been observed in this type of cyclization, and the support of DFT calculations made it possible to propose a general reaction mechanism for this new modification of *oxo*-Friedel–Crafts–Bradsher cyclization.

## Figures and Tables

**Table 1:** A comparison of the cyclization results of ethers **5** and **12**.

Substrate	Reaction Conditions	*RO*-Acene (Yield) ^1^	*HO(CH_2_)_2_S*-Acene (Yield) ^1^
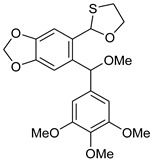 **5a**	HCl (0.17 mol/dm^3^, 10 equiv.), MeOH, 72 h, rt	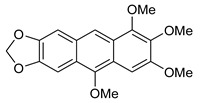 **7a** (53%)	not observed
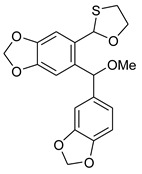 **5b**	HCl (0.17 mol/dm^3^, 10 equiv.), MeOH, 72 h, rt	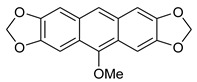 **7b** (37%)	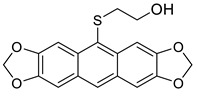 **8b** (12%)
	HCl (0.17 mol/dm^3^, 10 equiv.), MeOH, 72 h, rt then 8 h, 65 °C	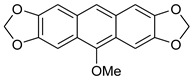 **7b** (8%)	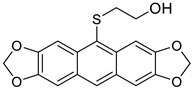 **8b** (25%)
	HCl (0.34 mol/dm^3^, 10 equiv.), MeOH, 0.5 h, 65 °C	not observed	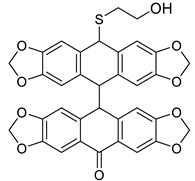 **9b** (55%)
	FeCl_3_/KI, MeOH, 3 h, 65 °C	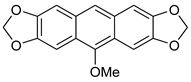 **7b** (36%)	not observed
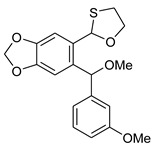 **5c**	HCl (0.17 mol/dm^3^, 10 equiv.), 2 h, 65 °C then 12 h, rt	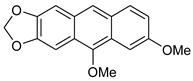 **7c** (35%)	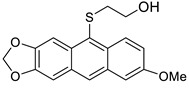 **8c** (9%)
	HCl, (0.34 mol/dm^3^, 5 equiv.), 4 d, rt	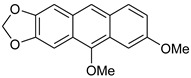 **7c** (25%)	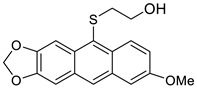 **8c** (27%)
	FeCl_3_/KI, MeOH, 12 h, 65 °C	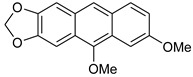 **7c** (78%)	not observed
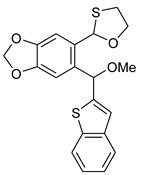 **5d**	HCl (0.17 mol/dm^3^, 10 equiv.), MeOH, 72 h, rt	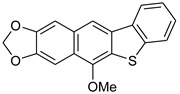 **7d** (62%)	not observed
	HCl (0.17 mol/dm^3^, 10 equiv.), MeOH, 8 h, 65 °C	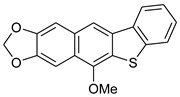 **7d** (33%)	not observed
	HCl (1.78 mol/dm^3^, 100 equiv.), 3 h, rt	not observed	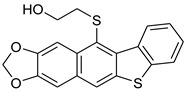 **8d** (14%) ^2^
	FeCl_3_/KI, MeOH, 12 h, 65 °C	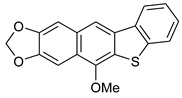 **7d** (62%)	not observed
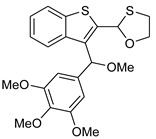 **12**	HCl (0.34 mol/dm^3^, 10 equiv.), MeOH, 12 h, rt	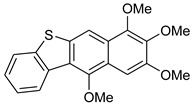 **13** (15%)	not observed

^1^ isolated yields; ^2^ yield based on ^1^H NMR spectrum (not isolated).

**Table 2:** Absorption and emission maxima (λ_max_), Stokes shifts in dichloromethane solution (10^−5^ mol/dm^3^, 25 °C) for **7b,c**, **8c** and **13** (underlined values are highest absorption maxima in the lower part of the spectrum in the 300–420 nm range).

Compound	Absorption λ_max_ (nm)	Emission ^1^ λ_max_ (nm)	Stokes Shift (cm^−1^)
**7b**	269, 322, 339, 357, 376	385, 406	621
**7c**	270, 323, 339, 356, 372, 393	416, 438	1472
**8c**	269, 363, 379, 400	408, 428	553
**13**	280, 302, 317, 330, 363, 383	404, 424	1420

^1^ Excited at 339 nm (**7b**, **7c**), 362 nm (**8c**) and 330 nm (**13**).

## Data Availability

Experimental details can be found in the Supporting Information. CCDC 1475147 contains the supplementary crystallographic data for this paper. These data can be obtained free of charge via www.ccdc.cam.ac.uk/data_request/cif,orby (accessed on 15 December 2021), emailing data_request@ccdc.cam.ac.uk or by contacting The Cambridge Crystallographic Data Centre, 12 Union Road, Cambridge, CB2 1EZ, UK; Fax: +44-1223-336033.

## Supplementary Materials

The following supporting information can be downloaded at: https://www.mdpi.com/article/10.3390/molecules28062474/s1. The ^1^H and ^13^C NMR spectra of obtained compounds. Atom coordinates (Å), total energy (Hartree) and the number of imaginary vibrational frequencies for the geometry of *cis*-**9b** and *trans*-**9b** optimized at the B3LYP/6-31++(d,p) level in the gas phase using Gaussian 09 (Tables S1 and S2). Atom coordinates (Å), total energy (Hartree) and the number of imaginary vibrational frequencies for the geometry of **5b**, **16a** and **16b** optimized at the B3LYP/6-311++(d,p) level in the gas phase using Gaussian 09 (Tables S3–S5). Refs. [42,49,50,51] are cited in the supplementary materials.

## References

[1] Yang Y., Börjesson K. (2022). Electroactive covalent organic frameworks: A new choice for organic electronics. Trends Chem..

[2] Koprowski M., Owsianik K., Knopik Ł., Vivek V., Romaniuk A., Różycka-Sokołowska E., Bałczewski P. (2022). Comprehensive Review on Synthesis, Properties, and Applications of Phosphorus (PIII, PIV, PV) Substituted Acenes with More Than Two Fused Benzene Rings. Molecules.

[3] Bałczewski P., Kowalska E., Różycka-Sokołowska E., Skalik J., Owsianik K., Koprowski M., Marciniak B., Guziejewski D., Ciesielski W. (2019). Mono-Aryl/Alkylthio-Substituted (Hetero)acenes of Exceptional Thermal and Photochemical Stability by the Thio-Friedel–Crafts/Bradsher Cyclization Reaction. Chem.—A Eur. J..

[4] Im Y., Byun S.Y., Kim J.H., Lee D.R., Oh C.S., Yook K.S., Lee J.Y. (2017). Recent Progress in High-Efficiency Blue-Light-Emitting Materials for Organic Light-Emitting Diodes. Adv. Funct. Mater..

[5] Yuan Q., Wang T., Yu P., Zhang H., Zhang H., Ji W. (2021). A review on the electroluminescence properties of quantum-dot light-emitting diodes. Org. Electron..

[6] Lim H., Woo S.-J., Ha Y.H., Kim Y.-H., Kim J.-J. (2022). Breaking the Efficiency Limit of Deep-Blue Fluorescent OLEDs Based on Anthracene Derivatives. Adv. Mater..

[7] Wang Z., Yang T., Dong S., Wen Z., Xu H., Miao Y., Wang H., Yu J. (2022). Anthracene and carbazole based asymmetric fluorescent materials for high-efficiency deep-blue non-doped organic light emitting devices with CIEy=0.06. Dye. Pigment..

[8] Haykir G., Aydemir M., Han S.H., Gumus S., Hizal G., Lee J.Y., Turksoy F. (2018). The investigation of sky-blue emitting anthracene-carbazole derivatives: Synthesis, photophysics and OLED applications. Org. Electron..

[9] Lim H., Cheon H.J., Lee G.S., Kim M., Kim Y.-H., Kim J.-J. (2019). Enhanced Triplet–Triplet Annihilation of Blue Fluorescent Organic Light-Emitting Diodes by Generating Excitons in Trapped Charge-Free Regions. ACS Appl. Mater. Interfaces.

[10] Song D., Yu Y., Yue L., Zhong D., Zhang Y., Yang X., Sun Y., Zhou G., Wu Z. (2019). Asymmetric thermally activated delayed fluorescence (TADF) emitters with 5,9-dioxa-13b-boranaphtho[3,2,1-de]anthracene (OBA) as the acceptor and highly efficient blue-emitting OLEDs. J. Mater. Chem. C.

[11] Park J., Jang B., Moon Y.J., Lee H., Kim Y.K., Yoon S.S. (2020). Efficient deep blue organic light emitting diodes based on [2,7,7,13,13-pentamethyl-9-(10-phenylanthracen-9-yl)-7,13-dihydrobenzo[5,6]-s-indaceno[1,2-g]quinoline] derivatives. Mol. Cryst. Liq. Cryst..

[12] Wang Y., Liu W., Ye S., Zhang Q., Duan Y., Guo R., Wang L. (2020). Molecular engineering of anthracene-based emitters for highly efficient nondoped deep-blue fluorescent OLEDs. J. Mater. Chem. C.

[13] Desai N.K., Kolekar G.B., Patil S.R. (2020). Fabrication and Characterization of Anthracene Doped P-terphenyl Thin Films By Spin Coating Technique; Investigation of Fluorescence Properties. J. Fluoresc..

[14] Lv X., Sun M., Xu L., Wang R., Zhou H., Pan Y., Zhang S., Sun Q., Xue S., Yang W. (2020). Highly efficient non-doped blue fluorescent OLEDs with low efficiency roll-off based on hybridized local and charge transfer excited state emitters. Chem. Sci..

[15] Huh J.-S., Ha Y.H., Kwon S.-K., Kim Y.-H., Kim J.-J. (2020). Design Strategy of Anthracene-Based Fluorophores toward High-Efficiency Deep Blue Organic Light-Emitting Diodes Utilizing Triplet–Triplet Fusion. ACS Appl. Mater. Interfaces.

[16] Wu Z., Zhu X., Li Y., Chen H., Zhuang Z., Shen P., Zeng J., Chi J., Ma D., Zhao Z. (2021). High-Performance Hybrid White OLEDs with Ultra-Stable Emission Color and Small Efficiency Roll-Off Achieved by Incorporating a Deep-Blue Fluorescent Neat Film. Adv. Opt. Mater..

[17] Kang S., Kwon H., Jeong J., Kim Y.-C., Park J. (2022). Synthesis and Electroluminescence Properties of New Blue Emitting Polymer Based on Dual-Core Type for Solution Process OLEDs. Macromol. Res..

[18] Zhao Z., Wang G., Luo X., Tian X., Zhang D., Guo S., Zhou H., Miao Y., Huang J., Wang H. (2022). Anthracene-based blue fluorescence materials utilized in non-doped OLEDs with high luminance and a low efficiency roll-off. Dye. Pigment..

[19] Lee J.H., Lin H.-Y., Chen C.-H., Lee Y.-T., Chiu T.-L., Lee J.-H., Chen C.-T., Adachi C. (2021). Deep Blue Fluorescent Material with an Extremely High Ratio of Horizontal Orientation to Enhance Light Outcoupling Efficiency (44%) and External Quantum Efficiency in Doped and Non-Doped Organic Light-Emitting Diodes. ACS Appl. Mater. Interfaces.

[20] Bałczewski P., Kowalska E., Różycka-Sokołowska E., Uznański P., Wilk J., Koprowski M., Owsianik K., Marciniak B. (2021). Organosulfur Materials with High Photo- and Photo-Oxidation Stability: 10-Anthryl Sulfoxides and Sulfones and Their Photophysical Properties Dependent on the Sulfur Oxidation State. Materials.

[21] Newman M.S., Hussain N.S. (1982). Synthesis of nuclear monobromobenz[a]anthracenes. J. Org. Chem..

[22] Andrus M.B., Ye Z., Zhang J. (2005). Highly selective glycine phase-transfer catalysis using fluoroanthracenylmethyl cinchonidine catalysts. Tetrahedron Lett..

[23] Harvey R.G., Cortez C., Sugiyama T., Ito Y., Sawyer T.W., DiGiovanni J. (1988). Biologically active dihydrodiol metabolites of polycyclic aromatic hydrocarbons structurally related to the potent carcinogenic hydrocarbon 7,12-dimethylbenz[a]anthracene. J. Med. Chem..

[24] Hallman J.L., Bartsch R.A. (1991). Synthesis of naphtho[f]ninhydrin. J. Org. Chem..

[25] Platt K.L., Oesch F. (1981). Reductive cyclization of keto acids to polycyclic aromatic hydrocarbons by hydroiodic acid-red phosphorus. J. Org. Chem..

[26] Ihmels H., Meiswinkel A., Mohrschladt C.J., Otto D., Waidelich M., Towler M., White R., Albrecht M., Schnurpfeil A. (2005). Anthryl-Substituted Heterocycles as Acid-Sensitive Fluorescence Probes. J. Org. Chem..

[27] Sangaiah R., Gold A., Toney G.E. (1983). Synthesis of a series of novel polycyclic aromatic systems: Isomers of benz[a]anthracene containing a cyclopenta-fused ring. J. Org. Chem..

[28] Newman M.S., Prabhu V.S., Veeraraghavan S. (1983). Synthesis of nuclear monobromobenz[a]anthracenes. J. Org. Chem..

[29] Bradsher C., Sinclair E. (1957). Notes—Cyclodehydrations in Liquid Sulfur Dioxide. J. Org. Chem..

[30] Yamato T., Sakaue N., Shinoda N., Matsuo K. (1997). Selective preparation of polycyclic aromatic hydrocarbons. Part 4.1 New synthetic route to anthracenes from diphenylmethanes using Friedel–Crafts intramolecular cyclization. J. Chem. Soc. Perkin Trans. 1.

[31] Bałczewski P., Skalik J., Uznański P., Guziejewski D., Ciesielski W. (2015). Use of isomeric, aromatic dialdehydes in the synthesis of photoactive, positional isomers of higher analogs of o-bromo(hetero)acenaldehydes. RSC Adv..

[32] Bodzioch A., Marciniak B., Różycka-Sokołowska E., Jeszka J.K., Uznański P., Kania S., Kuliński J., Bałczewski P. (2012). Synthesis and Optoelectronic Properties of Hexahydroxylated 10-O-R-Substituted Anthracenes via a New Modification of the Friedel–Crafts Reaction Using O-Protected ortho-Acetal Diarylmethanols. Chem.—A Eur. J..

[33] Bałczewski P., Bodzioch A., Różycka-Sokołowska E., Marciniak B., Uznański P. (2010). First Approach to Nitrogen-Containing Fused Aromatic Hydrocarbons as Targets for Organoelectronics Utilizing a New Transformation of O-Protected Diaryl Methanols. Chem.—A Eur. J..

[34] Bałczewski P., Kowalska E., Skalik J., Koprowski M., Owsianik K., Różycka-Sokołowska E. (2019). Ultrasound-assisted synthesis of RO- and RS-substituted (hetero)acenes via oxo- and thio-Friedel-Crafts/Bradsher reactions. Ultrason. Sonochem..

[35] Mondal E., Sahu P.R., Khan A.T. (2002). A Useful and Catalytic Method for Protection of Carbonyl Compounds into the Corresponding 1,3-Oxathiolanes and Deprotection to the Parent Carbonyl Compounds. Synlett.

[36] Djerassi C., Gorman M. (1953). Studies in Organic Sulfur Compounds. VI.1 Cyclic Ethylene and Trimethylene Hemithioketals. J. Am. Chem. Soc..

[37] Liang X., Gao S., Yang J., He M. (2008). Synthesis of a Novel Strong Brønsted Acidic Ionic Liquid and its Catalytic Activities for the Oxathioacetalization. Catal. Lett..

[38] Yus M., Nájera C., Foubelo F. (2003). The role of 1,3-dithianes in natural product synthesis. Tetrahedron.

[39] Morton D.R., Hobbs S.J. (1979). Facile preparation of cyclic ethylene thioketals and thioacetals with 2-phenyl- and 2-chloro-1,3,2-dithiaborolanes. J. Org. Chem..

[40] Fuji K., Ueda M., Sumi K., Kajiwara K., Fujita E., Iwashita T., Miura I. (1985). Chemistry of carbanions stabilized by sulfur. 1. Chemistry of 1,3-oxathianes. Synthesis and conformation of 2-substituted 1,3-oxathianes. J. Org. Chem..

[41] Satchell D.P.N., Satchell R.S. (1990). Mechanisms of hydrolysis of thioacetals. Chem. Soc. Rev..

[42] Lu T., Chen F. (2012). Multiwfn: A multifunctional wavefunction analyzer. J. Comput. Chem..

[43] Lu T., Chen F. (2012). Quantitative analysis of molecular surface based on improved Marching Tetrahedra algorithm. J. Mol. Graph. Model..

[44] (2015). ACD/Percepta.

[45] Míšek J., Vargas Jentzsch A., Sakurai S., Emery D., Mareda J., Matile S. (2010). A Chiral and Colorful Redox Switch: Enhanced π Acidity in Action. Angew. Chem. Int. Ed..

[46] Yang M., Xing Z., Fang B., Xie X., She X. (2020). Visible light photoredox catalyzed deprotection of 1,3-oxathiolanes. Org. Biom. Chem..

[47] Björk M., Grivas S. (2006). Synthesis of novel 2-aminoimidazo[4,5-b]pyridines, including the thieno analogue of the cooked-food mutagen IFP. J. Heterocycl. Chem..

[48] Sheldrick G.M. (2015). Crystal structure refinement with SHELXL. Acta Crystallogr..

[49] Johnson E.R., Keinan S., Mori-Sanchez P., Contreras-Garcia J., Cohen A.J., Yang W. (2010). Revealing noncovalent interactions. J. Am. Chem. Soc..

[50] Chemcraft—Graphical Software for Visualization of Quantum Chemistry Computations. https://www.chemcraftprog.com.

[51] Humphrey W., Dalke A., Schulten K. (1996). VMD—Visual Molecular Dynamics. J. Mol. Graph..

