# Supplementary material for: O,S-Acetals in a New Modification of oxo-Friedel–Crafts–Bradsher Cyclization—Synthesis of Fluorescent (Hetero)acenes and Mechanistic Considerations
Source: Molecules. 2023 Mar 8;28(6):2474. doi: 10.3390/molecules28062474 (PMC10051591; doi:10.3390/molecules28062474)
Supplement: Supplementary file 1 [file molecules-28-02474-s001.zip › molecules-2193431-supplementary.pdf]

Supplementary Materials (SM)

O,S-Acetals in a new modification of the *oxo*-Friedel-Crafts-Bradsher cyclization – synthesis of fluorescent (hetero)acenes and mechanistic considerations.

Krzysztof Owsianik [1,*], Ewa Różycka-Sokołowska [2*] and Piotr Bałczewski [1,2,*]

[1]Division of Organic Chemistry, Centre of Molecular and Macromolecular Studies, Polish Academy of Sciences, Sienkiewicza 112, Łódź, 90-363, Poland

[2]Institute of Chemistry, Faculty of Science and Technology, Jan Długosz University in Częstochowa, Armii Krajowej 13/15, Częstochowa, 42-201, Poland.

*Correspondence: krzysztof.owsianik@cbmm.lodz.pl (K.O.); e.sokolowska@ujd.edu.pl (E.R.-S.); piotr.balczewski@cbmm.lodz.pl (P.B.)

Table of contents

^{1}H and ^{13}C NMR spectra S2-S41

Crystal structure determination S42-S42

Calculations S43-43

Cartesian coordinates and total energies for investigated compounds S44-S50

References S50-S50

^{1}H and ^{13}C NMR spectra

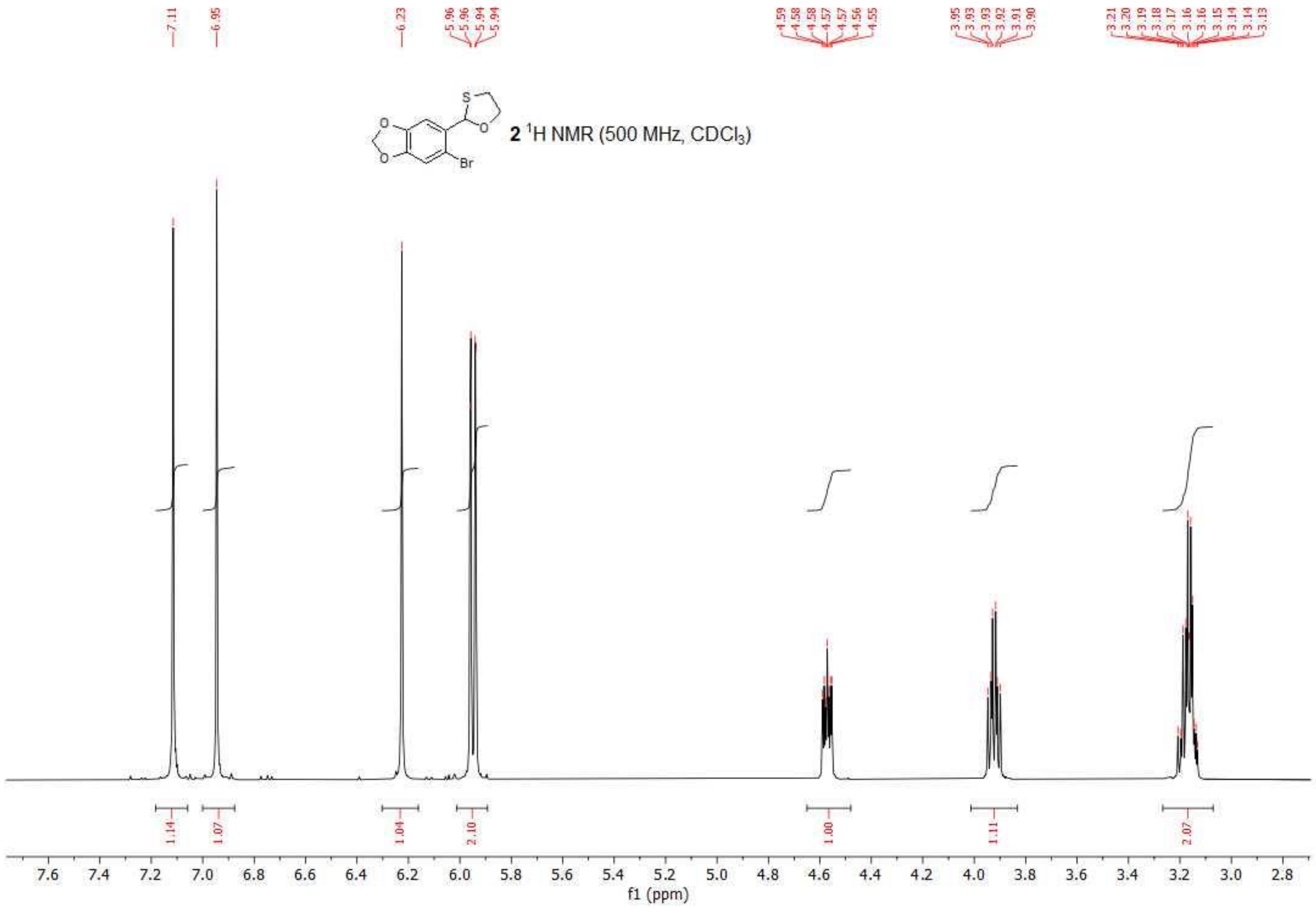

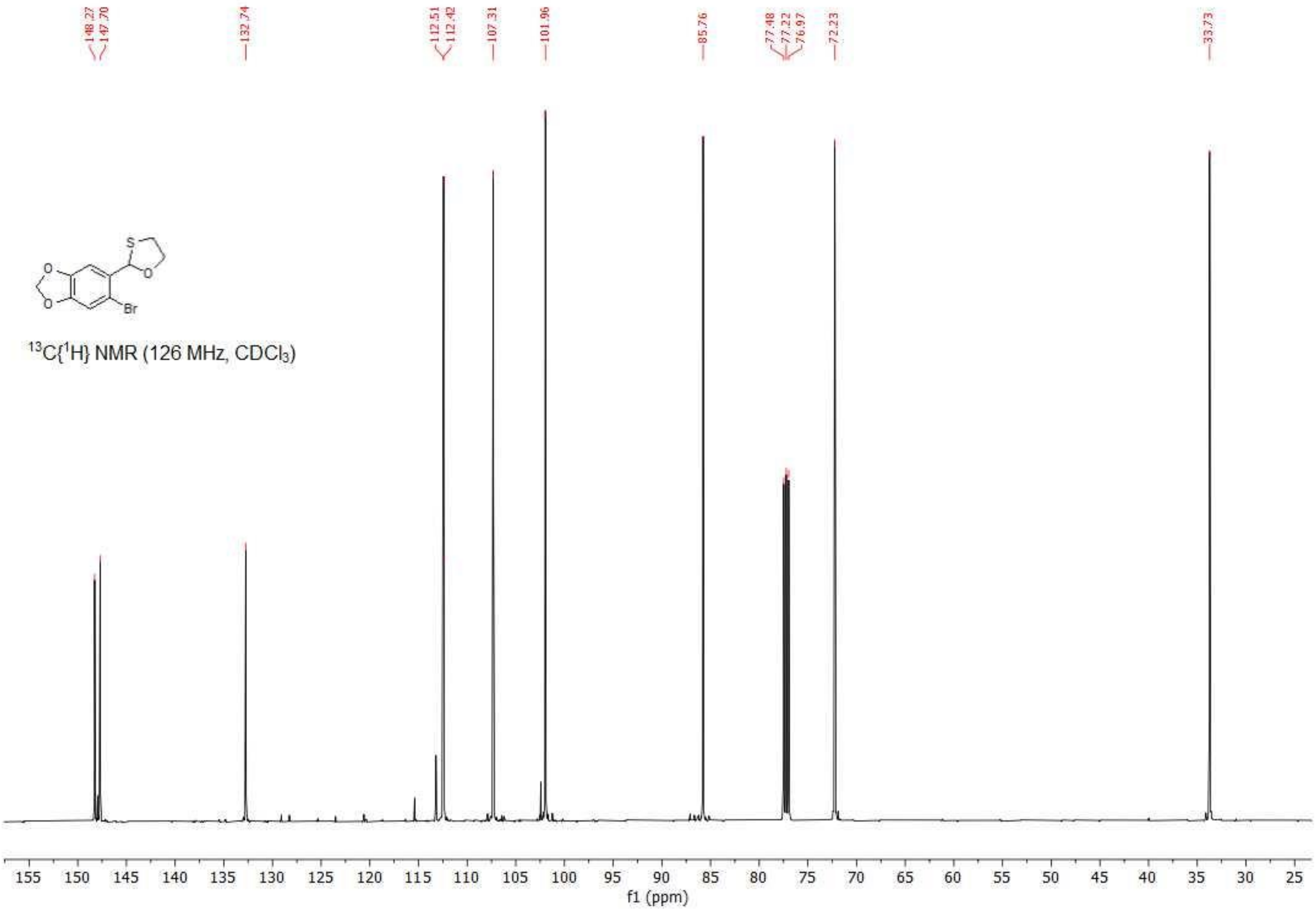
148.27
147.70
132.74
112.51
112.42
107.31
101.96
85.76
77.48
77.22
76.97
72.23
33.73
Br
13C{1H} NMR (126 MHz, CDCl3)
155 150 145 140 135 130 125 120 115 110 105 100 95 90 85 80 75 70 65 60 55 50 45 40 35 30 25
f1 (ppm)

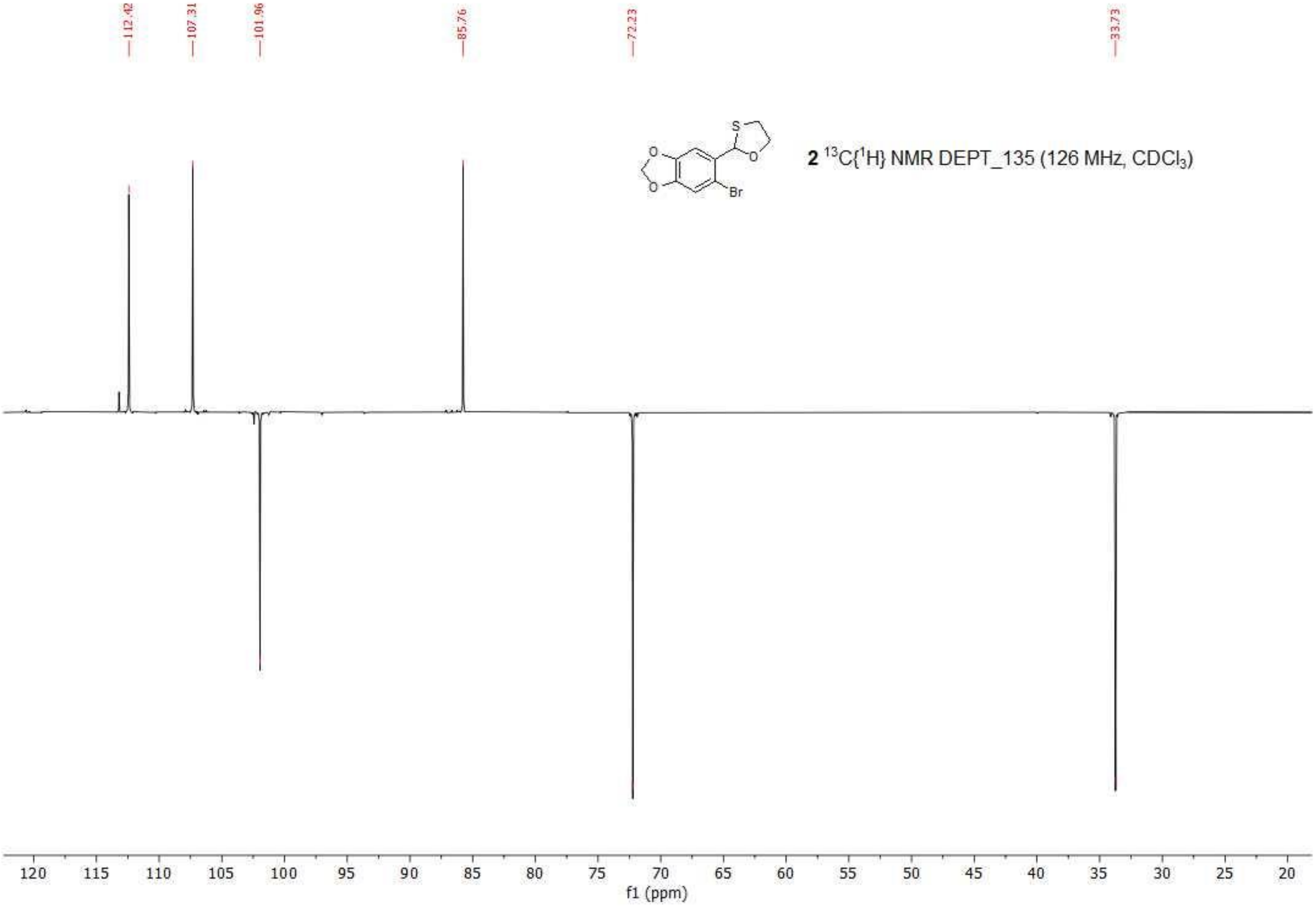
112.42
107.31
101.96
85.76
72.23
33.73
S
O
O
O
Br
2 $^{13}C\{^{1}H\}$ NMR DEPT_135 (126 MHz, $CDCl_3$)
120 115 110 105 100 95 90 85 80 75 70 65 60 55 50 45 40 35 30 25 20
f1 (ppm)

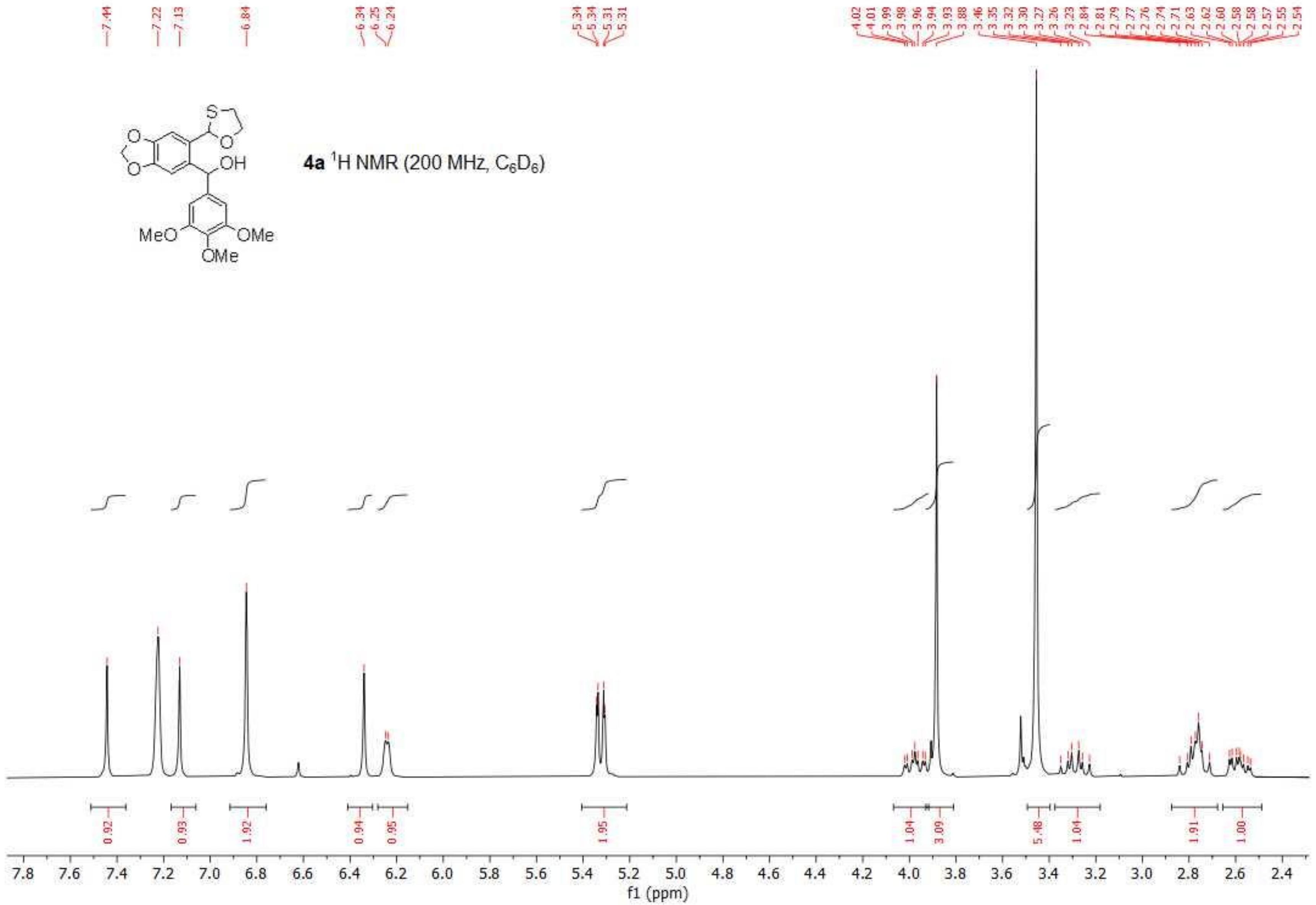

4a ^{1}H NMR (200 MHz, C_6D_6)
MeO
OMe
OMe
OH
f1 (ppm)

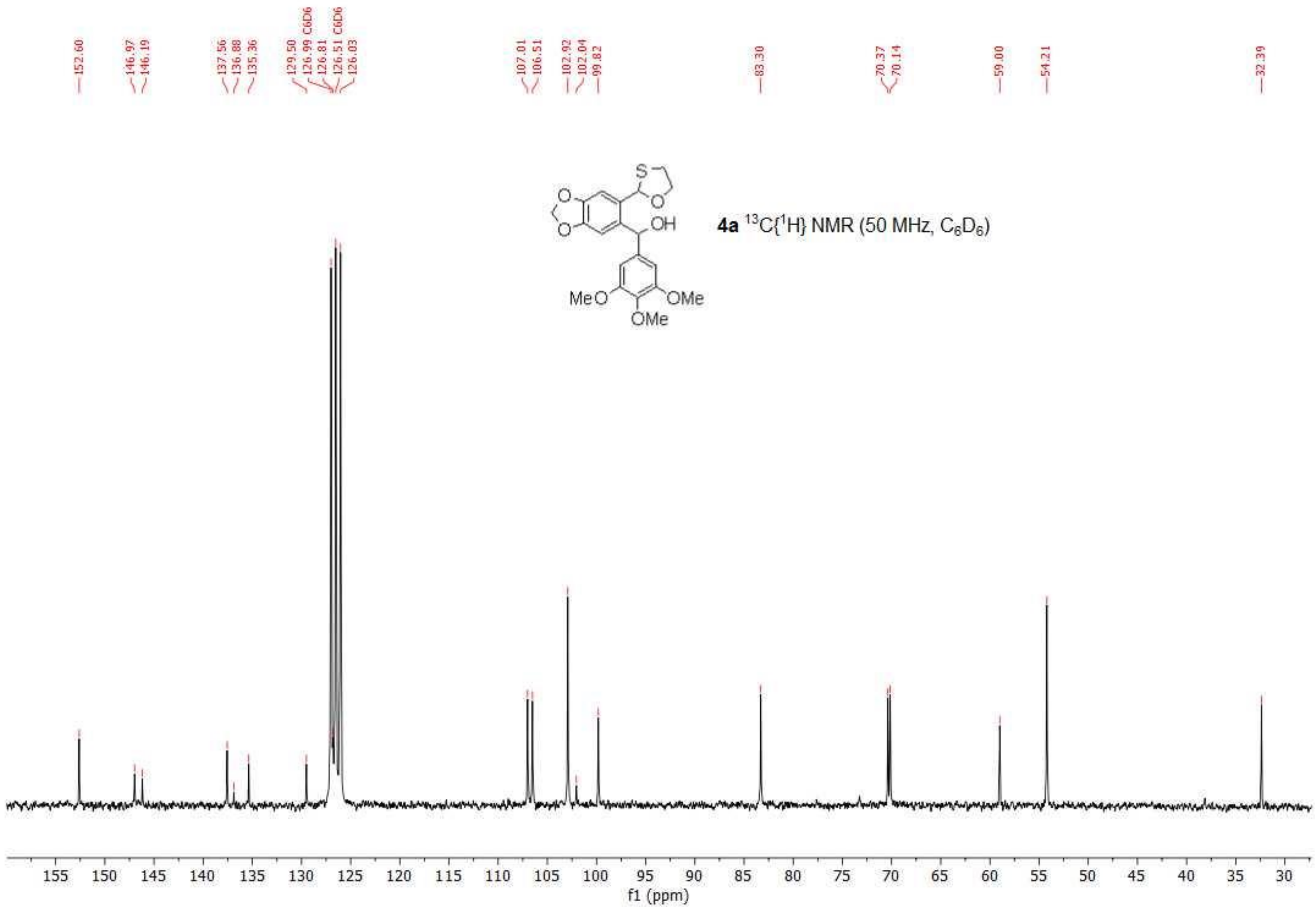
152.60
146.97
146.19
137.56
136.88
135.36
129.50
126.99 C6D6
126.81
126.51 C6D6
126.03
107.01
106.51
102.92
102.04
99.82
83.30
70.37
70.14
59.00
54.21
32.39
4a $^{13}C\{^{1}H\}$ NMR (50 MHz, C_6D_6)
S
O
OH
MeO
OMe
OMe
155 150 145 140 135 130 125 120 115 110 105 100 95 90 85 80 75 70 65 60 55 50 45 40 35 30
f1 (ppm)

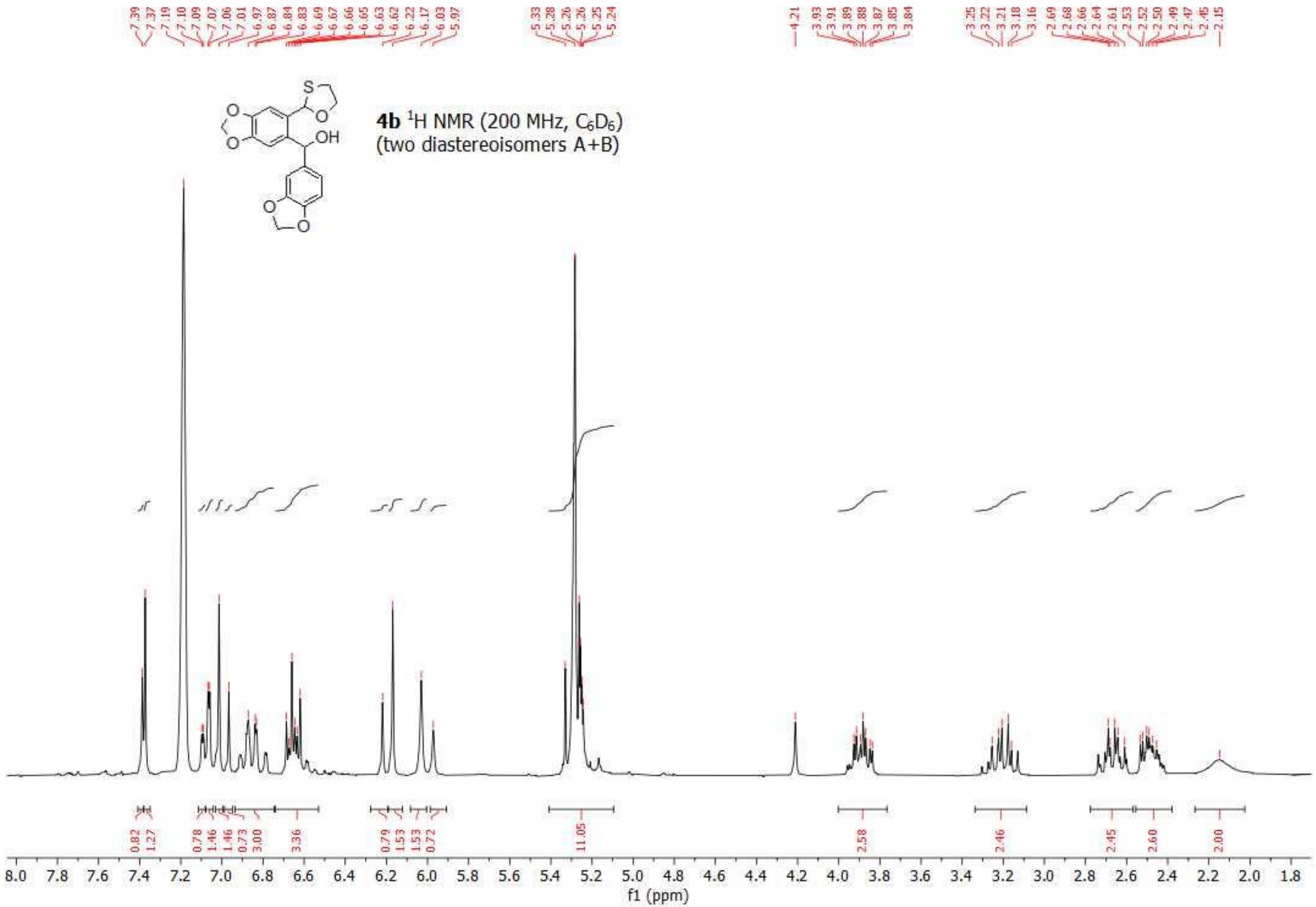
4b ¹H NMR (200 MHz, C₆D₆)
(two diastereoisomers A+B)
7.39 7.37 7.19 7.10 7.09 7.07 7.06 7.01 6.97 6.87 6.84 6.83 6.69 6.67 6.66 6.65 6.63 6.62 6.22 6.17 6.03 5.97
5.33 5.28 5.26 5.26 5.25 5.24
4.21 3.93 3.91 3.89 3.88 3.87 3.85 3.84
3.25 3.22 3.21 3.18 3.16 2.69 2.68 2.66 2.64 2.61 2.53 2.52 2.50 2.49 2.47 2.45 2.15
0.82 1.27 0.78 1.46 1.46 0.73 3.00 3.36 0.79 1.53 1.53 0.72 11.05 2.58 2.46 2.45 2.60 2.00
8.0 7.8 7.6 7.4 7.2 7.0 6.8 6.6 6.4 6.2 6.0 5.8 5.6 5.4 5.2 5.0 4.8 4.6 4.4 4.2 4.0 3.8 3.6 3.4 3.2 3.0 2.8 2.6 2.4 2.2 2.0 1.8
f1 (ppm)

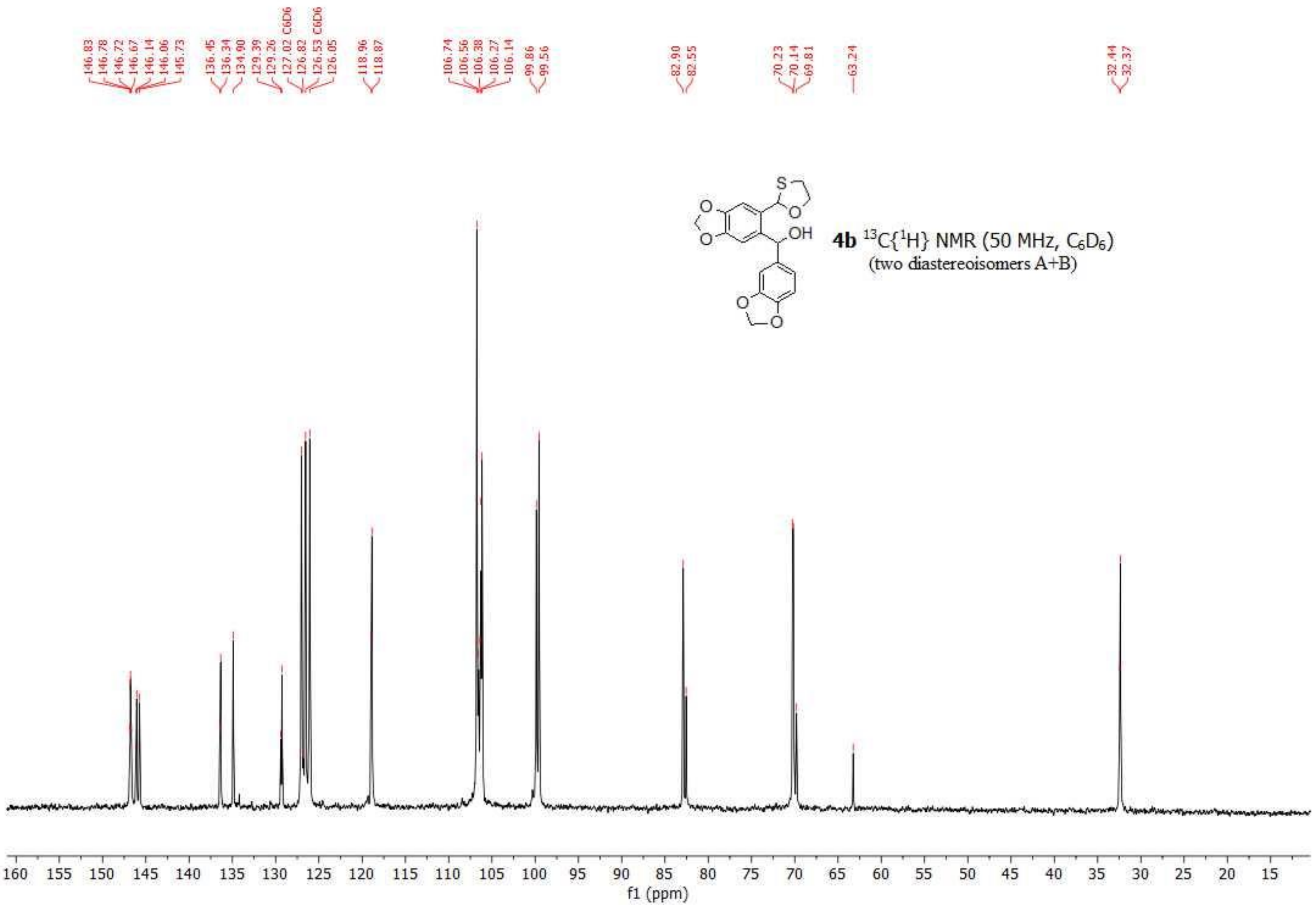

4b $^{13}C\{^{1}H\}$ NMR (50 MHz, C_6D_6)
(two diastereoisomers A+B)

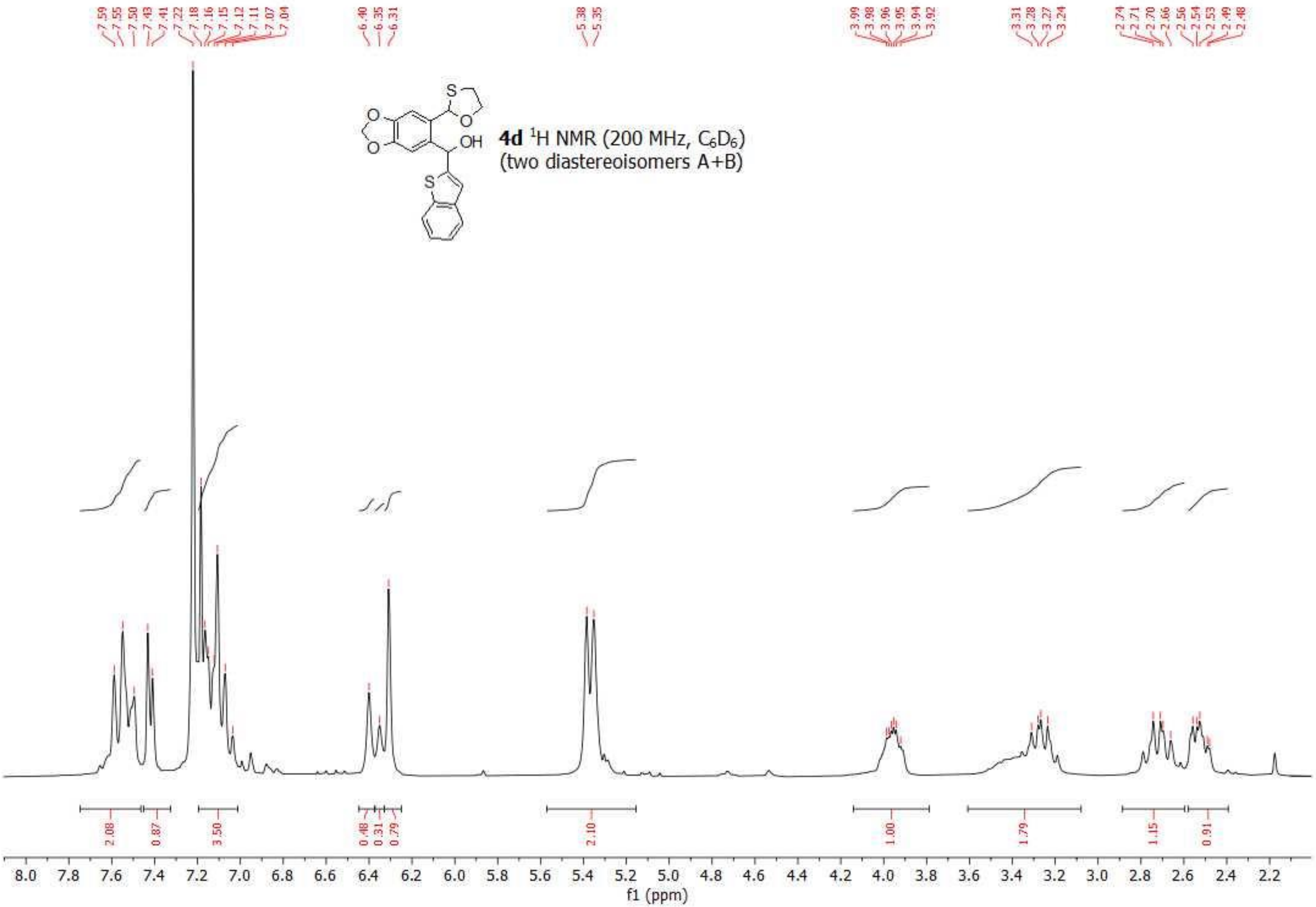
4d ¹H NMR (200 MHz, C6D6)
(two diastereoisomers A+B)
f1 (ppm)

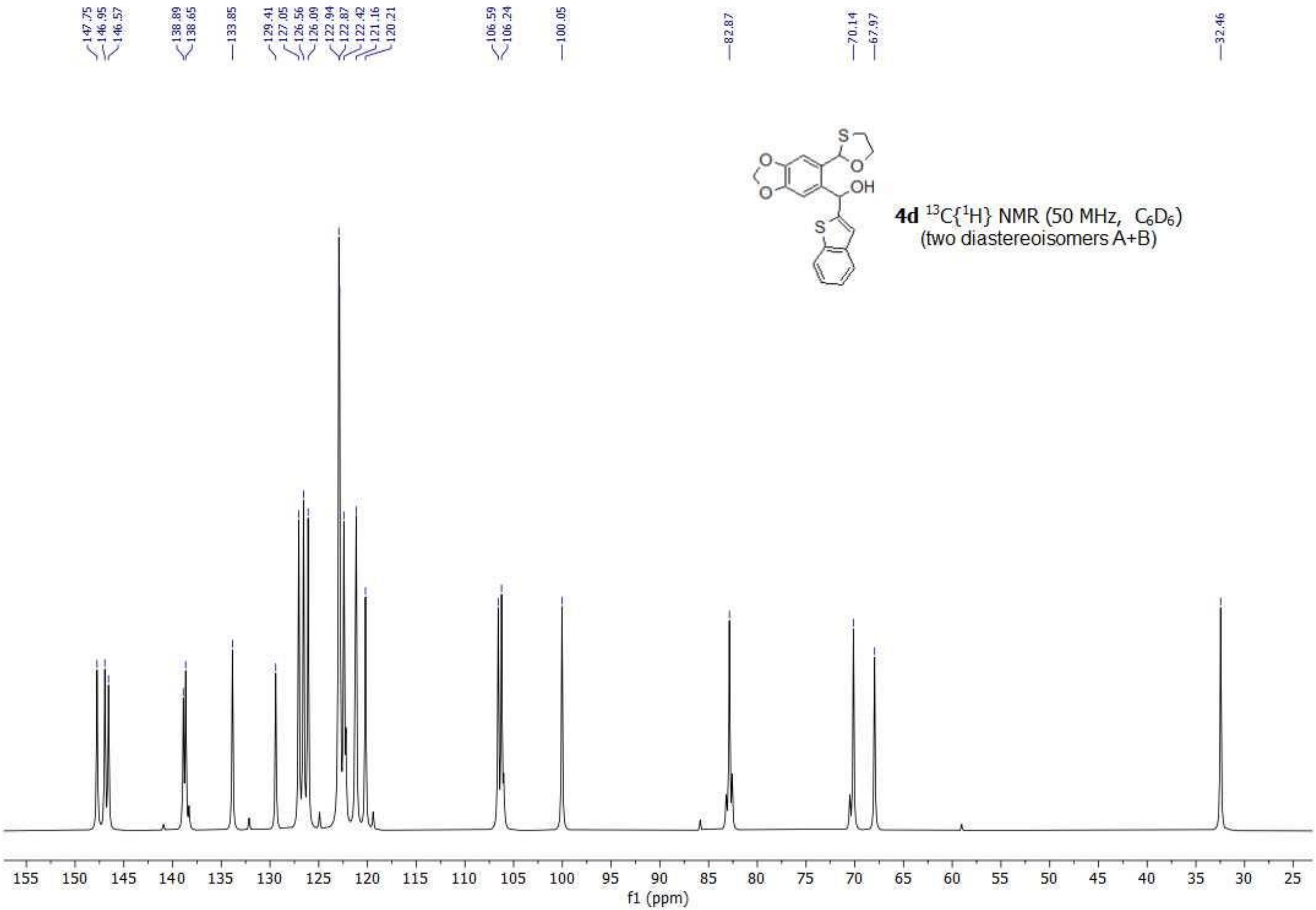
147.75
146.95
146.57
138.89
138.65
133.85
129.41
127.05
126.56
126.09
122.94
122.87
122.42
121.16
120.21
106.59
106.24
100.05
82.87
70.14
67.97
32.46
OH
4d $^{13}C\{^{1}H\}$ NMR (50 MHz, C_6D_6)
(two diastereoisomers A+B)
155 150 145 140 135 130 125 120 115 110 105 100 95 90 85 80 75 70 65 60 55 50 45 40 35 30 25
f1 (ppm)

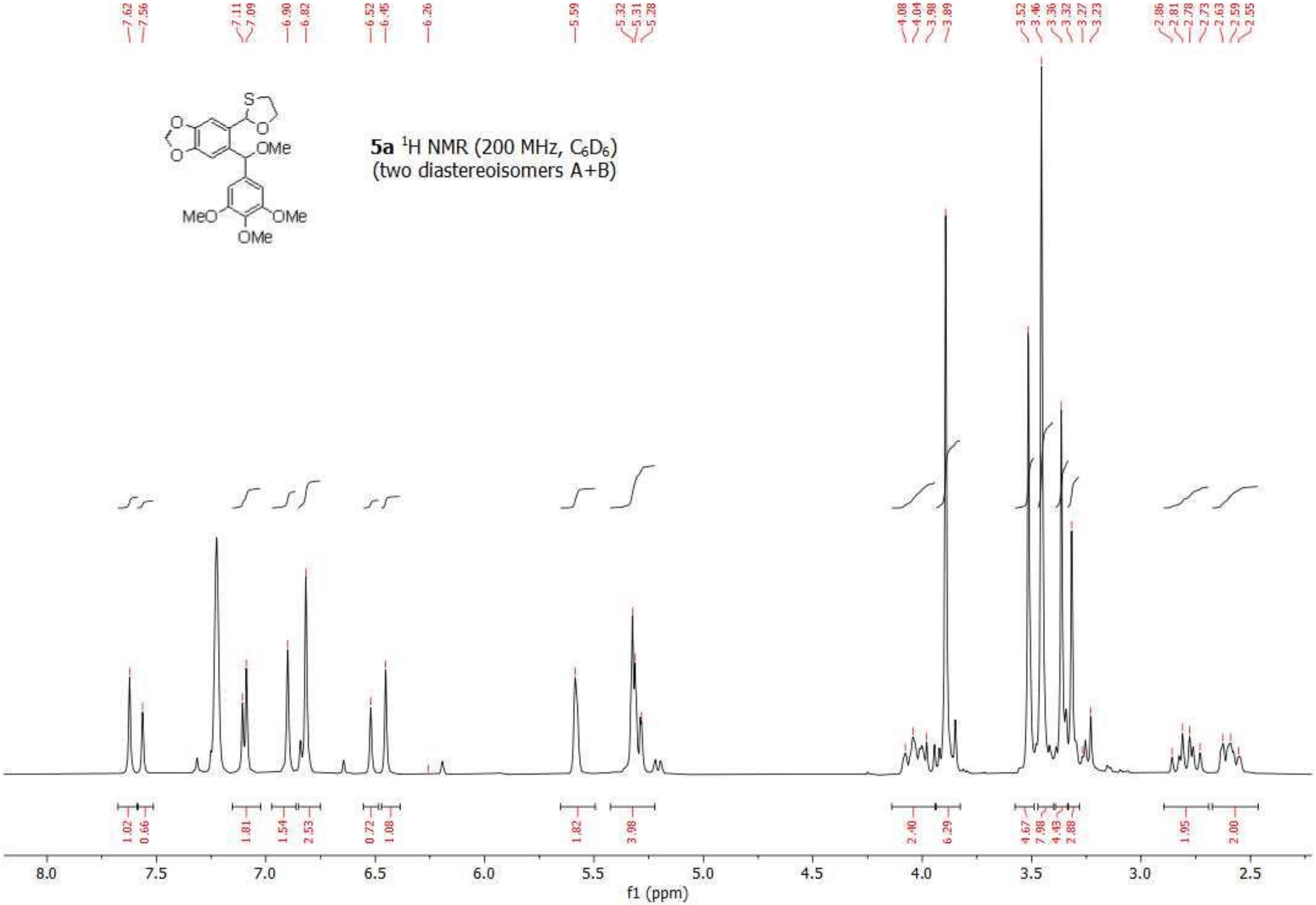

7.62
7.56
7.11
7.09
6.90
6.82
6.52
6.45
6.26
5.59
5.32
5.31
5.28
4.08
4.04
3.98
3.89
3.52
3.46
3.36
3.32
3.27
3.23
2.86
2.81
2.78
2.73
2.63
2.59
2.55
S
O
O
O
OMe
MeO
OMe
OMe
5a ^{1}H NMR (200 MHz, C_6D_6)
(two diastereoisomers A+B)
1.02
0.66
1.81
1.54
2.53
0.72
1.08
1.82
3.98
2.40
6.29
4.67
7.98
4.43
2.88
1.95
2.00
8.0
7.5
7.0
6.5
6.0
5.5
5.0
4.5
4.0
3.5
3.0
2.5
f1 (ppm)

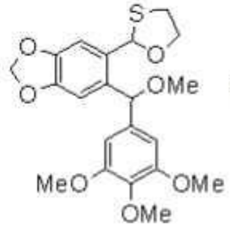

5a $^{13}C\{^{1}H\}$ NMR (50 MHz, $CDCl_3$)
(two diastereoisomers A+B)

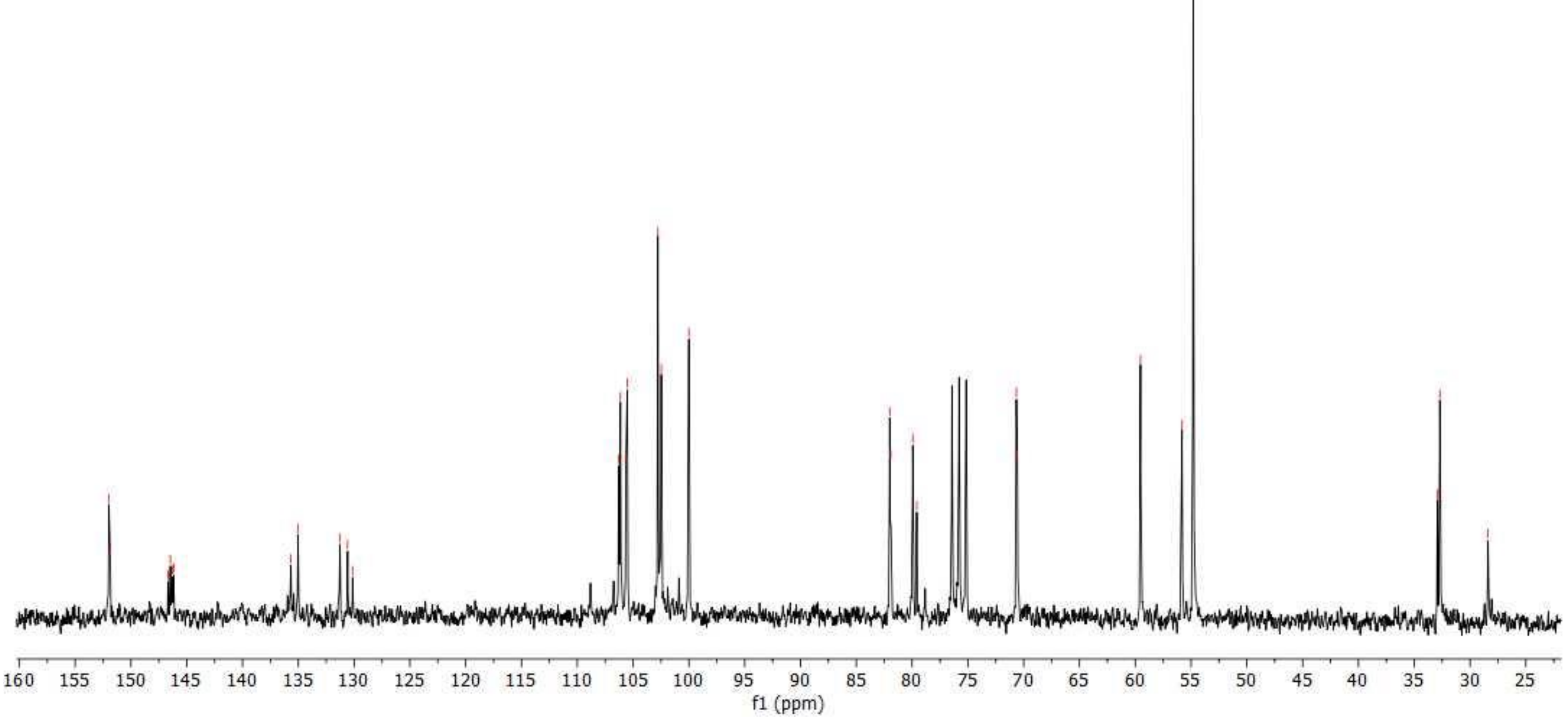

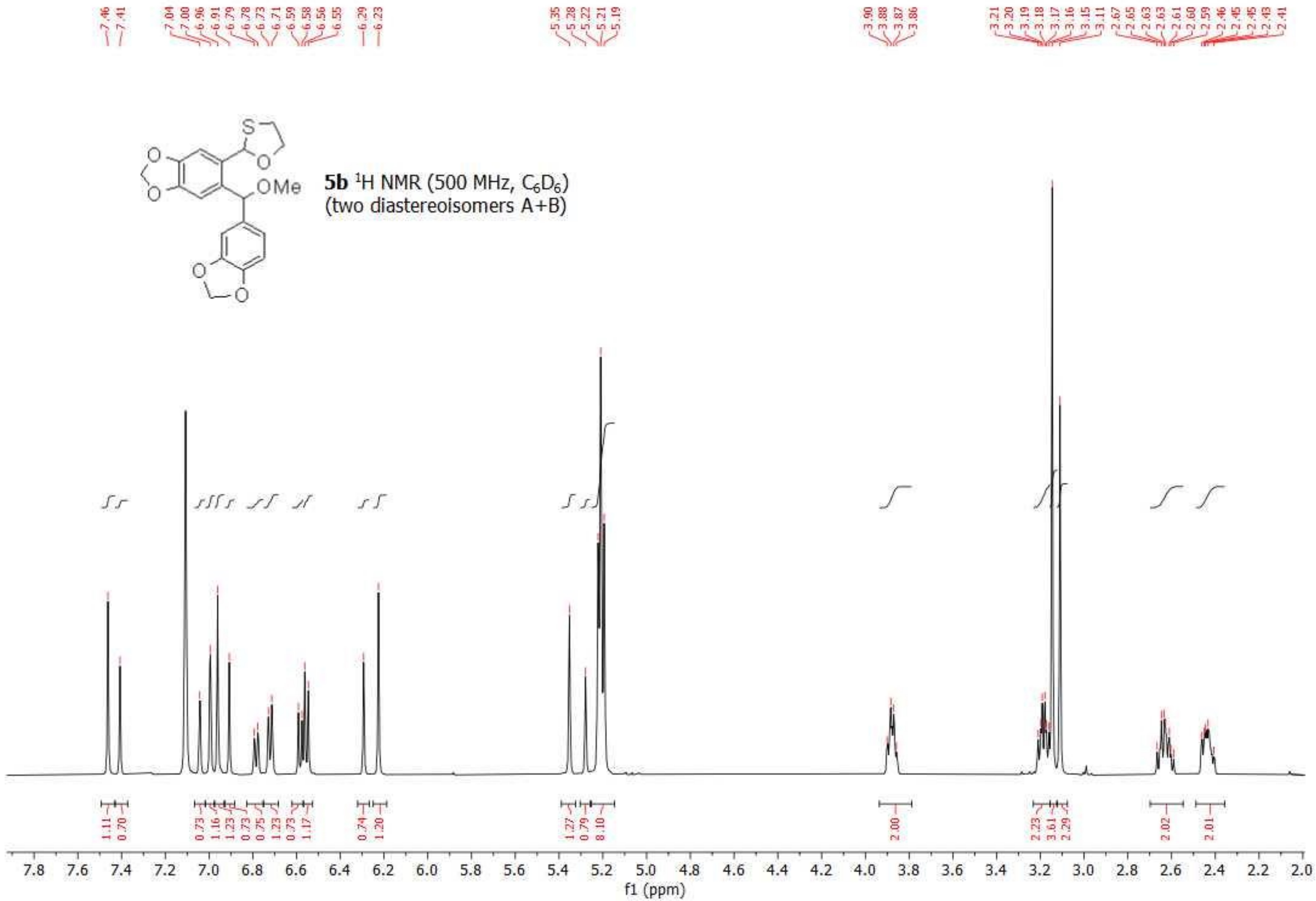
OMe
5b ¹H NMR (500 MHz, C6D6)
(two diastereoisomers A+B)
f1 (ppm)

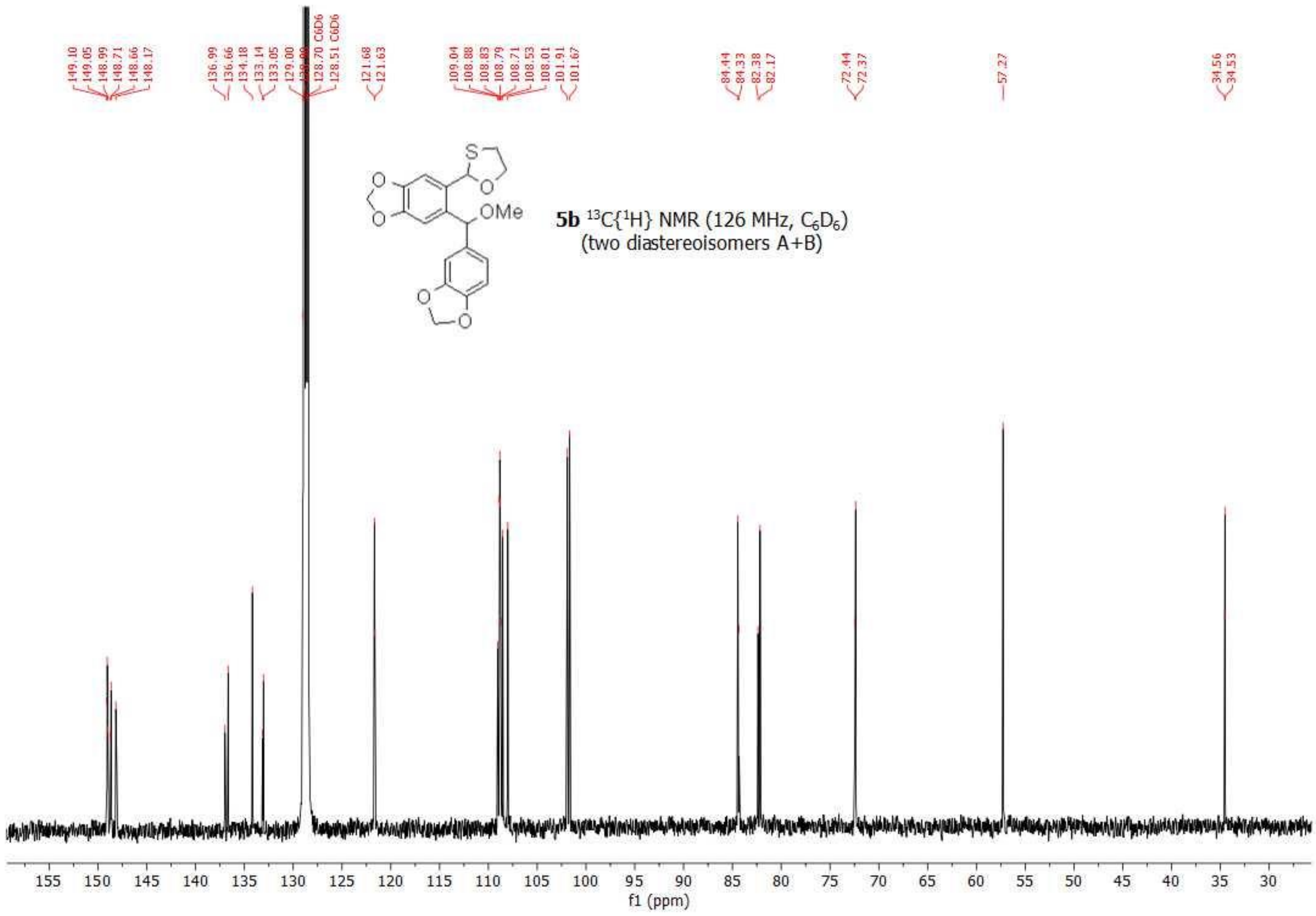
149.10
149.05
148.99
148.71
148.66
148.17
136.99
136.66
134.18
133.14
133.05
129.00
128.70 C6D6
128.51 C6D6
121.68
121.63
109.04
108.88
108.83
108.79
108.71
108.53
108.01
101.91
101.67
84.44
84.33
82.38
82.17
72.44
72.37
57.27
34.56
34.53
OMe
S
O
5b 13C{1H} NMR (126 MHz, C6D6)
(two diastereoisomers A+B)
155 150 145 140 135 130 125 120 115 110 105 100 95 90 85 80 75 70 65 60 55 50 45 40 35 30
f1 (ppm)

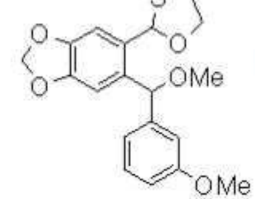

5c ^{1}H NMR (500 MHz, $CDCl_3$)
(two diastereoisomers A+B)

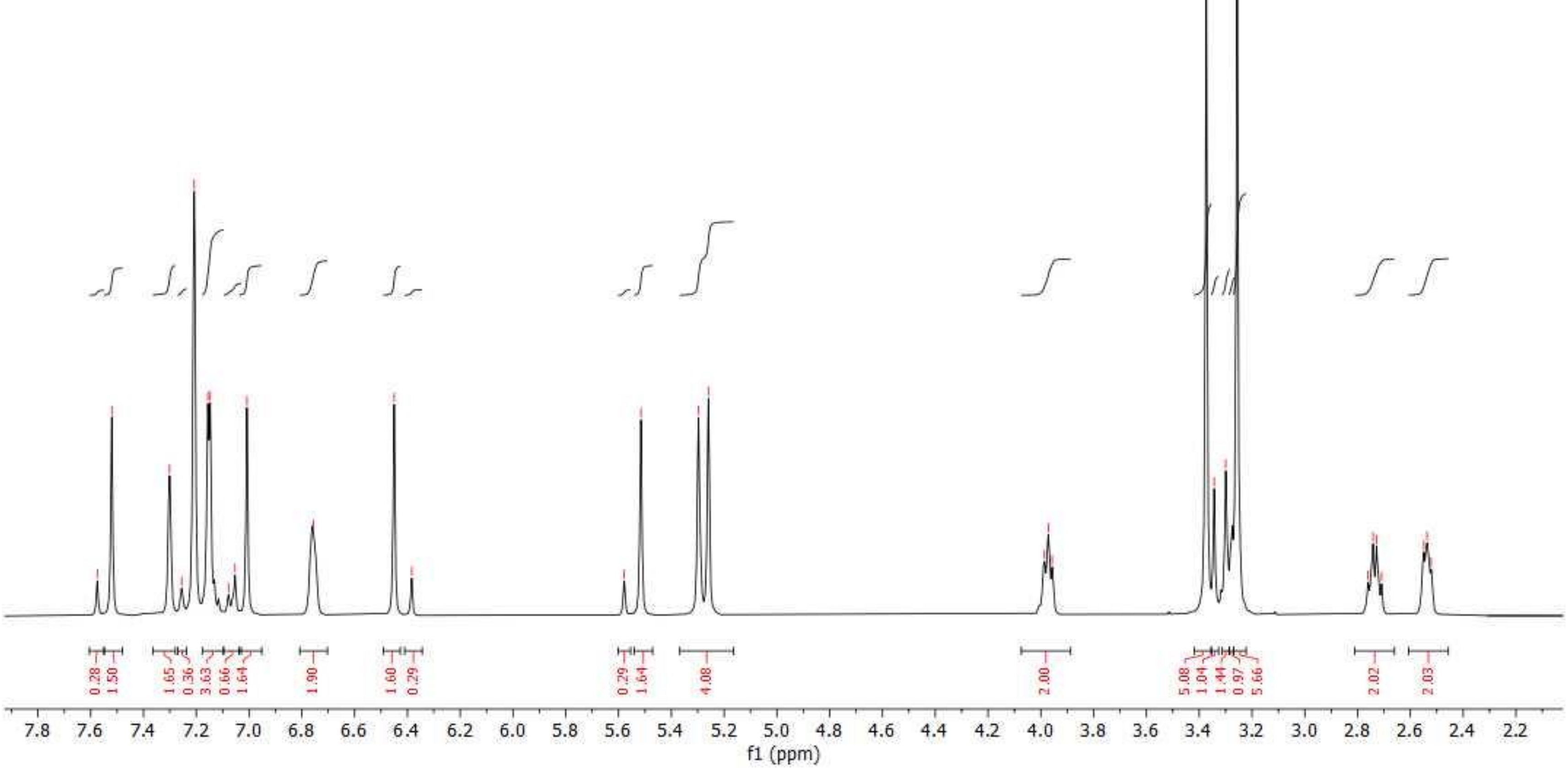

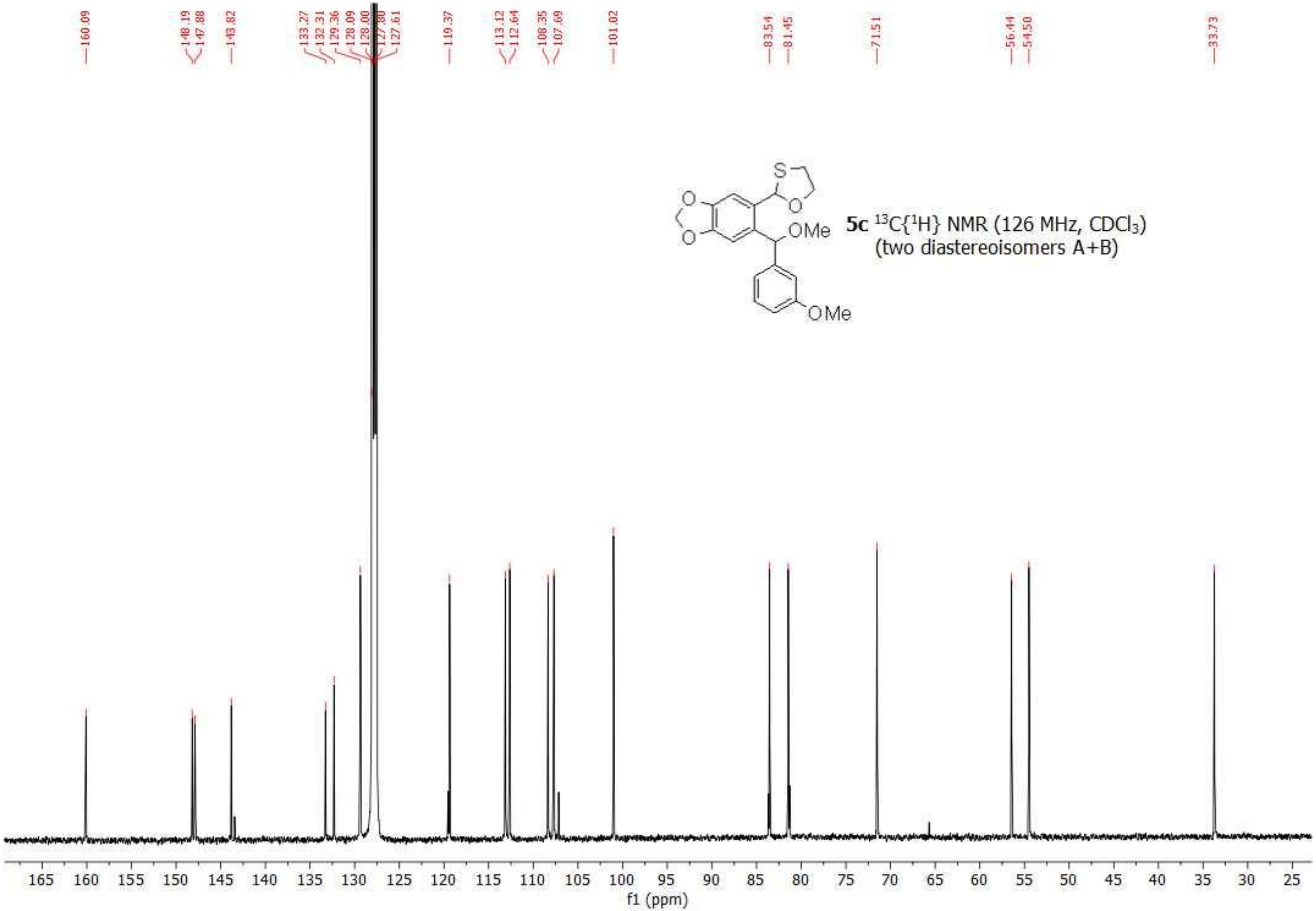
160.09
148.19
147.88
143.82
133.27
132.31
129.36
128.09
128.00
127.80
127.61
119.37
113.12
112.64
108.35
107.69
101.02
83.54
81.45
71.51
56.44
54.50
33.73
OMe
OMe
5c $^{13}C\{^1H\}$ NMR (126 MHz, $CDCl_3$)
(two diastereoisomers A+B)
165 160 155 150 145 140 135 130 125 120 115 110 105 100 95 90 85 80 75 70 65 60 55 50 45 40 35 30 25
f1 (ppm)

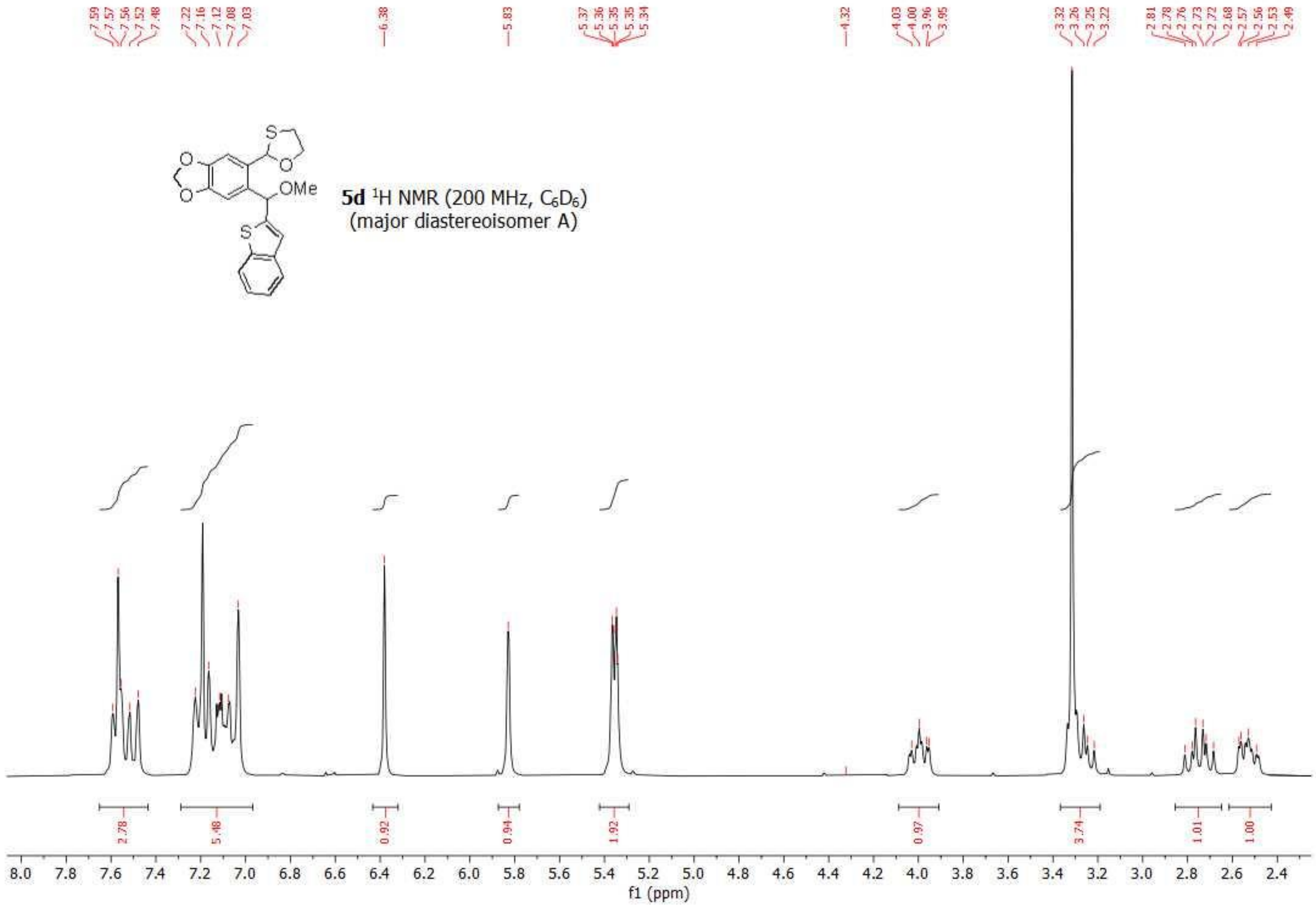

5d ¹H NMR (200 MHz, C6D6)
(major diastereoisomer A)
OMe
f1 (ppm)

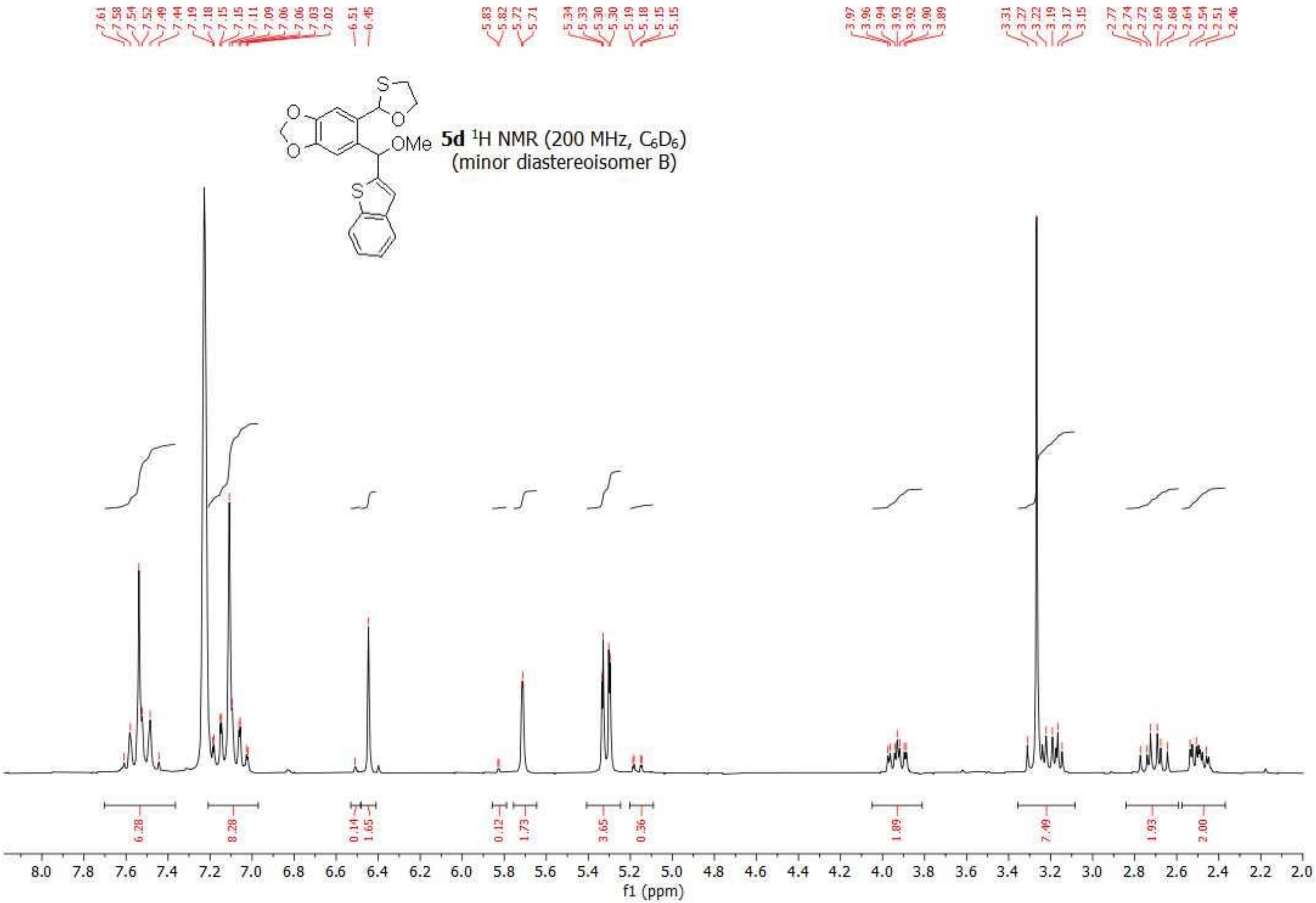

5d ^{1}H NMR (200 MHz, C_6D_6)
(minor diastereoisomer B)
OMe
f1 (ppm)

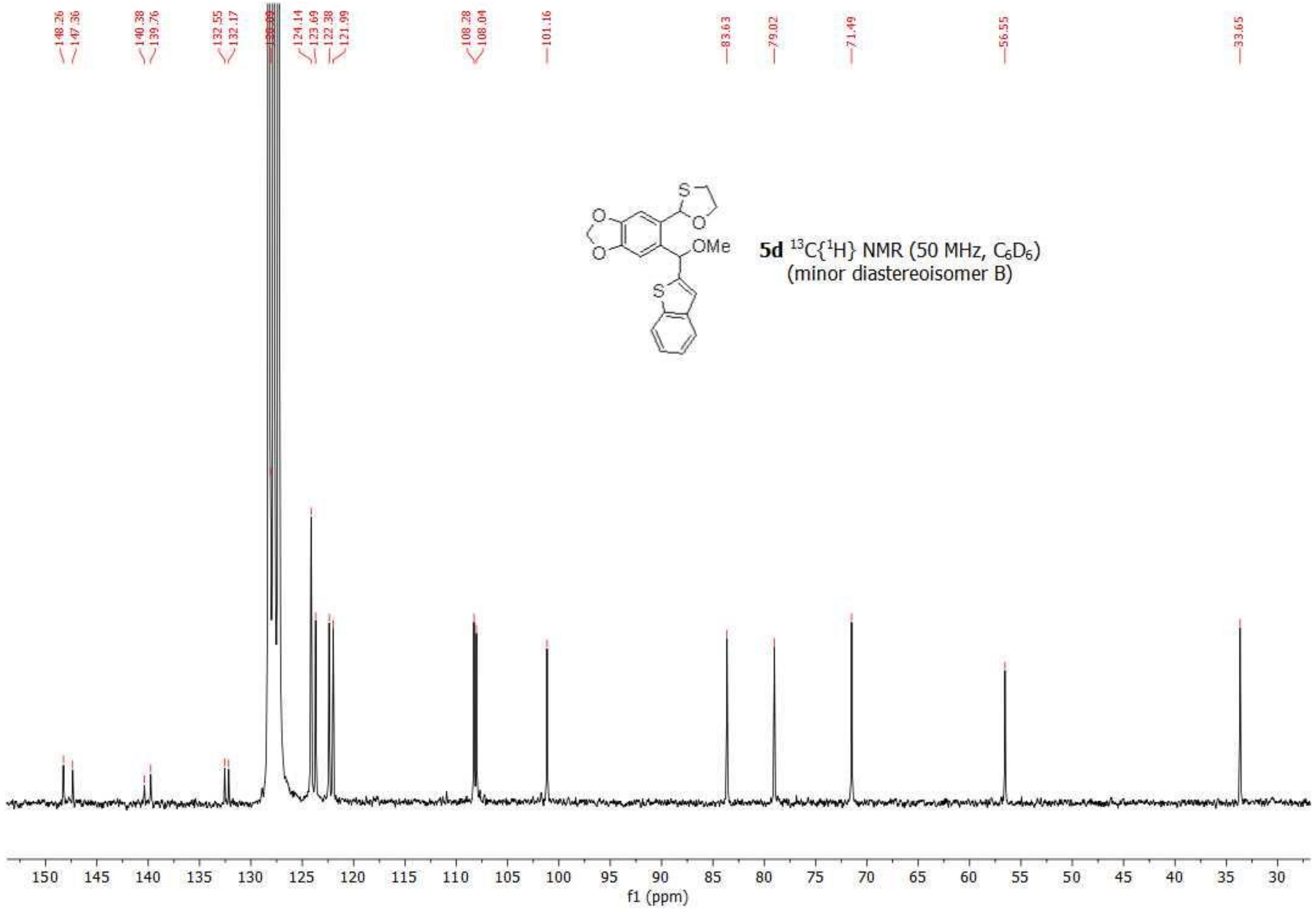

5d $^{13}C\{^{1}H\}$ NMR (50 MHz, C_6D_6)
(minor diastereoisomer B)

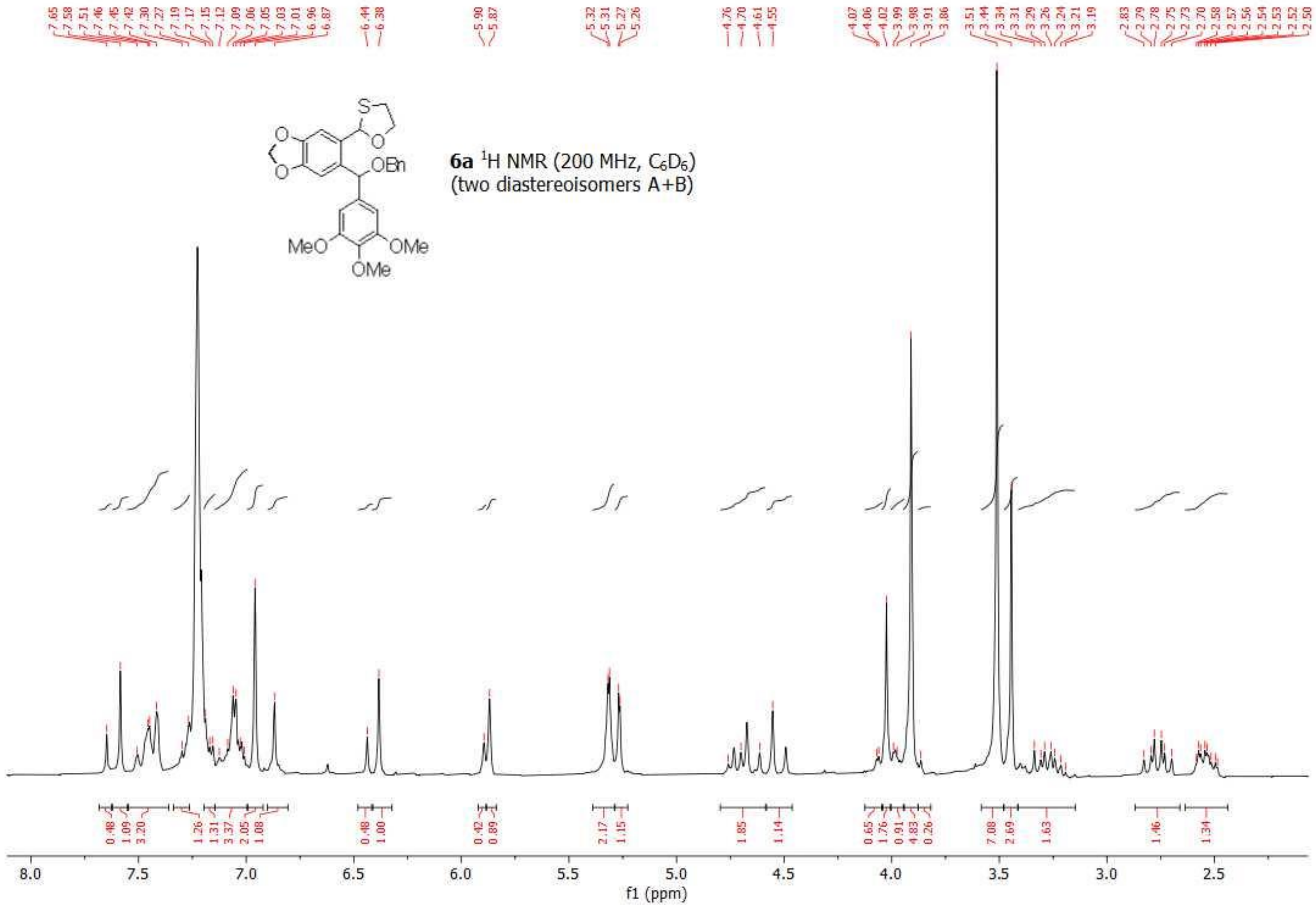
OBn
MeO
OMe
OMe
6a 1H NMR (200 MHz, C6D6)
(two diastereoisomers A+B)
8.0
7.5
7.0
6.5
6.0
5.5
5.0
4.5
4.0
3.5
3.0
2.5
f1 (ppm)

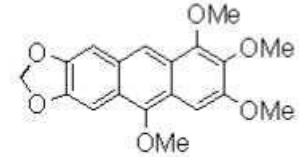

7a ^{1}H NMR (200 MHz, C_6D_6)

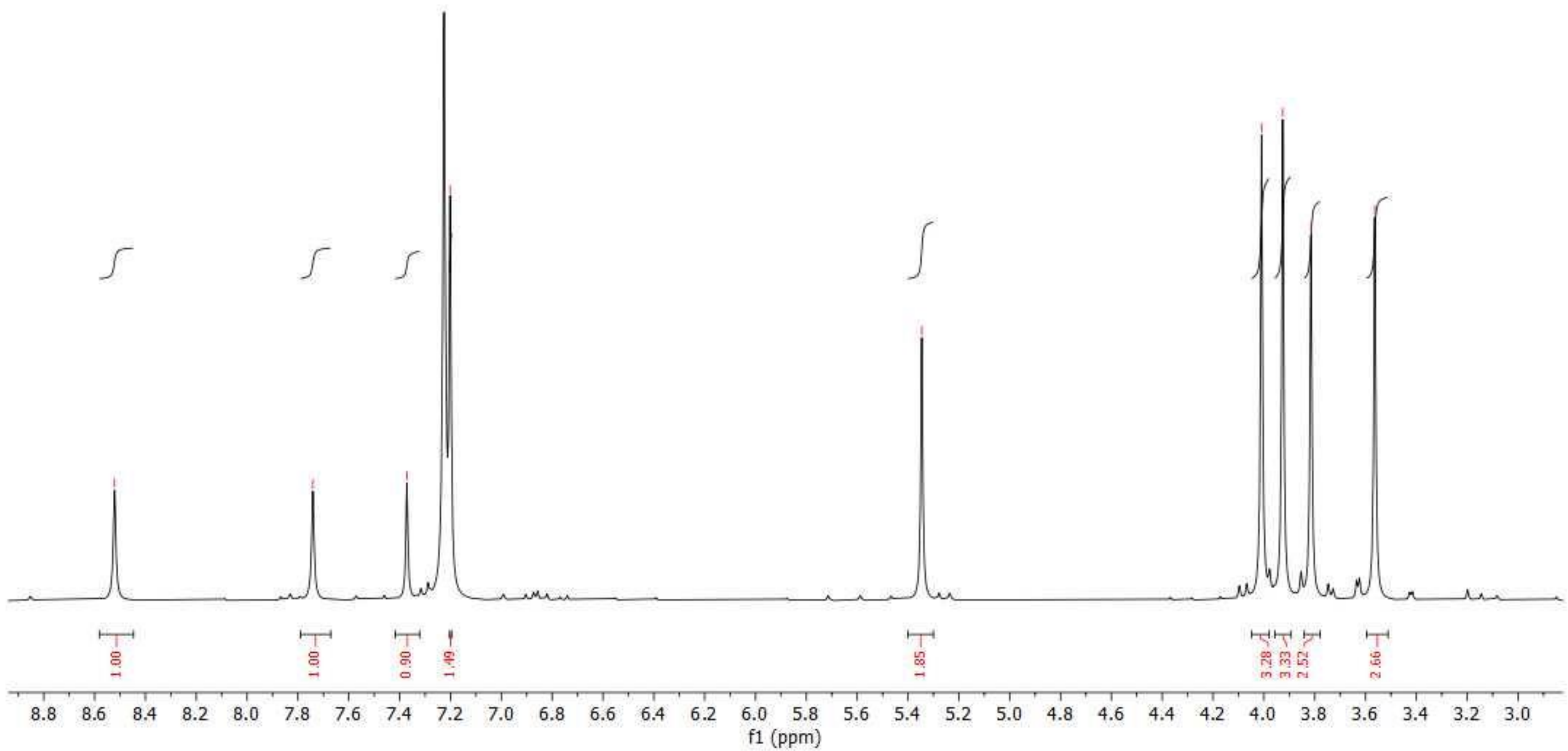

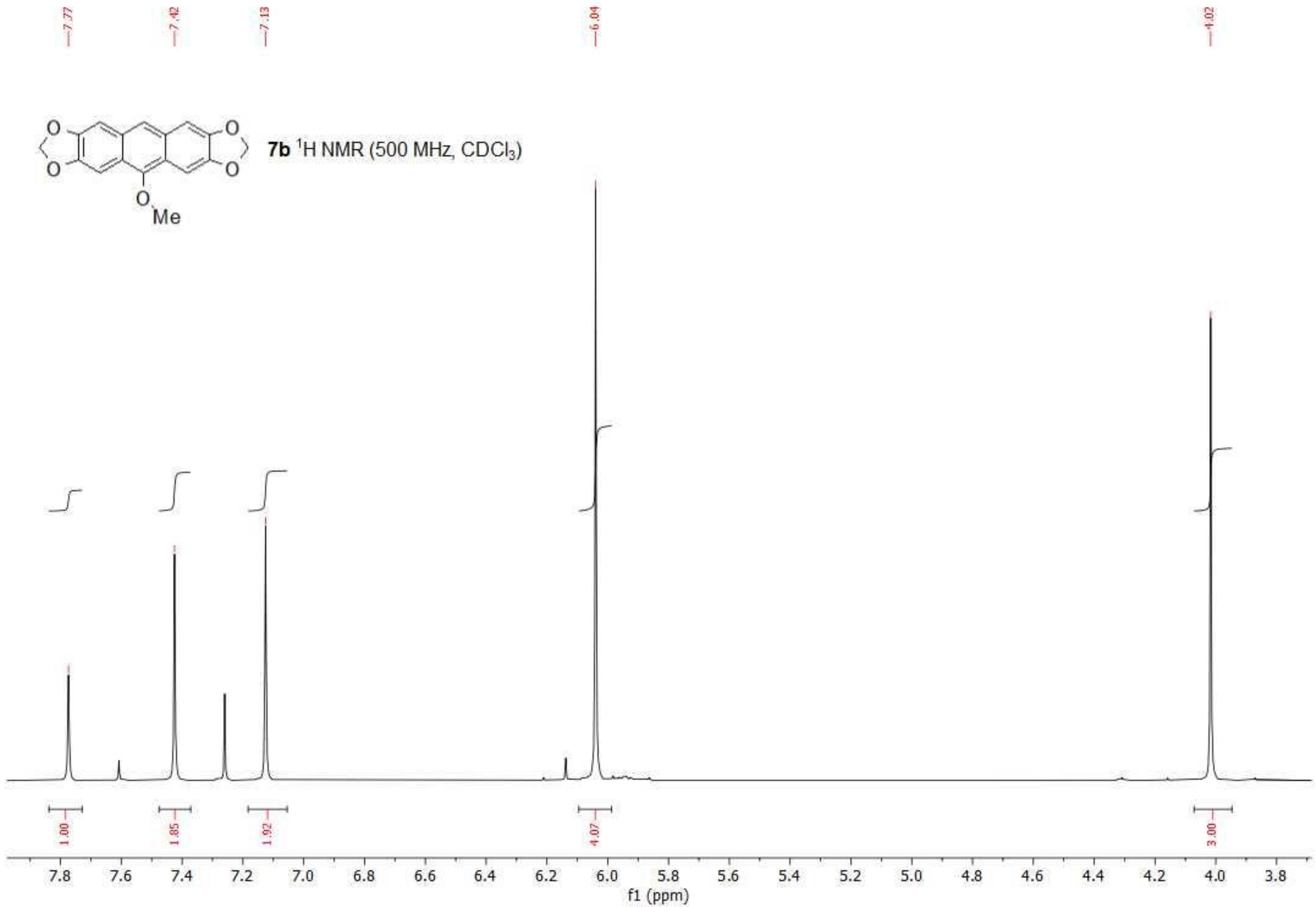

7.77
7.42
7.13
6.04
4.02
7b ¹H NMR (500 MHz, CDCl3)
Me
1.00
1.85
1.92
4.07
3.00
7.8 7.6 7.4 7.2 7.0 6.8 6.6 6.4 6.2 6.0 5.8 5.6 5.4 5.2 5.0 4.8 4.6 4.4 4.2 4.0 3.8
f1 (ppm)

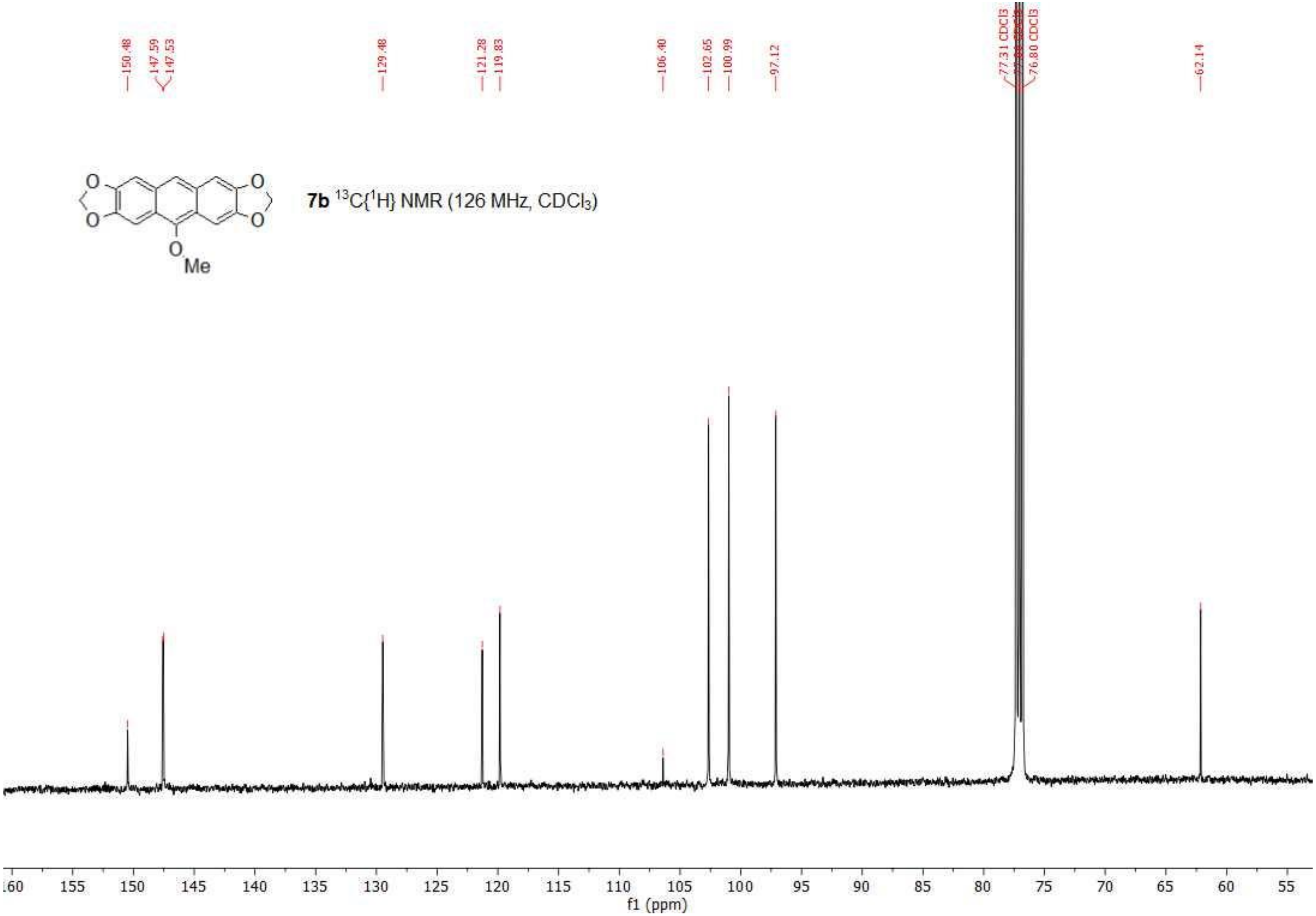

7b $^{13}C\{^1H\}$ NMR (126 MHz, $CDCl_3$)

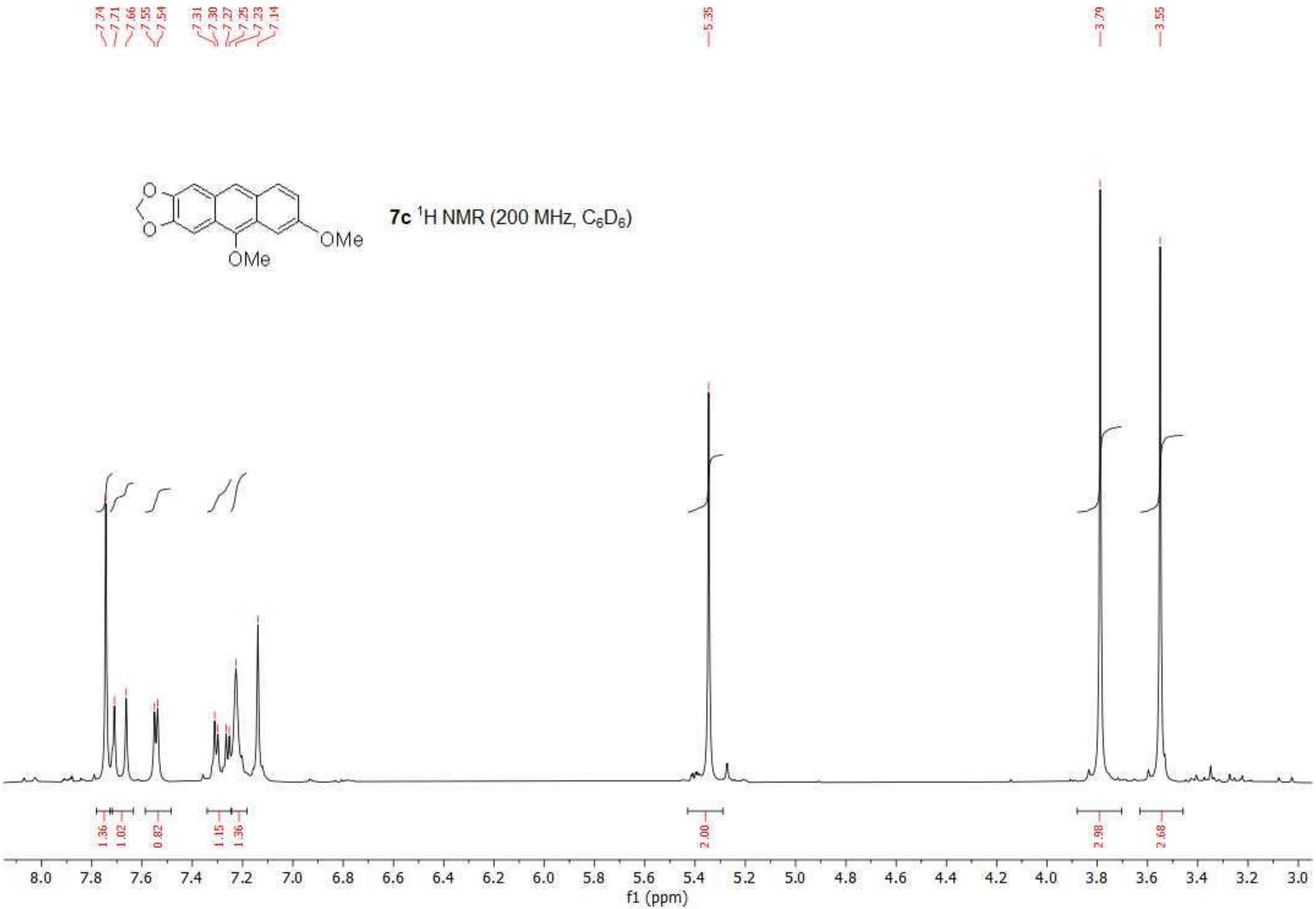
7.74
7.71
7.66
7.55
7.54
7.31
7.30
7.27
7.25
7.23
7.14
5.35
3.79
3.55
OMe
OMe
7c ^{1}H NMR (200 MHz, C_6D_6)
1.36
1.02
0.82
1.15
1.36
2.00
2.98
2.68
8.0 7.8 7.6 7.4 7.2 7.0 6.8 6.6 6.4 6.2 6.0 5.8 5.6 5.4 5.2 5.0 4.8 4.6 4.4 4.2 4.0 3.8 3.6 3.4 3.2 3.0
f1 (ppm)

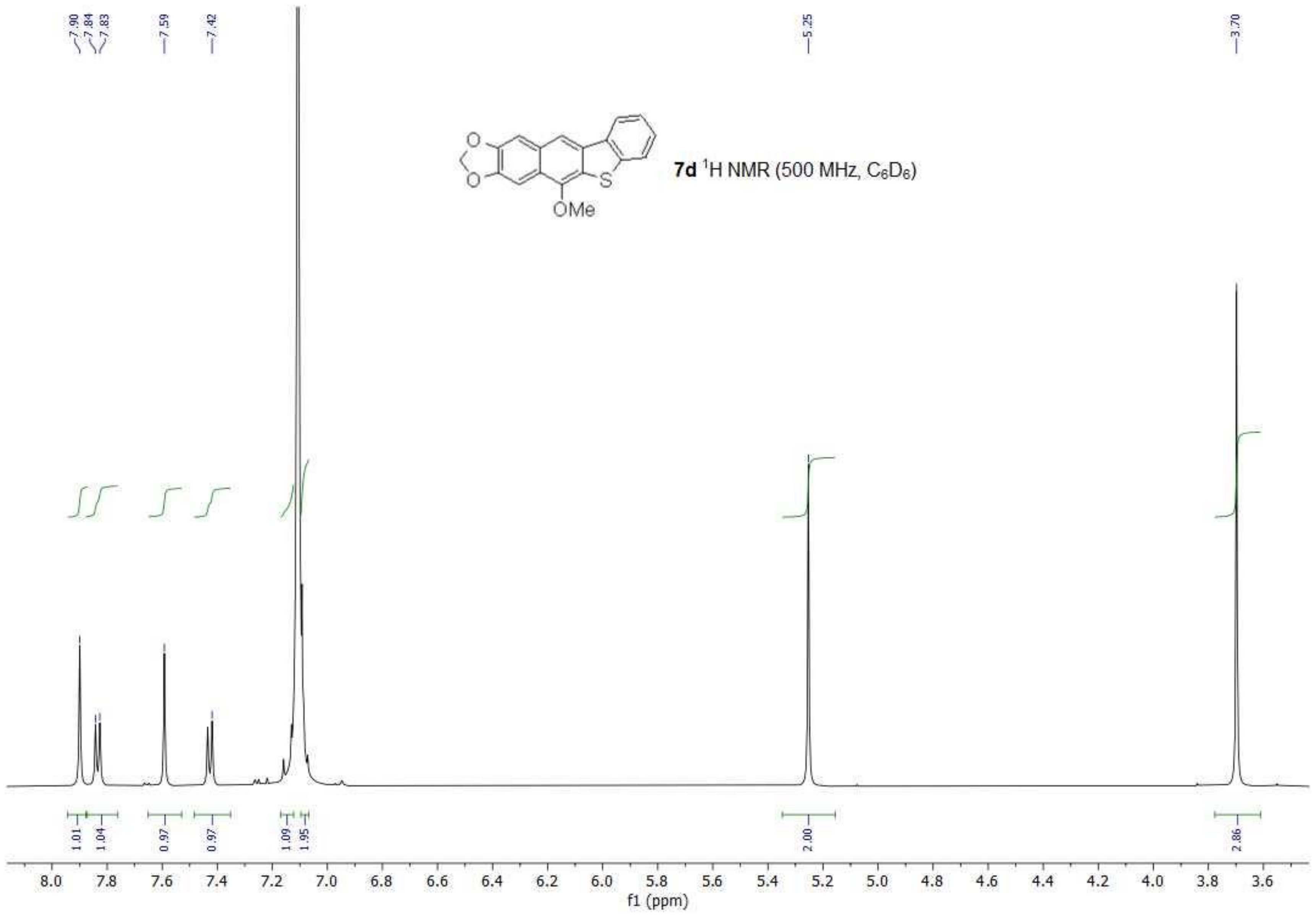
7.90
7.84
7.83
7.59
7.42
5.25
3.70
OMe
S
O
O
7d ^{1}H NMR (500 MHz, C_6D_6)
1.01
1.04
0.97
0.97
1.09
1.95
2.00
2.86
8.0 7.8 7.6 7.4 7.2 7.0 6.8 6.6 6.4 6.2 6.0 5.8 5.6 5.4 5.2 5.0 4.8 4.6 4.4 4.2 4.0 3.8 3.6
f1 (ppm)

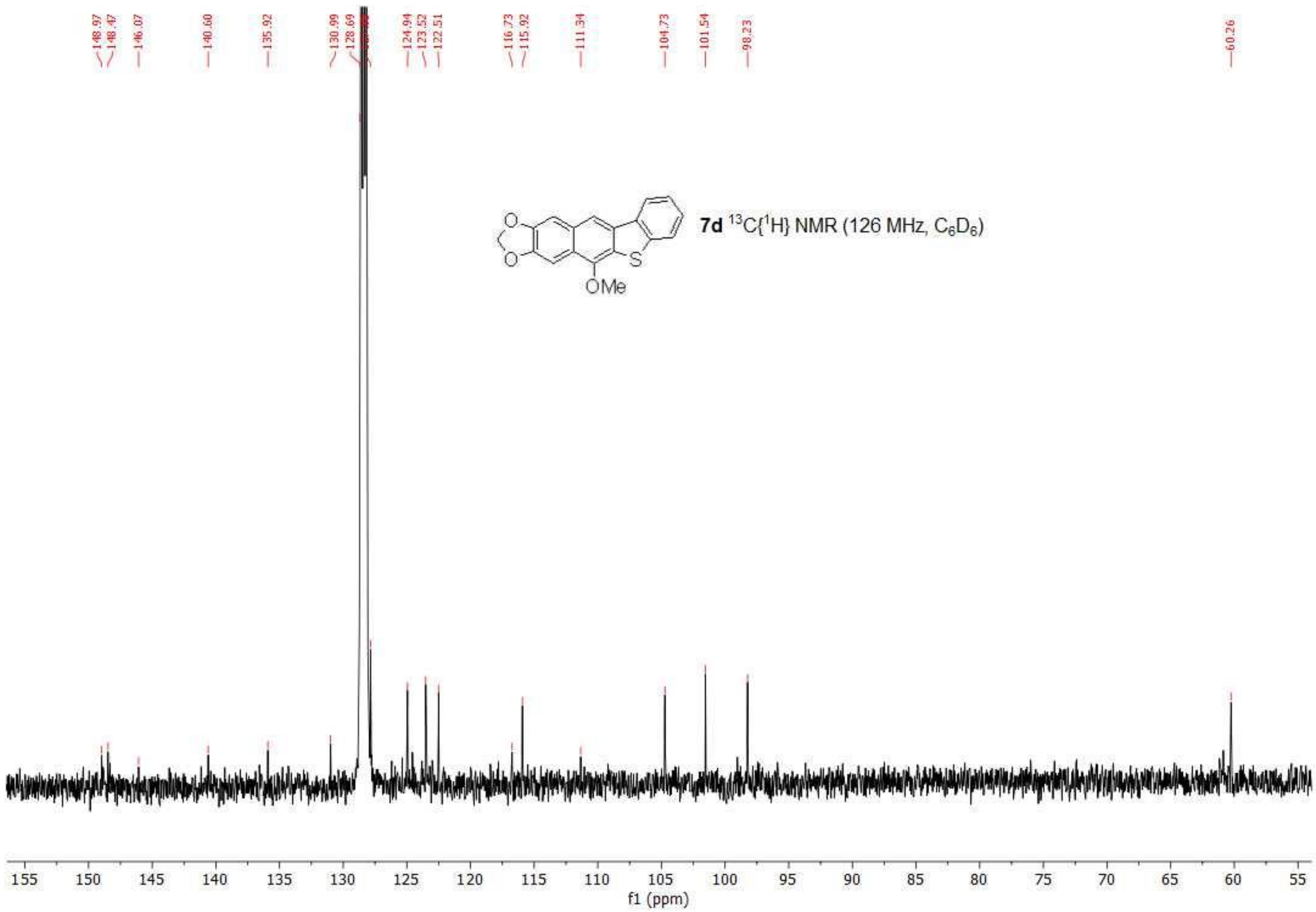
148.97
148.47
146.07
140.60
135.92
130.99
128.69
124.94
123.52
122.51
116.73
115.92
111.34
104.73
101.54
98.23
60.26
O
O
S
OMe
7d $^{13}C\{^1H\}$ NMR (126 MHz, C_6D_6)
155 150 145 140 135 130 125 120 115 110 105 100 95 90 85 80 75 70 65 60 55
f1 (ppm)

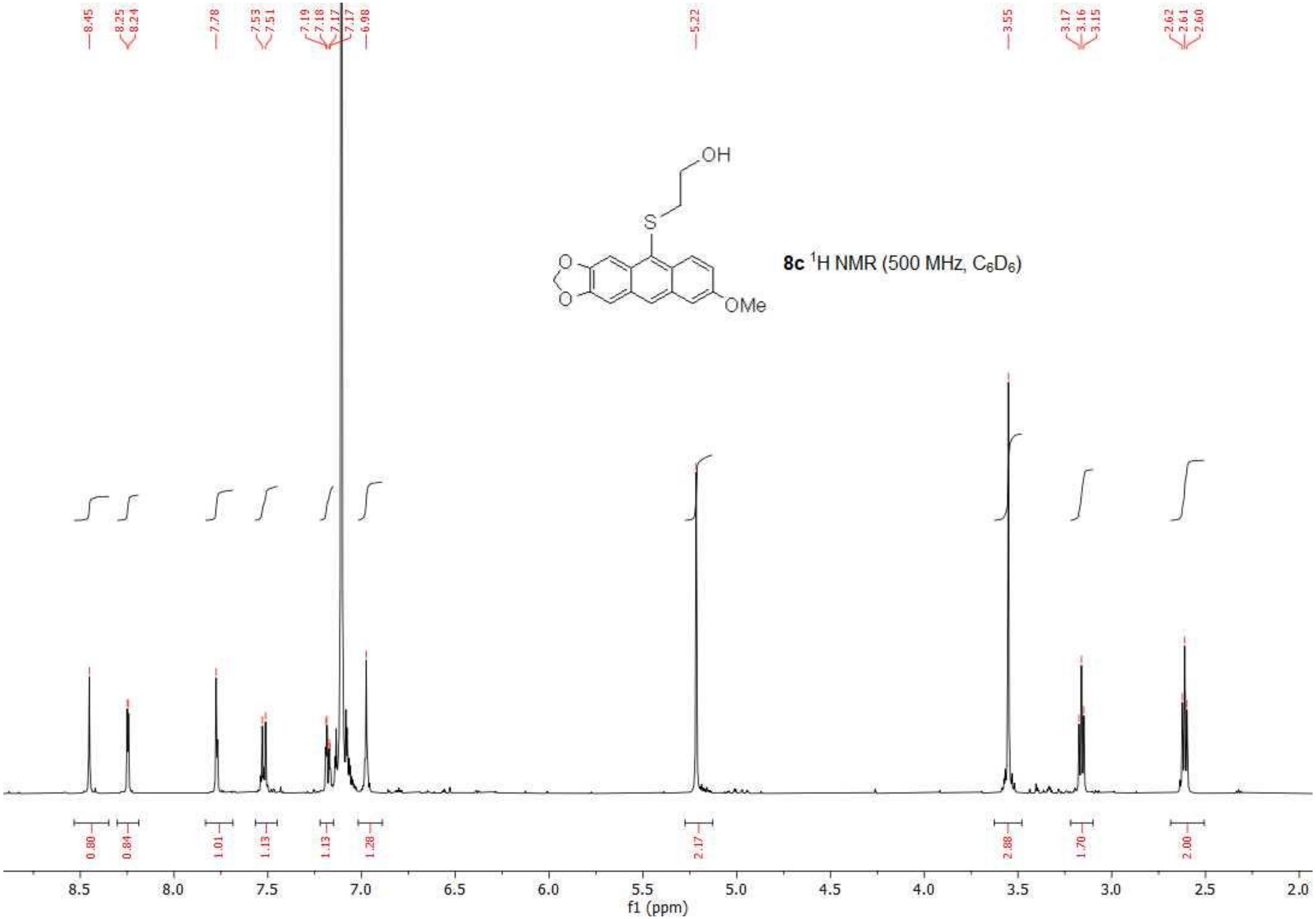

8c ^{1}H NMR (500 MHz, C_6D_6)

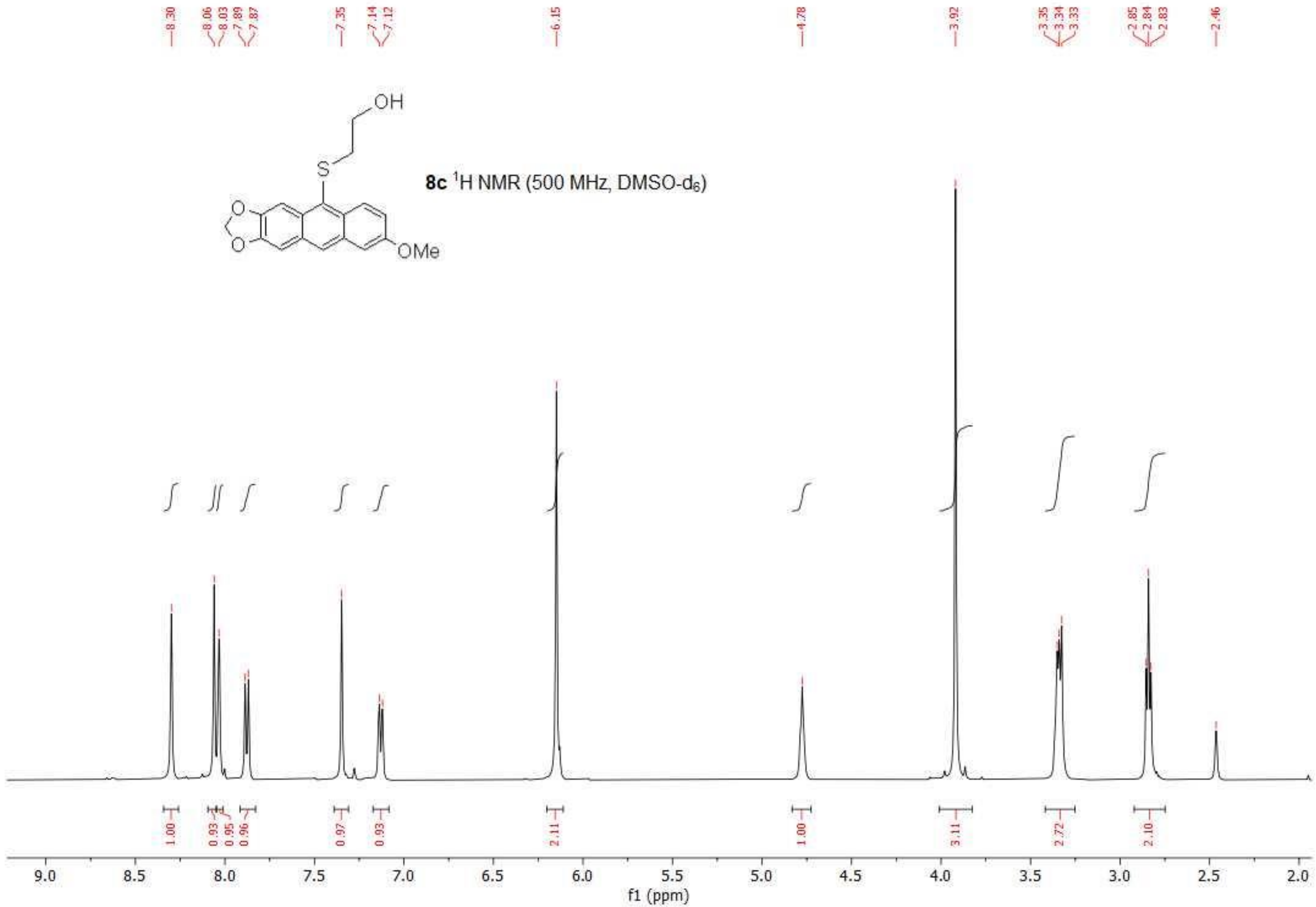
8c ¹H NMR (500 MHz, DMSO-d6)
OH
S
O
O
OMe
8.30
8.06
8.03
7.89
7.87
7.35
7.14
7.12
6.15
4.78
3.92
3.35
3.34
3.33
2.85
2.84
2.83
2.46
1.00
0.93
0.95
0.96
0.97
0.93
2.11
1.00
3.11
2.72
2.10
9.0
8.5
8.0
7.5
7.0
6.5
6.0
5.5
5.0
4.5
4.0
3.5
3.0
2.5
2.0
f1 (ppm)

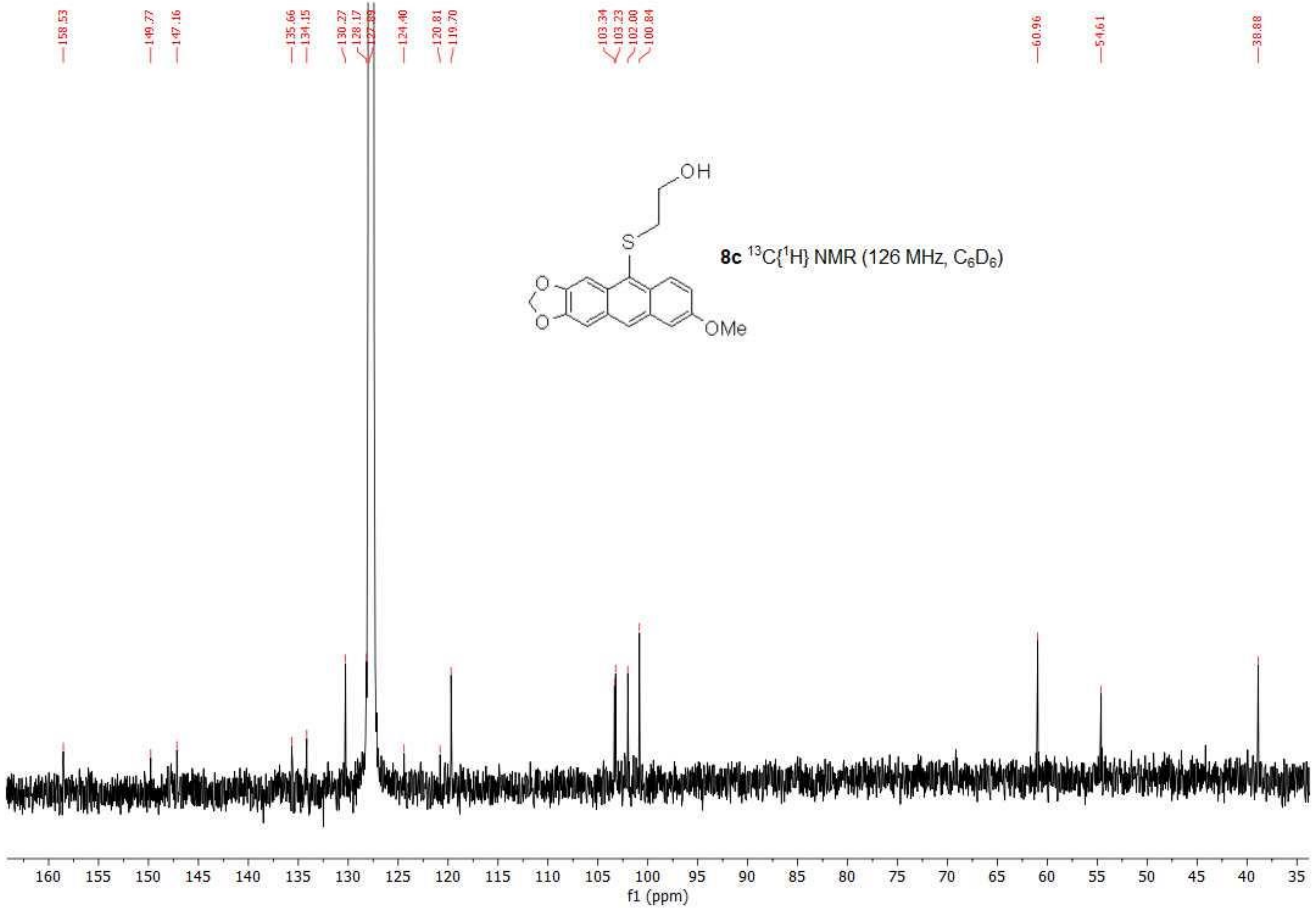

8c $^{13}C\{^{1}H\}$ NMR (126 MHz, C_6D_6)

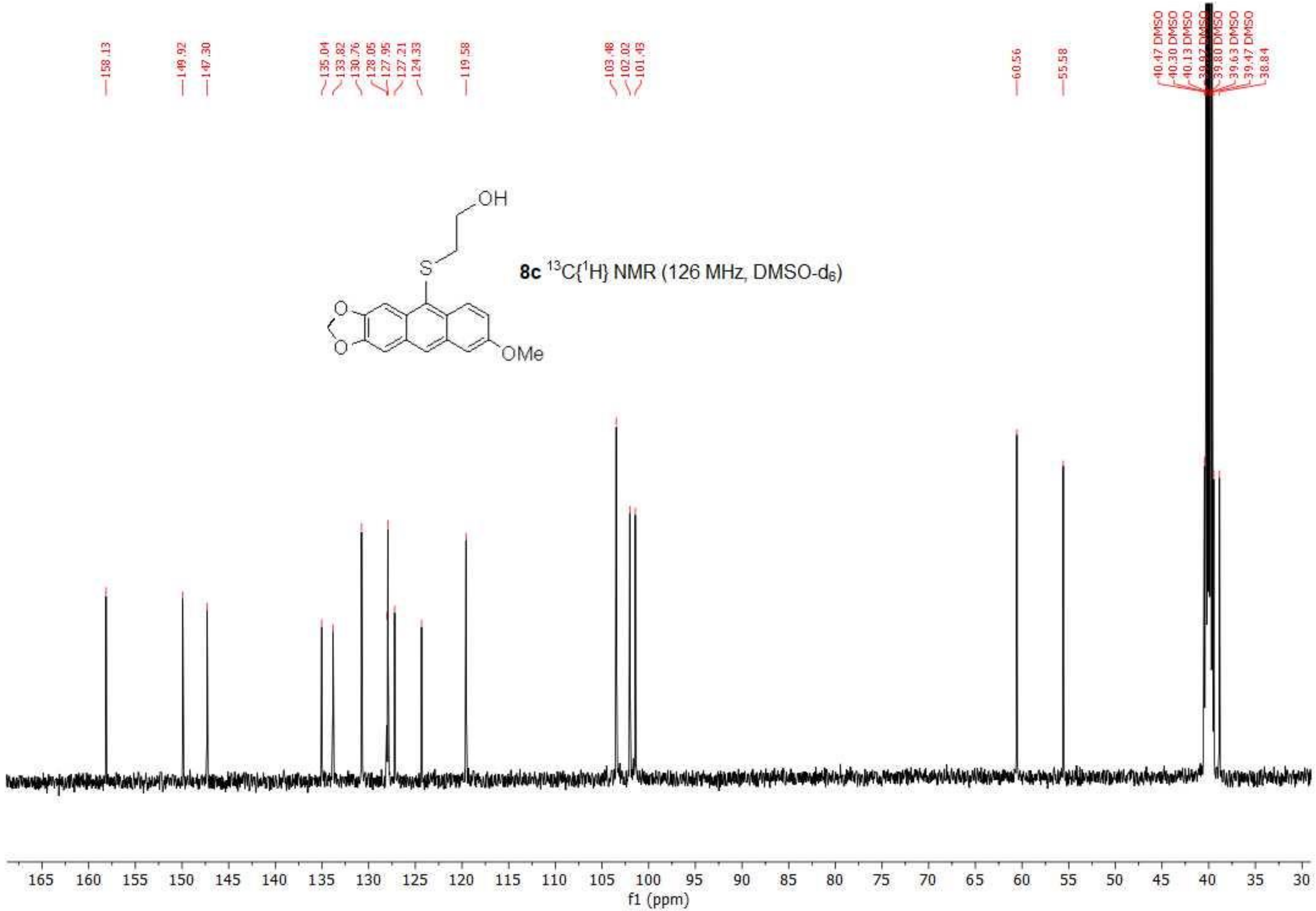

158.13
149.92
147.30
135.04
133.82
130.76
128.05
127.95
127.21
124.33
119.58
103.48
102.02
101.43
60.56
55.58
40.47 DMSO
40.30 DMSO
40.13 DMSO
39.97 DMSO
39.80 DMSO
39.63 DMSO
39.47 DMSO
38.84
OH
S
O
O
OMe
8c $^{13}C\{^{1}H\}$ NMR (126 MHz, DMSO-d_6)
165 160 155 150 145 140 135 130 125 120 115 110 105 100 95 90 85 80 75 70 65 60 55 50 45 40 35 30
f1 (ppm)

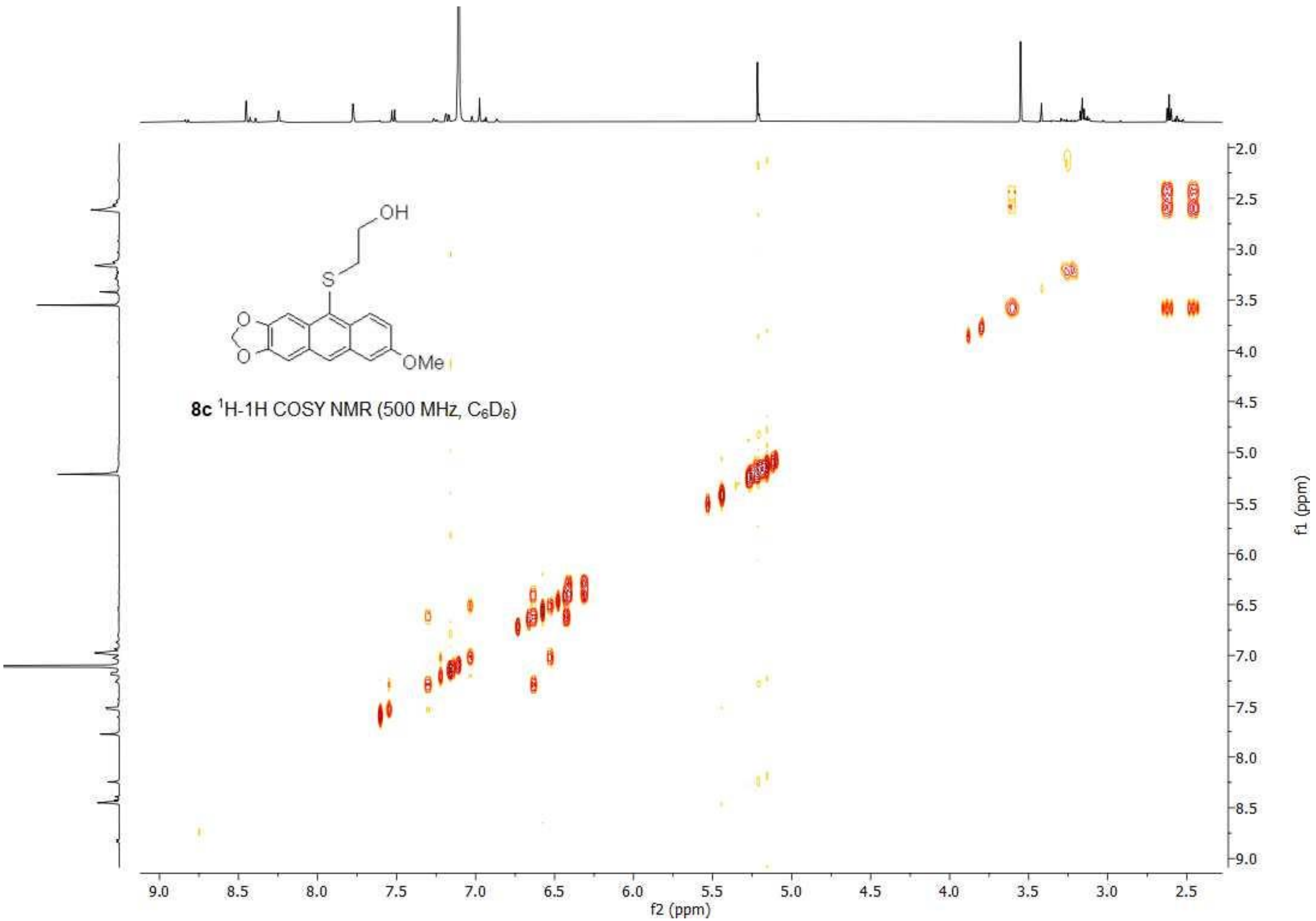

8c ^{1}H-1H COSY NMR (500 MHz, C_6D_6)

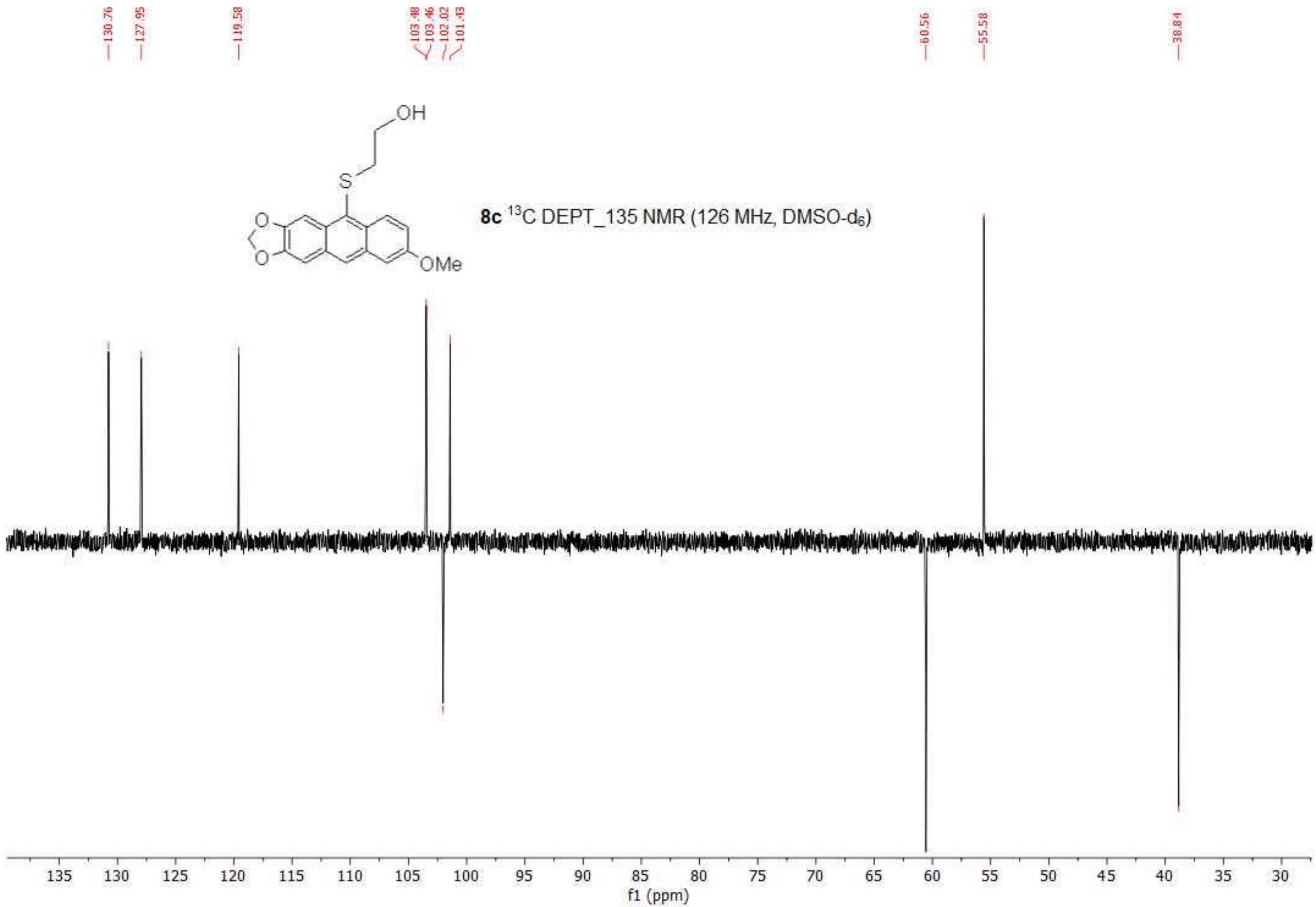
8c ^{13}C DEPT_135 NMR (126 MHz, DMSO-d_6)
OH
S
O
O
OMe
130.76
127.95
119.58
103.48
103.46
102.02
101.43
60.56
55.58
38.84
135 130 125 120 115 110 105 100 95 90 85 80 75 70 65 60 55 50 45 40 35 30
f1 (ppm)

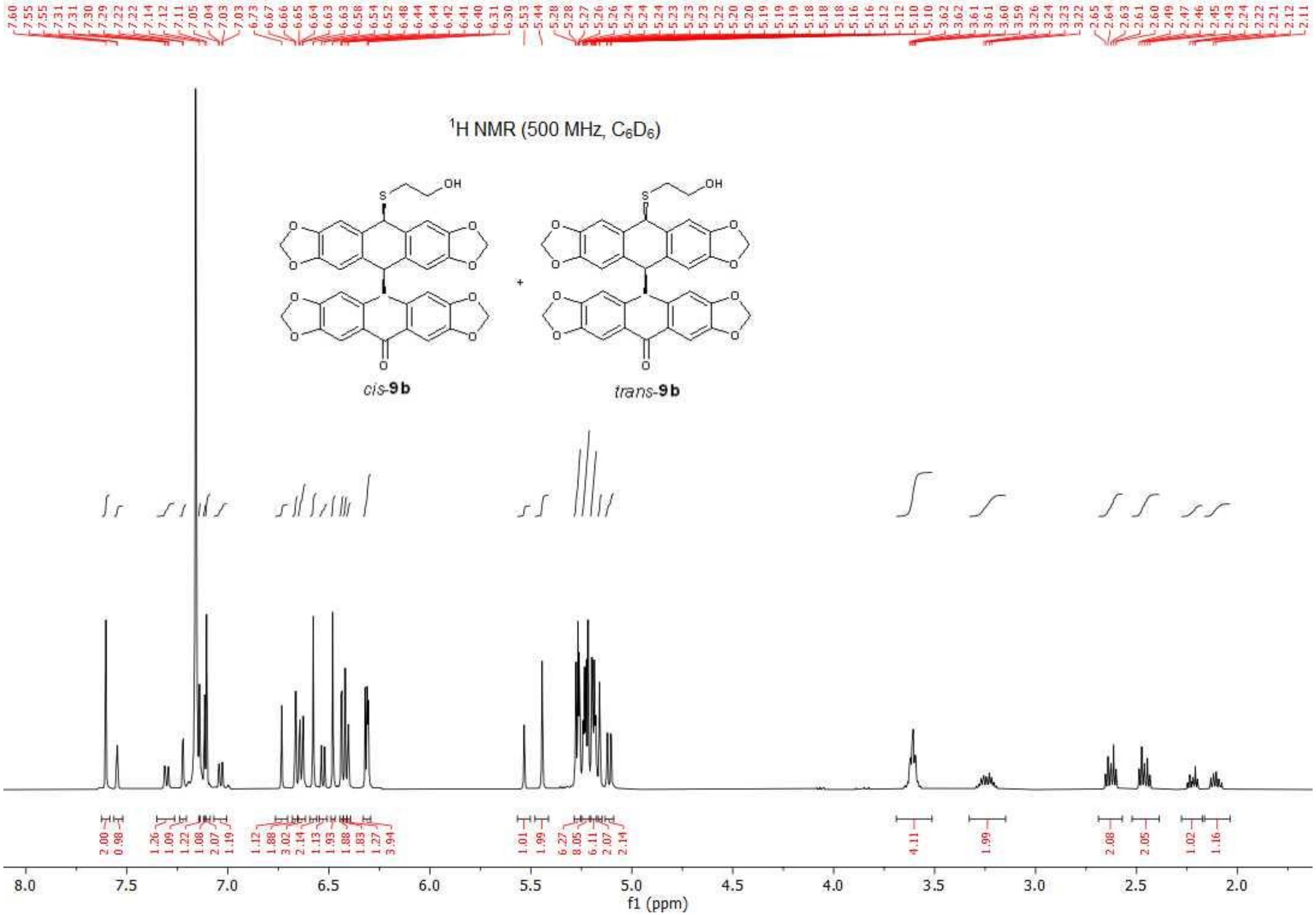

7.60 7.55 7.31 7.31 7.30 7.29 7.22 7.22 7.14 7.12 7.11 7.05 7.04 7.03 7.03 6.73 6.67 6.66 6.65 6.64 6.63 6.63 6.58 6.54 6.52 6.48 6.44 6.44 6.42 6.41 6.40 6.31 6.30 5.53 5.44 5.28 5.28 5.27 5.26 5.26 5.24 5.24 5.24 5.23 5.23 5.23 5.22 5.20 5.20 5.19 5.19 5.19 5.18 5.18 5.16 5.12 5.12 5.10 5.10 3.62 3.62 3.61 3.61 3.60 3.59 3.26 3.24 3.23 3.22 2.65 2.64 2.63 2.61 2.60 2.49 2.47 2.46 2.45 2.43 2.24 2.22 2.21 2.12 2.11
1H NMR (500 MHz, C6D6)
OH
S
O
cis-9b
trans-9b
2.00 0.98 1.26 1.09 1.22 1.08 2.07 1.19 1.12 1.88 3.02 2.14 1.13 1.93 1.88 1.83 1.27 3.94 1.01 1.99 6.27 8.05 6.11 2.07 2.14 4.11 1.99 2.08 2.05 1.02 1.16
8.0 7.5 7.0 6.5 6.0 5.5 5.0 4.5 4.0 3.5 3.0 2.5 2.0
f1 (ppm)

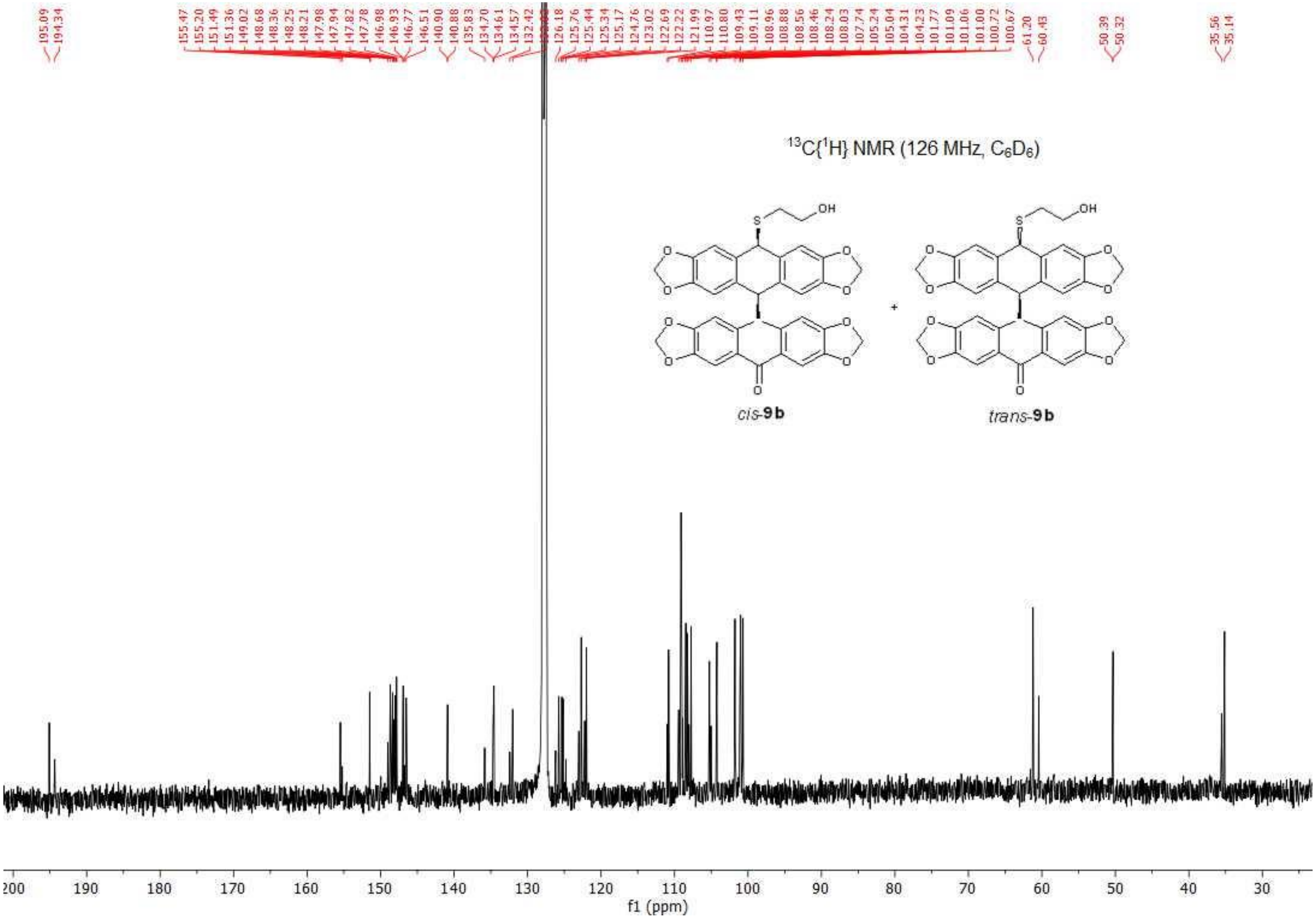
195.09
194.34
155.47
155.20
151.49
151.36
149.02
148.68
148.36
148.25
148.21
147.98
147.94
147.82
147.78
146.98
146.93
146.77
146.51
140.90
140.88
135.83
134.70
134.61
134.57
132.42
126.18
125.76
125.44
125.34
125.17
124.76
123.02
122.69
122.22
121.99
110.97
110.80
109.43
109.11
108.96
108.88
108.56
108.46
108.24
108.03
107.74
105.24
105.04
104.31
104.23
101.77
101.09
101.06
101.00
100.72
100.67
61.20
60.43
50.39
50.32
35.56
35.14
$^{13}C\{^1H\}$ NMR (126 MHz, C_6D_6)
S
OH
O
+
cis-**9b**
trans-**9b**
f1 (ppm)

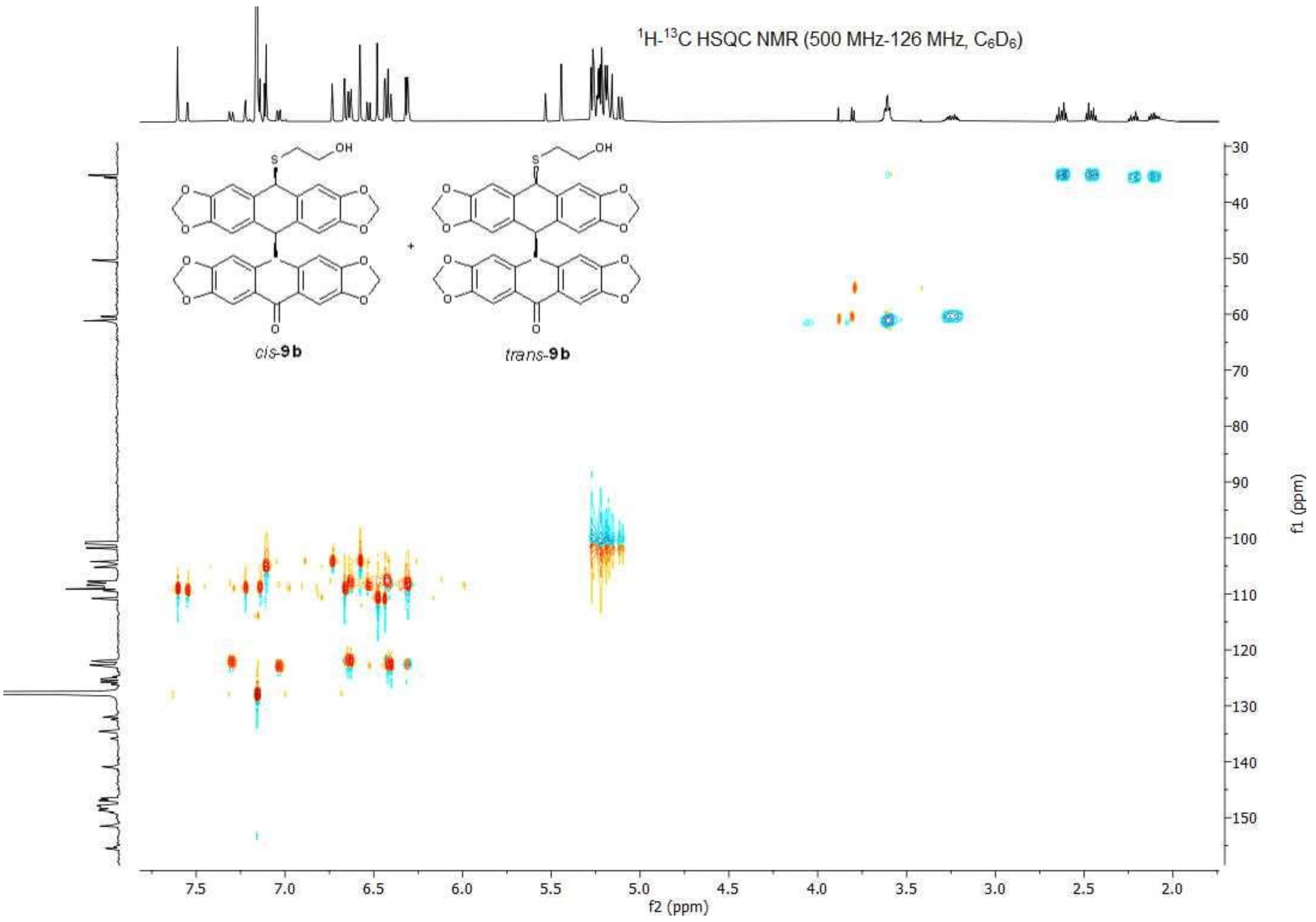

^1H-^{13}C HSQC NMR (500 MHz-126 MHz, C_6D_6)
cis-9b
trans-9b
f2 (ppm)
f1 (ppm)

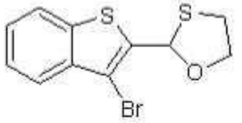

11 ^{1}H NMR (200 MHz, C_6D_6)

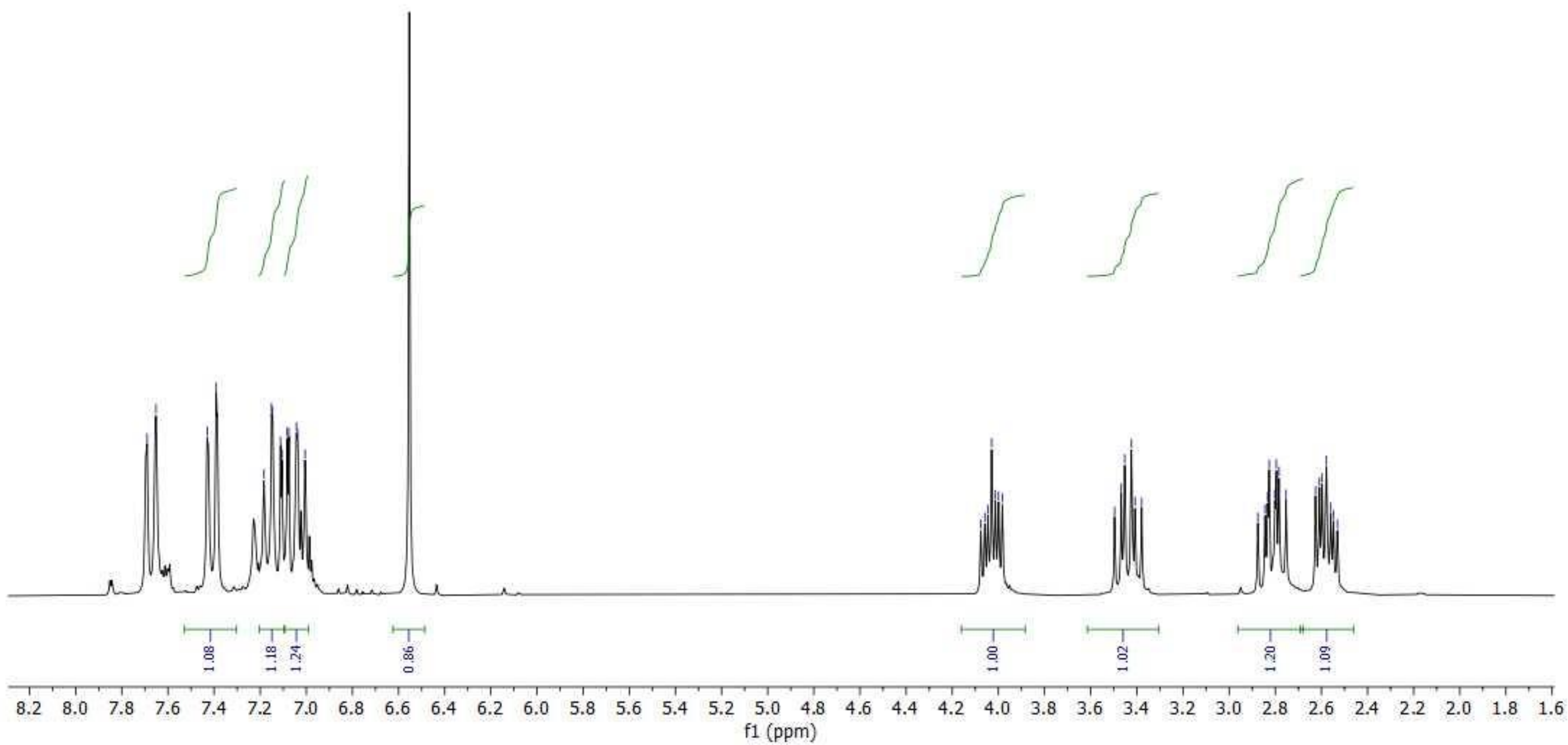

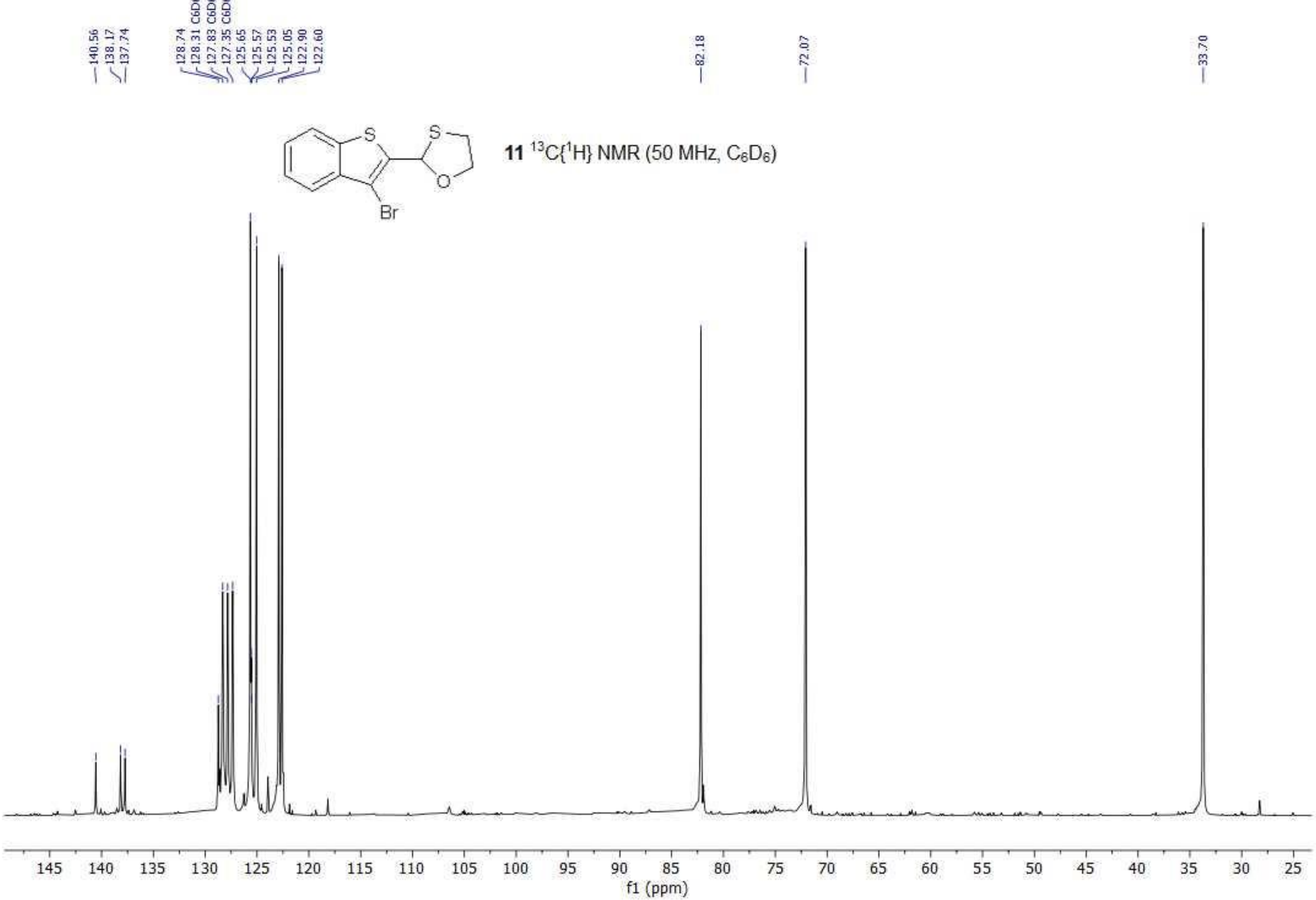
140.56
138.17
137.74
128.74
128.31 C6D6
127.83 C6D6
127.35 C6D6
125.65
125.57
125.53
125.05
122.90
122.60
82.18
72.07
33.70
S
S
O
Br
11 $^{13}C\{^1H\}$ NMR (50 MHz, C_6D_6)
145 140 135 130 125 120 115 110 105 100 95 90 85 80 75 70 65 60 55 50 45 40 35 30 25
f1 (ppm)

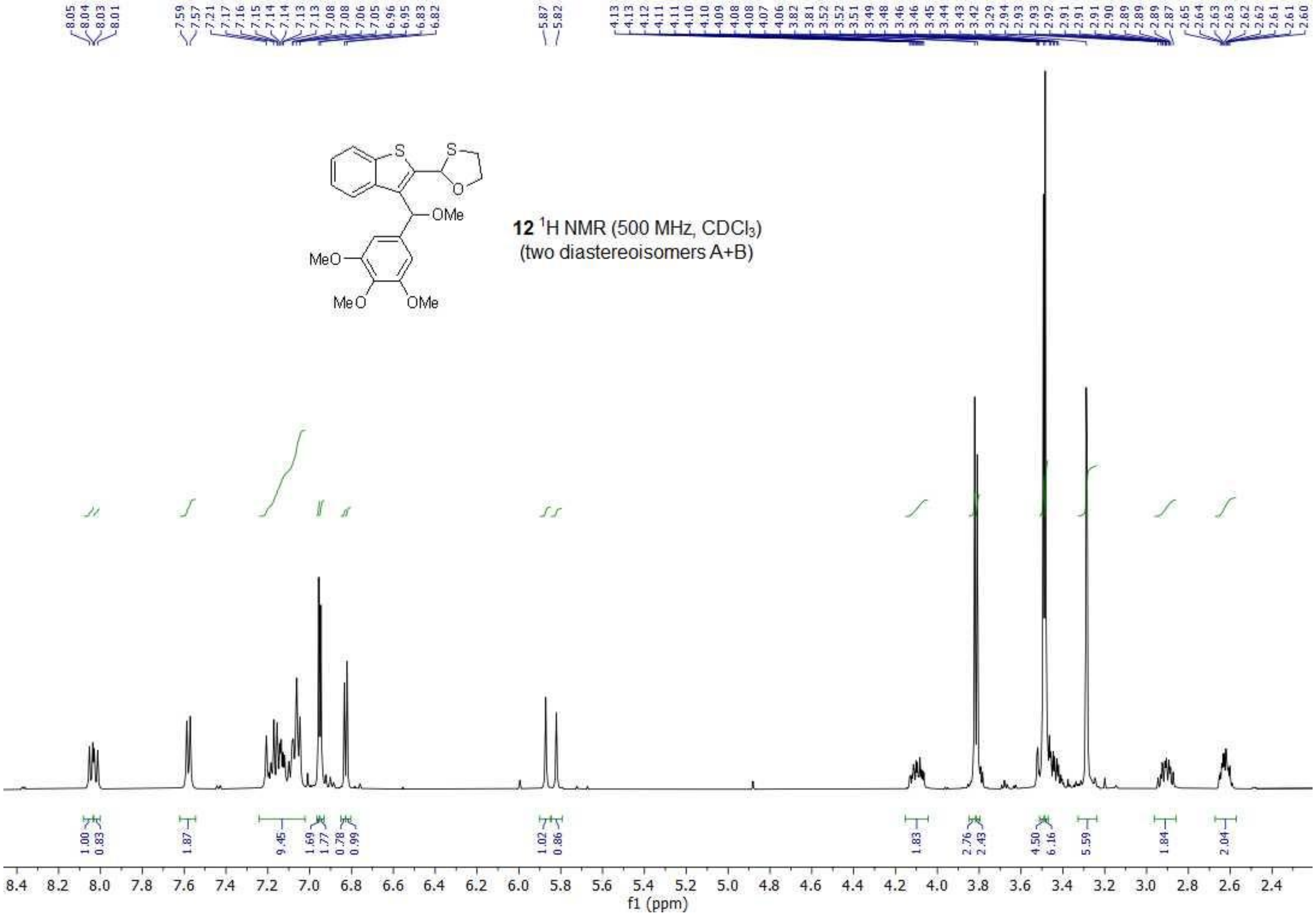
MeO
MeO
OMe
OMe
S
S
O
12 ¹H NMR (500 MHz, CDCl₃)
(two diastereoisomers A+B)
f1 (ppm)

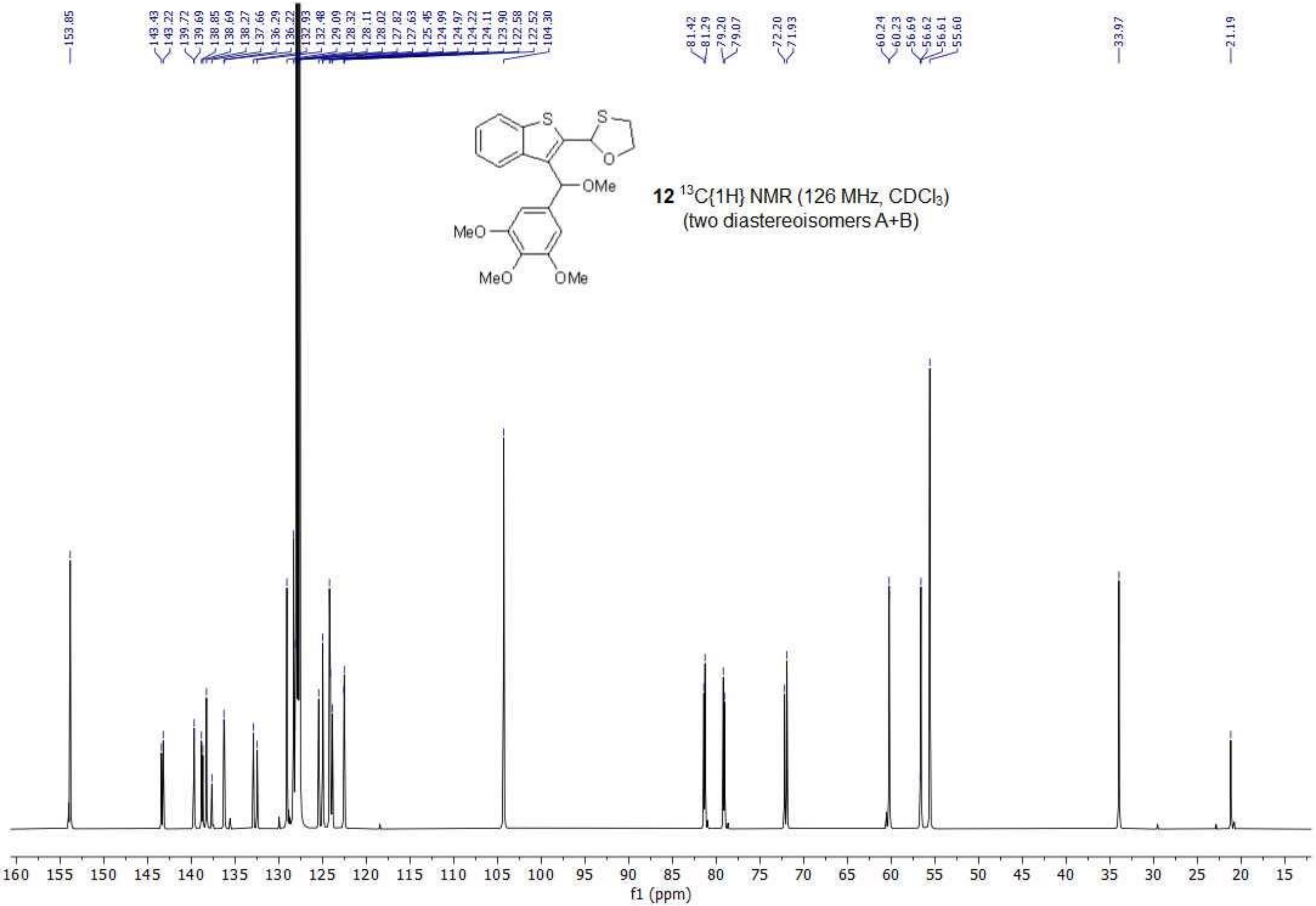

12 $^{13}C\{^1H\}$ NMR (126 MHz, $CDCl_3$)
(two diastereoisomers A+B)

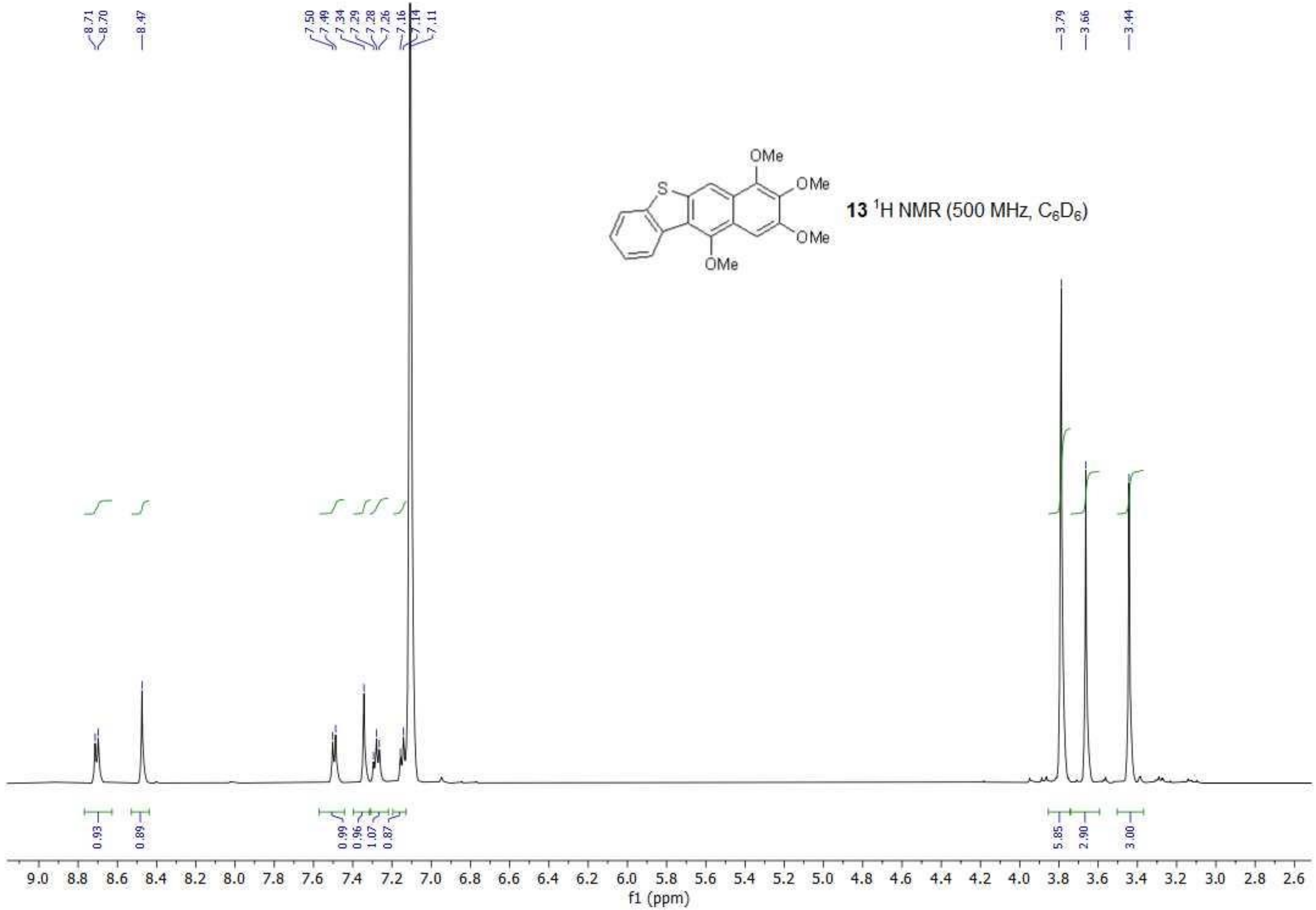
13 ¹H NMR (500 MHz, C_6D_6)
OMe
OMe
OMe
OMe
S
8.71
8.70
8.47
7.50
7.49
7.34
7.29
7.28
7.26
7.16
7.14
7.11
3.79
3.66
3.44
0.93
0.89
0.99
0.96
1.07
0.87
5.85
2.90
3.00
9.0 8.8 8.6 8.4 8.2 8.0 7.8 7.6 7.4 7.2 7.0 6.8 6.6 6.4 6.2 6.0 5.8 5.6 5.4 5.2 5.0 4.8 4.6 4.4 4.2 4.0 3.8 3.6 3.4 3.2 3.0 2.8 2.6
f1 (ppm)

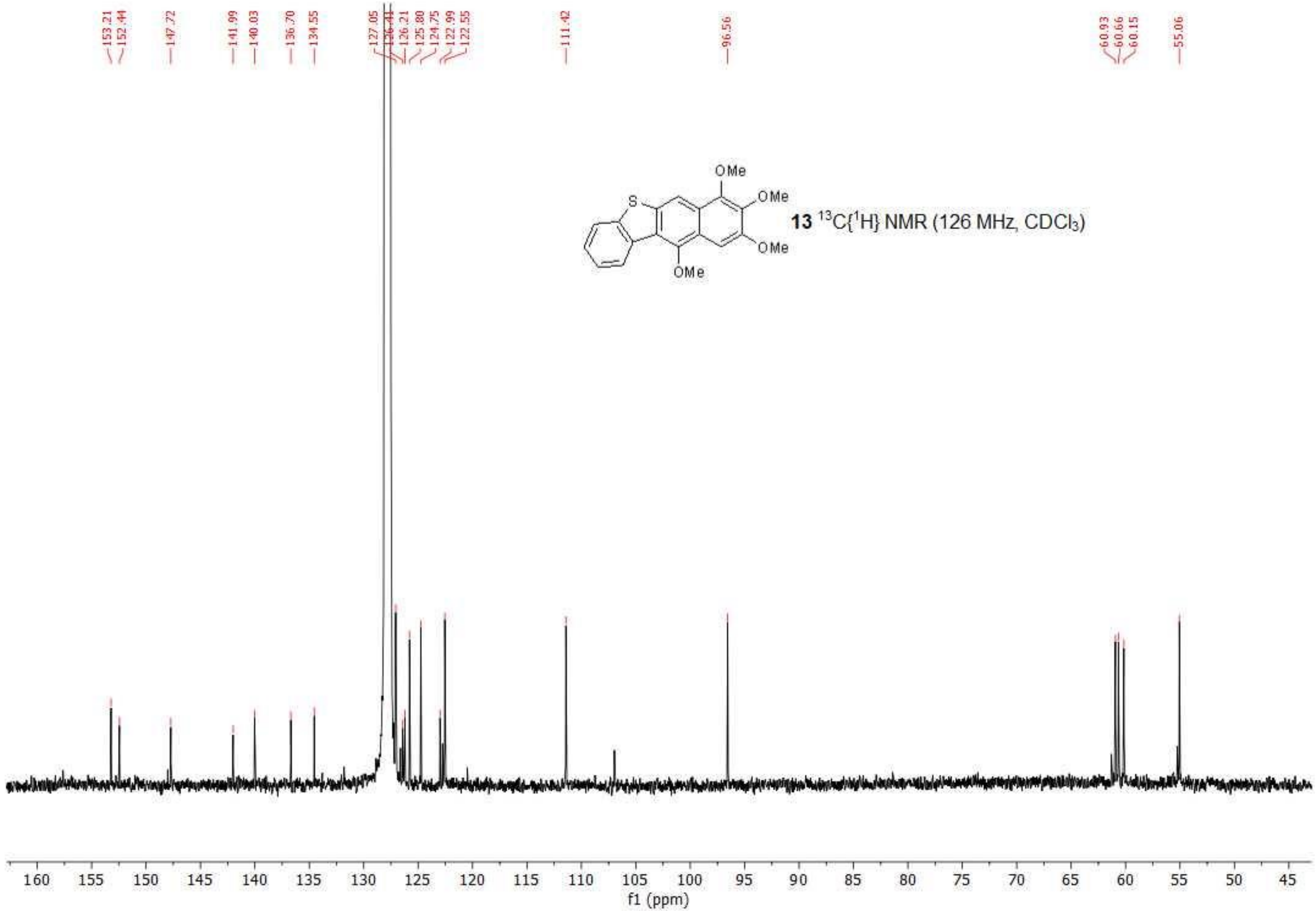
153.21
152.44
147.72
141.99
140.03
136.70
134.55
127.05
126.41
126.21
125.80
124.75
122.99
122.55
111.42
96.56
60.93
60.66
60.15
55.06
OMe
OMe
OMe
OMe
S
13 13C{1H} NMR (126 MHz, CDCl3)
160 155 150 145 140 135 130 125 120 115 110 105 100 95 90 85 80 75 70 65 60 55 50 45
f1 (ppm)

Crystal structure determination

X-ray diffraction data for **13** were collected using an Oxford Diffraction Xcalibur Saphire 3 diffractometer with CuKα radiation. The structures were solved by direct methods and refined by full-matrix least-squares on F^2 with SHELXL-2014 [1]. The carbon, oxygen and sulphur atoms were refined anisotropically. All aromatic H atoms and H atoms of methyl groups were positioned geometrically and constrained to ride on their parent atoms, with C–H distances of 0.93 and 0.96 Å, respectively, and with $U_{iso}(H_{aromatic})$ and $U_{iso}(H_{methyl})$ values of 1.2 $U_{eq}(C_{aromatic})$ and 1.5 $U_{eq}(C_{methyl})$. Details of data collections and structure refinements have been deposited with the Cambridge Crystallographic Data Centre as supplementary publication number CCDC 1475147.

Calculations

The molecular and electronic structures of *cis*-**9b**, *trans*-**9b, 5b, 16a** and **16b** were calculated by DFT method using the gradient corrected three-parameter hybrid functional (B3LYP) with the 6-31++G(d,p) (for *cis*-**9b** and *trans*-**9b**) and 6-311++G(d,p) basis sets (for **5b, 16a** and **16b**). Full geometry optimizations of compounds in the gas phase were performed using its starting geometries prepared manually. The above computations were performed using GAUSSIAN09 quantum chemistry package. In order to check the structural optimizations, the calculated vibrational frequencies of the compounds were used (no imaginary frequencies). GAUSSIAN09 was also used for the TD-DFT (B3LYP 6-311++G(d,p) calculations performed for **7b**.

The calculations in the framework of the noncovalent interactions (NCI) method [2] were performed with the Multiwfn (A Multifunctional Wavefunction Analyzer) software [3]. This method is based on the relationship between the electron density (ρ) and the reduced density gradient ($RDG = \frac{1}{2(3\pi^2)^{1/3}}\frac{|\nabla\rho|}{\rho^{4/3}}$), and is a very useful tool for the analysis and visualization of different types of non-covalent interactions. Chemcraft program [4] was employed to visualize the chemical structures and molecular orbitals of investigated compounds, and VMD (Visual Molecular Dynamics) [5] and Multiwfn software[3] were used for visualization of their RDG isosurfaces. The latter software was also used to perform the quantitative analysis of molecular surfaces of **5b**, **16a** and **16b**.

Cartesian coordinates and total energies for the geometries of *cis*-9b and *trans*-9b optimized using Gaussian 09

Table S1. Atom coordinates (Å), total energy (Hartree) and the number of imaginary vibrational frequencies for the geometry of *cis*-**9b** optimized at the B3LYP/6-31++(d,p) level in the gas phase using Gaussian 09.

H	0.515590	-3.776210	0.065460
C	0.343632	-2.702158	0.172555
C	1.458119	-1.952754	-0.513957
C	2.711596	-2.584562	-0.683454
C	3.686621	-1.897029	-1.375815
O	4.954823	-2.311444	-1.717134
C	5.592135	-1.143211	-2.255601
O	4.557124	-0.216292	-2.613136
C	3.446196	-0.636884	-1.915718
C	2.226327	-0.007548	-1.779079
C	1.218969	-0.674027	-1.049490
C	-0.145246	-0.026123	-0.886016
H	-0.293606	0.669623	-1.719168
C	-0.248703	0.890152	0.442795
H	-0.098402	0.217177	1.290334
C	0.844824	1.937684	0.441758
C	2.106780	1.638274	0.993633
C	3.088880	2.603513	0.895589
O	4.387451	2.537380	1.333082
C	4.917479	3.863254	1.156089
O	4.036859	4.562899	0.271000
C	2.870259	3.827772	0.258983
C	1.650537	4.151811	-0.284017
C	0.620561	3.187009	-0.167841
C	-0.745145	3.571878	-0.614353
O	-0.941872	4.568084	-1.312177
C	-1.868956	2.765541	-0.067085
C	-3.163975	3.337923	-0.088908
C	-4.163140	2.639547	0.544603
O	-5.501377	2.952089	0.658712
C	-6.030364	2.015544	1.602807

O	-5.083910	0.938238	1.718412
C	-3.917295	1.420193	1.180685
C	-2.668721	0.831148	1.188482
C	-1.622367	1.519953	0.542032
C	-1.232575	-1.086015	-0.956317
C	-2.459991	-0.789507	-1.589722
C	-3.422166	-1.776138	-1.618430
O	-4.667379	-1.745273	-2.206182
C	-5.324375	-2.935360	-1.748122
O	-4.308809	-3.829289	-1.268973
C	-3.206361	-3.031845	-1.054174
C	-2.009735	-3.357962	-0.454105
C	-1.007661	-2.362685	-0.415305
H	2.894765	-3.587067	-0.311227
H	6.157921	-1.417541	-3.147251
H	6.233885	-0.692429	-1.485274
H	2.047379	0.966745	-2.220707
H	2.301949	0.683485	1.468216
H	5.910126	3.797603	0.707052
H	4.943104	4.372132	2.129634
H	1.449728	5.101447	-0.765779
H	-3.324082	4.294306	-0.572550
H	-6.980078	1.623137	1.234916
H	-6.141844	2.501703	2.581949
H	-2.503630	-0.128019	1.666001
H	-2.641416	0.182532	-2.035123
H	-5.853837	-3.401120	-2.580754
H	-6.003149	-2.680665	-0.921771
H	-1.837565	-4.344662	-0.037183
S	0.229749	-2.404550	2.039398
C	1.838711	-3.014481	2.667770
H	1.963566	-2.548650	3.650130
H	2.650103	-2.653476	2.029498
C	1.900535	-4.530868	2.809400
H	1.718896	-5.014925	1.838528
H	1.122433	-4.868928	3.506735
O	3.206512	-4.852774	3.295439
H	3.264511	-5.804579	3.447583

Total energy	-2460.004177
Number of imaginary vibrational frequencies	0

Table S2. Atom coordinates (Å), total energy (Hartree) and the number of imaginary vibrational frequencies for the geometry of *trans*-**9b** optimized at the B3LYP/6-31++(d,p) level in the gas phase using Gaussian 09.

Atom	X	Y	z
O	-4.912202	1.585518	1.488486
C	-3.669161	1.935627	1.027387
C	-2.506630	1.192307	1.065503
C	-1.348807	1.765122	0.500745
C	-1.235952	-0.860491	-1.013824
C	-2.360346	-0.466105	-1.767963
C	-3.430894	-1.334156	-1.821816
O	-4.613102	-1.205602	-2.517457
C	-5.439667	-2.277734	-2.040875
O	-4.579070	-3.238989	-1.413818
C	-3.409092	-2.555675	-1.158781
C	-2.315876	-2.975082	-0.427552
C	-1.203111	-2.109713	-0.364602
H	2.534566	-3.777185	-0.069751
H	6.010289	-2.269314	-3.076157
H	6.191110	-1.424986	-1.480130
H	2.235703	0.684545	-2.331496
H	2.427564	0.372689	1.468983
H	6.431798	2.968457	0.826042
H	5.565748	3.625461	2.277419
H	2.214663	4.944332	-0.598153
H	-2.627630	4.774882	-0.564861
H	-6.677076	2.512083	0.916791
H	-5.839417	3.227924	2.356914
H	-2.494049	0.198912	1.499580
H	-2.384059	0.489197	-2.281215
H	-5.951339	-2.744048	-2.884378
H	-6.149319	-1.885943	-1.297878
H	-2.311932	-3.938039	0.066906
S	0.130985	-4.240127	0.933142
C	0.932765	-4.070302	2.575122
H	1.405111	-5.035133	2.781030
H	1.728302	-3.321076	2.524805
C	-0.052272	-3.729222	3.687147

H	-0.590270	-2.800331	3.447968
H	-0.793870	-4.533789	3.782469
O	0.707958	-3.580372	4.888691
H	0.107642	-3.422282	5.628365
H	0.072027	-1.911156	1.341034
Total energy		-2459.996890	
Number of imaginary vibrational frequencies		0	

Table S3. Atom coordinates (Å), total energy (Hartree) and the number of imaginary vibrational frequencies for the geometry of **5b** optimized at the B3LYP/6-311++(d,p) level in the gas phase using Gaussian 09.

Atom	X	Y	Z
O	4.003551	-2.887588	-0.068798
C	5.127414	-2.263804	-0.706813
H	5.532462	-2.934285	-1.463405
H	5.875831	-2.005209	0.052782
O	4.652318	-1.066696	-1.332568
C	2.672433	0.331054	-0.808993
H	2.975369	1.182700	-1.402091
C	1.445439	0.345175	-0.114939
C	1.043898	-0.763506	0.649892
C	1.873879	-1.901510	0.732739
H	1.578800	-2.762824	1.319850
C	3.062110	-1.892791	0.040291
C	3.451237	-0.795764	-0.717793
C	0.600219	1.586782	-0.229141
H	-0.406597	1.429895	0.151837
O	0.562767	2.027703	-1.567803
C	0.094205	3.368642	-1.607538
H	-0.958877	3.411159	-1.300240
H	0.178892	3.703620	-2.642345
C	0.962915	4.192843	-0.649270
H	1.902274	4.490270	-1.116726
H	0.436677	5.079701	-0.294632
S	1.299917	3.056795	0.757260
C	-0.275931	-0.810897	1.418706
H	-0.253360	-1.730777	2.024343
O	-0.430789	0.290345	2.314258
C	0.443556	0.260736	3.432332
H	0.199582	1.131307	4.040564
H	0.292769	-0.651628	4.025762
H	1.495547	0.318601	3.130571
C	-1.525518	-0.881328	0.540590
C	-1.487787	-1.498469	-0.711344
C	-2.745479	-0.388345	1.048176
C	-2.640297	-1.648613	-1.500370
H	-0.545988	-1.868306	-1.096071

C	-3.864526	-0.543726	0.261968
H	-2.788466	0.107058	2.007582
C	-3.819070	-1.160020	-0.983350
H	-2.600974	-2.116002	-2.475894
O	-5.068580	-1.126733	-1.559908
O	-5.146951	-0.103114	0.506550
C	-5.948761	-0.726500	-0.502630
H	-6.673995	-0.009921	-0.885519
H	-6.436752	-1.615183	-0.081437
Total energy	-1584.317859		
Number of imaginary vibrational frequencies	0		

Table S4. Atom coordinates (Å), total energy (Hartree) and the number of imaginary vibrational frequencies for the geometry of **16a** optimized at the B3LYP/6-311++(d,p) level in the gas phase using Gaussian 09.

Atom	X	Y	Z
C	3.976628	-0.894226	-0.200932
C	2.945527	-1.590379	0.387051
C	1.662135	-1.017971	0.329827
C	1.455522	0.213924	-0.295718
C	2.524757	0.912110	-0.885020
C	3.770572	0.330929	-0.824529
C	0.075109	0.829343	-0.301008
C	0.505287	-1.732794	0.991458
C	-1.013767	-0.224323	-0.349756
C	-0.809819	-1.458362	0.283395
C	-1.796146	-2.459745	0.233845
H	-1.635335	-3.431859	0.684140
C	-2.967432	-2.173515	-0.433692
C	-3.166976	-0.954115	-1.065707
C	-2.205176	0.033057	-1.053162
H	3.116905	-2.538341	0.881829
H	2.376190	1.873248	-1.361627
H	-2.357449	0.962873	-1.587991
H	-0.024191	1.473587	-1.183886
H	0.689707	-2.817199	0.963078
C	-1.091952	2.550765	0.983801

H	-2.019332	2.088928	0.626666
H	-1.214613	2.751930	2.050320
C	-0.815554	3.859980	0.241327
H	0.052929	4.350541	0.680749
H	-0.625422	3.676141	-0.817109
O	0.507737	-1.324241	2.370385
C	-0.342288	-2.075113	3.217959
H	-0.148789	-1.729337	4.233816
H	-1.402660	-1.919886	2.985736
H	-0.120118	-3.151249	3.159449
O	5.312107	-1.218593	-0.255063
O	4.969513	0.816746	-1.291617
C	5.887597	-0.277066	-1.169185
H	6.833401	0.085893	-0.769079
H	6.015753	-0.754303	-2.149080
O	-4.048245	-2.992460	-0.662042
O	-4.378532	-0.965041	-1.715367
C	-5.048892	-2.133089	-1.224553
H	-5.542870	-2.642596	-2.050368
H	-5.758601	-1.841396	-0.440185
O	0.005182	1.652553	0.887332
S	-2.293894	4.958642	0.401606
H	-1.746105	6.036573	-0.193585
Total energy		-1584.320012	
Number of imaginary vibrational frequencies		0	

Table S5. Atom coordinates (Å), total energy (Hartree) and the number of imaginary vibrational frequencies for the geometry of **16b** optimized at the B3LYP/6-311++(d,p) level in the gas phase using Gaussian 09.

Atom	X	Y	Z
C	3.743403	0.332348	-0.843920
C	2.527987	0.974423	-0.822405
C	1.436609	0.281035	-0.262365
C	1.601248	-1.005710	0.258503
C	2.856959	-1.641020	0.230555
C	3.905342	-0.949852	-0.329331
C	0.077881	0.940321	-0.228198

C	0.431888	-1.708818	0.907022
C	-0.884779	-1.336064	0.251651
C	-1.053766	-0.051340	-0.285598
C	-2.255571	0.301531	-0.930945
H	-2.391365	1.275808	-1.383835
C	-3.251286	-0.648513	-0.993307
C	-3.084818	-1.918143	-0.458411
C	-1.912289	-2.292802	0.159424
H	2.416119	1.978304	-1.212882
H	2.992926	-2.632652	0.643955
H	-1.783427	-3.295349	0.549154
H	-0.002310	1.630049	-1.069392
H	0.567753	-2.796895	0.818561
S	0.037539	2.020398	1.321258
C	-1.465994	3.033282	1.074093
H	-2.304450	2.396861	0.784931
H	-1.696324	3.436587	2.063012
C	-1.286541	4.176962	0.086689
H	-0.497761	4.849775	0.442845
H	-0.984011	3.793511	-0.896801
O	-2.541549	4.858064	-0.008778
H	-2.439676	5.621758	-0.584779
O	0.490869	-1.371287	2.305019
C	-0.399753	-2.094342	3.135775
H	-0.165390	-1.808290	4.161419
H	-1.448514	-1.851214	2.929704
H	-0.257756	-3.179989	3.026699
O	4.952302	0.798616	-1.304133
O	5.222175	-1.329035	-0.447146
C	5.819375	-0.342701	-1.297501
H	5.900160	-0.742298	-2.316392
H	6.791113	-0.057856	-0.896355
O	-4.509945	-0.537332	-1.534323
O	-4.236158	-2.649555	-0.639915
C	-5.027199	-1.874613	-1.550002
H	-6.063448	-1.866789	-1.215022
H	-4.928007	-2.289357	-2.561093
Total energy		-1584.330880	
Number of imaginary vibrational frequencies		0	

References

1. G. M. Sheldrick, Crystal structure refinement with *SHELXL,* Acta Crystallographica C71 (2015) 3-8.
2. E. R. Johnson, S. Keinan, P. Mori-Sanchez, J. Contreras-Garcia, A.J. Cohen, W. Yang, Revealing noncovalent interactions, Journal of the American Chemical Society 132 (2010) 6498–6506.
3. T. Lu, F. Chen, Multiwfn: A multifunctional wavefunction analyzer, Journal Compututational Chemistry, 33 (2012) 580–592.
4. Chemcraft - graphical software for visualization of quantum chemistry computations. https://www.chemcraftprog.com
5. W. Humphrey, A. Dalke, K. Schulten, VMD - Visual Molecular Dynamics, Journal of Molecular Graphics 14 (1996) 33-38
